# Synthesis and Anti-Neuroinflammatory Activity of 1,7-diphenyl-1,4-heptadien-3-ones in LPS-Stimulated BV2 Microglia Via Inhibiting NF-*κ*B/MAPK Signaling Pathways

**DOI:** 10.3390/molecules27113537

**Published:** 2022-05-31

**Authors:** Xuan Zhao, Jiqing Fang, Yu Jia, Zi Wu, Meihui Zhang, Mingyu Xia, Jinhua Dong

**Affiliations:** 1 Key Laboratory of Structure-Based Drug Design & Discovery, Ministry of Education, Shenyang Pharmaceutical University, 103 Wenhua Road, Shenhe District, Shenyang City 110016, China; zhaoxuan666jy@163.com (X.Z.); jy666zhaoxuan@163.com (Y.J.); zhangmeihui2005@hotmail.com (M.Z.); 2 School of Life Science and Biopharmaceutics, Shenyang Pharmaceutical University, 103 Wenhua Road, Shenhe District, Shenyang City 110016, China; fangjiqing1998@163.com (J.F.); hildawuzi@163.com (Z.W.)

**Keywords:** curcumin, 1,7-diphenyl-1,4-heptadien-3-one, neuroinflammation, anti-inflammation, BV2 cell, synthesis

## Abstract

A series of 1,7-diphenyl-1,4-heptadien-3-ones with various substituents (HO-, CH_3_O-, CH_3_-, Cl-) on the phenyl rings were synthesized and evaluated for anti-neuroinflammatory effects in LPS-stimulated BV2 microglia. The pharmacological results showed that the target compounds bearing methoxy groups greatly inhibited LPS-induced NO release, and that the active compounds **CU-19** and **CU-21** reduced the level of NO, TNF-*α*, IL-6 and PGE-2, downregulated the expression of COX-2 and iNOS in LPS-stimulated BV2 cells. A study of the mechanism of action revealed that **CU-19** and **CU-21** inhibited the nuclear translocation of NF-*κ*B and phosphorylation of MAPKs (ERK, JNK, and p38). A preliminary pharmacokinetic study in rats revealed that the pharmacokinetic properties of **CU-19** and **CU-21** were dramatically ameliorated in comparison with the pharmacokinetic properties of curcumin.

## 1. Introduction

Neuroinflammation is a significant and complicated pathological process mediating all types of damage and disorders of the central nervous system [1]. Microglia, the major cellular elements of neuroinflammation, influence brain development and maintain the neural environment and responses to brain injury [2]. Activated microglia are characterized by the increased release of neurotoxic proinflammatory mediators, such as nitric oxide (NO), interleukin-6 (IL-6) and tumor necrosis factor-*α* (TNF-*α*) [3,4]. Thus, inhibiting neuroinflammation by suppressing the activation of microglia is a promising therapeutic strategy for the treatment of neuroinflammation-associated diseases [5].

**Figure 1:**
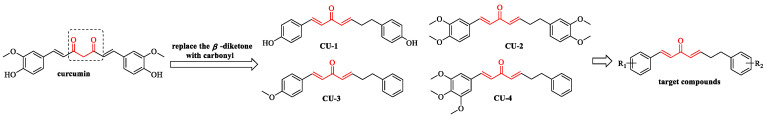
The structures of **CU**, some reported mono-carbonyl analogues, and the target compounds.

The natural product curcumin (**CU**) has the potential to treat neuroinflammation, which inhibits the release of cytokines by inhibiting the MAPK signaling pathway and promotes microglia M2 polarization via TREM2/TLR4/NF-*κ*B signaling pathways [6,7,8,9,10]. Since the *β*-diketone moiety in the skeleton of **CU** is a specific substrate of a series of aldo-keto reductases, **CU** is rapidly metabolized into non-bioactive components in vivo [11,12]. Most likely, the clinical applications of **CU** as an anti-neuroinflammatory agent will be limited by its rapid metabolism as well as poor bioavailability. In order to avoid the metabolic defect of *β*-diketone moiety, great efforts have been made in the structural modification of **CU**, and the mono-carbonyl analogues of **CU** have been the focus of attention in the investigation of curcuminoids in recent two decades. Many mono-carbonyl analogues of **CU** with enhanced biological activities and ameliorated pharmacokinetic properties have been discovered [11,12,13,14,15,16,17]. However, the anti-neuroinflammatory activities of mono-carbonyl analogues have never been reported. 1,7-Diphenyl-1,4-heptadiene-3-ones, such as **CU-1**~**CU-4**, exhibited potent growth inhibitory effects against various tumor cells in vitro [18,19,20] (Figure 1). In order to explore novel anti-neuroinflammatory agents, we synthesized a series of 1,7-diphenyl-1,4-heptadien-3-ones (**CU-1**~**CU-28**, Table 1) and investigated their anti-neuroinflammatory effects.

Lipopolysaccharide (LPS)-stimulated BV2 microglia secrete proinflammatory mediators (NO, TNF-*α*, IL-6). This is a classic cell model for screening anti-neuroinflammatory agents. Herein, we assessed the inhibitory effects of the target compounds on NO release in LPS-stimulated BV2 cells in vitro. The results revealed that compounds **CU-19** and **CU-21** exhibited potent inhibition of NO release. Hence, these two compounds were further studied for understanding the mechanism of action and metabolic characteristics.

## 2. Results and Discussions

### 2.1. Chemistry

The synthesis of 1,7-diphenyl-1,4-heptadien-3-ones is briefly described as follows. Phenylacrylic acids and phenylpropionic acids were prepared and transformed into acyl chlorides. The phenylacryloyl chlorides and diethyl malonate were condensed to obtain tricarbonyl intermediates, which underwent decarboxylation to provide *β*-keto esters with one phenyl ring, followed by acylation with phenylpropionyl chlorides to obtain tricarbonyl intermediates. Subsequently, decarboxylation of tricarbonyl intermediate provided the diketone intermediate, which underwent selective reduction of the carbonyl group at C-5 to provide *β*’-hydroxy-*α*, *β*-unsaturated ketone intermediate. Finally, the target compounds without phenolic hydroxyl groups were obtained by dehydration, and the target compounds bearing phenolic hydroxyl groups were obtained by dehydration and deprotection.

**Scheme 1:**
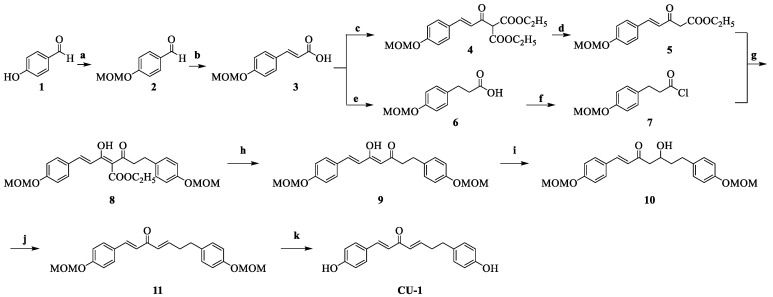
Synthetic route to **CU-1**. Reagents and conditions: (**a**) MOMBr, DIPEA, CH_2_Cl_2_, r.t., 5 h, 92.8%; (**b**) CH_2_(COOH)_2_, pyridine, PhNH_2_, Tol, reflux, 1 h, 94.2%; (**c**) i. N_2_, Oxalyl chloride, CH_2_Cl_2_, r.t., 1 h; ii. CH_2_(COOEt)_2_, NaH, THF, r.t., 2 h, 82.2%; (**d**) H_2_O, DMSO, 110 °C, 5 h, 42.2%; (**e**) H_2_, 10% Pd/C, MeOH, r.t., 2 h, 98.3%; (**f**) N_2_, oxalyl chloride, CH_2_Cl_2_, r.t., 1 h; (**g**) Mg, EtOH, CCl_4_, THF, reflux, 1 h, 52.3%; (**h**) H_2_O, DMSO, 120 °C, 5 h, 54.2%; (**i**) BH_3_·THF, THF, r.t., 1 h, 58.2%; (**j**) cat. *p*-TsOH, EtOAc, 60 °C, 2 h, 86.4%; (**k**) hydrochloric acid, EtOAc, r.t., 1 h, 83.2%.

**CU-1** was taken as an example to illustrate the specific synthesis of the compounds bearing phenolic hydroxyl groups, and the synthetic route is shown in Scheme 1. The starting material 4-hydroxybenzaldehyde **1** was protected via alkylation with bromomethyl methyl ether (MOMBr) to give **2**, which was subsequently transformed into (*E*)-3-(4-(methoxymethoxy)phenyl)acrylic acid **3** by reacting with malonic acid. Intermediate **3** was converted into acyl chloride by reacting with oxalyl chloride and then condensed with diethyl malonate in the presence of NaH to provide **4**, followed by decarboxylation to provide **5**. Both **4** and **5** consisted of ketone-enol tautomeric isomers *via* ^1^H NMR analysis. Catalytic hydrogenation of **3** over Pd/C afforded 3-(4-(methoxymethoxy)phenyl)propanoic acid **6**, which was transformed into the acyl chloride **7** by reacting with oxalyl chloride. Intermediate **5** was condensed with **7** to provide **8**. Next, **8** was decarboxylated to provide (*E*)-1,7-bis(4-(methoxymethoxy)phenyl)-1-heptene-3,5-dione **9**. Using NMR analysis, intermediate **9** was detected as the enol form, which is interconvertible with the keto form. The enol form is more stable due to the presence of a continuous conjugated system and intramolecular hydrogen bonding [20]. Intermediate **9** underwent selective reduction with BH_3_·THF to provide **10**. Intermediate **10** was then dehydrated with the catalyst *p*-toluenesulfonic acid (*p*-TsOH) to afford **11**. Finally, the protective group was removed in the presence of hydrochloric acid to obtain **CU-1**. For the compounds without phenolic hydroxyl groups, the corresponding two steps of introducing the protective groups and removing them in the above route were not required.

In the reaction of converting (*E*)-3-(4-(methoxymethoxy)phenyl)acrylic acid **3** into acyl chloride, the generated hydrogen chloride could readily remove most of the protective MOM groups, resulting in complexity in the reaction. To tackle this problem, we tried several approaches and found that injecting N_2_ into the reaction system was efficient to blow out the generated hydrogen chloride. Using the N_2_ injection method, intermediate **4** was obtained with 82.2% yield. Intermediate acyl chloride **7** was prepared using a similar method.

### 2.2. Biological Evaluation

#### 2.2.1. Cell Growth Inhibition and Anti-Neuroinflammatory Screening of the Target Compounds **CU-1**~**CU-28**

In the present study, we assessed the ability of the target compounds to inhibit NO release in LPS-stimulated BV2 cells. To rule out the possibility that the inhibition of NO release was due to the cytotoxicity of the target compounds, a noncytotoxic dose of the compounds was screened by MTT assay in BV2 cells. As shown in Table 2, most compounds exhibited lower cytotoxicity at 3 μM, while some compounds bearing phenolic hydroxyl groups or multiple methoxy groups on phenyl rings showed higher cytotoxicity. Under the condition of ensuring about 90% cell viability, different concentrations (1 μM, 3 μM) of the target compounds were used in anti-neuroinflammatory activity screening of the corresponding compounds. Based on the screening results, the structure–activity relationships were analyzed and summarized as follows.

(i) Compounds **CU-1**, **CU-2**, **CU-4**, **CU-5**, **CU-7**, **CU-12**, **CU-17**, **CU-18** and **CU-20** with high cytotoxicity showed very weak anti-neuroinflammatory activity (inhibition was 0–16.9%). (ii) Compounds **CU-3**, **CU-14**, **CU-19**, **CU-21** and **CU-23**-bearing methoxy groups exhibited higher anti-neuroinflammatory activity (inhibition was 31.4–66.1%), indicating that the methoxy group might be helpful for anti-neuroinflammatory activity. (iii) Compounds **CU-6**, **CU-8**, **CU-9**, **CU-11**, **CU-13**, **CU-24** and **CU-25**-bearing phenolic hydroxyl groups displayed lower anti-neuroinflammatory activity (inhibition was 0–22.1%), indicating that phenolic hydroxyl group contributed little to anti-neuroinflammatory activity.

#### 2.2.2. Active Compounds Inhibit LPS-Induced NO Release in a Dose-Dependent Manner

**Figure 2:**
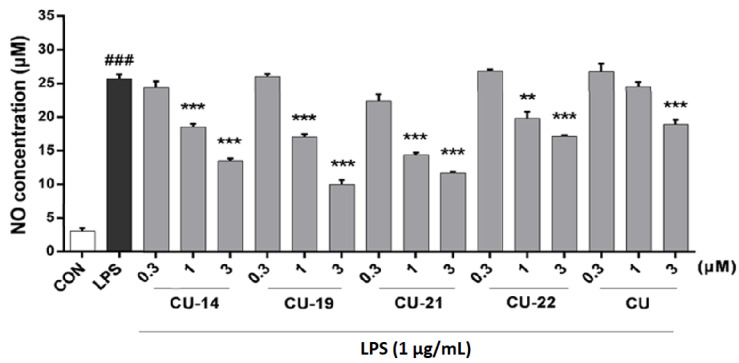
The ability of the active compounds to inhibit LPS-induced NO release in BV2 cells. BV2 cells were pretreated with **CU-14**, **CU-19**, **CU-21**, **CU-22**, and **CU** (0.3, 1.0, 3.0 μM) for 1 h and then stimulated with LPS (1 μg/mL) for 24 h. Results were the mean ± SD, *n* = 3; ^###^ *p* < 0.001 vs. control; ** *p* < 0.01; *** *p* < 0.001 vs. LPS alone.

Since **CU-14**, **CU-19**, **CU-21,** and **CU-22** exhibited higher inhibition of NO release than **CU** at 3 μM, these compounds were chosen for evaluation of dose-dependent inhibitory effects. As shown in Figure 2, all the active compounds showed dose-dependent inhibition. The IC_50_ value of **CU-14** (2.43 μM), **CU-19** (1.60 μM), **CU-21** (1.74 μM), **CU-22** (>3 μM) and **CU** (3 μM) shown in Table 3 is further calculated according to NO inhibition in Figure 2. Accordingly, compounds **CU-19** and **CU-21** with an IC_50_ value of less than 2 μM were selected for further exploration.

#### 2.2.3. The Ability of **CU-19** and **CU-21** to Inhibit LPS-Induced Inflammatory Mediator Release

**Figure 3:**
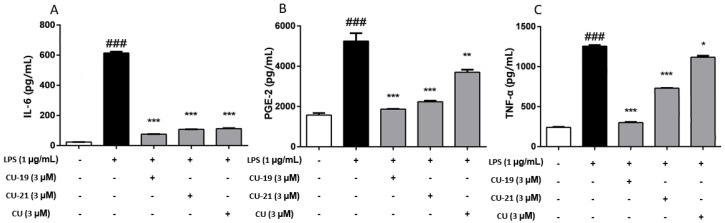
The ability of **CU-19**, **CU-21** and **CU** to inhibit LPS-induced (**A**) IL-6, (**B**) PEG-2, (**C**) TNF-*α* release in BV2 cells. BV2 cells were pretreated with **CU-19**, **CU-21** and **CU** (3 µM) for 1 h and then stimulated with LPS (1 μg/mL) for 24 h. Results were the mean ± SD, *n* = 3; ^###^ *p* < 0.001 vs. control; * *p* < 0.05; ** *p* < 0.01; *** *p* < 0.001 vs. LPS alone.

Interleukin-6 (IL-6), tumor necrosis factor-*α* (TNF-*α*) and prostaglandin E2 (PGE-2) are key inflammatory mediators that drive inflammation progression [21,22]. In order to more fully evaluate the anti-neuroinflammatory properties of **CU-19** and **CU-21**, we assessed their ability to inhibit IL-6, TNF-*α* and PGE-2 release in LPS-induced BV2 cells. As shown in Figure 3, **CU-19** and **CU-21** significantly inhibited IL-6, TNF-*α* and PGE-2 release, and exhibited better inhibitory activity than **CU**.

#### 2.2.4. The Ability of **CU-19** and **CU-21** to Inhibit LPS-Induced iNOS and COX-2 Upregulation

**Figure 4:**
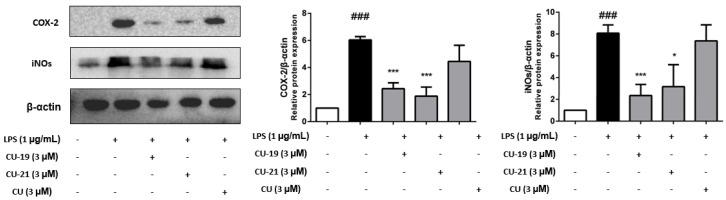
The ability of **CU-19**, **CU-21** and **CU** to inhibit LPS-induced COX-2 and iNOS expression. BV2 cells were pretreated with **CU-19**, **CU-21** and **CU** (3 μM) for 1 h, followed by stimulation with LPS (1 μg/mL) for 24 h. Results were the mean ± SD, *n* = 3; ^###^ *p* < 0.001 vs. control; * *p* < 0.05, *** *p* < 0.001 vs. LPS alone.

Inducible nitric oxide synthase (iNOS) and cyclooxygenase-2 (COX-2) are key regulators and highly expressed during the inflammatory response [23,24]. We therefore evaluated the ability of **CU-19**, **CU-21** and **CU** to modulate LPS-induced iNOS and COX-2 expression in BV2 cells via Western blot. As shown in Figure 4, LPS treatment resulted in a significant increase in the protein levels of COX-2 and iNOS, whereas **CU-19** and **CU-21** significantly inhibited the expression of iNOS and COX-2 and exhibited better inhibitory activity than **CU**.

#### 2.2.5. The Ability of **CU-19** and **CU-21** to Inhibit LPS-Induced NF-κB Activation

**Figure 5:**
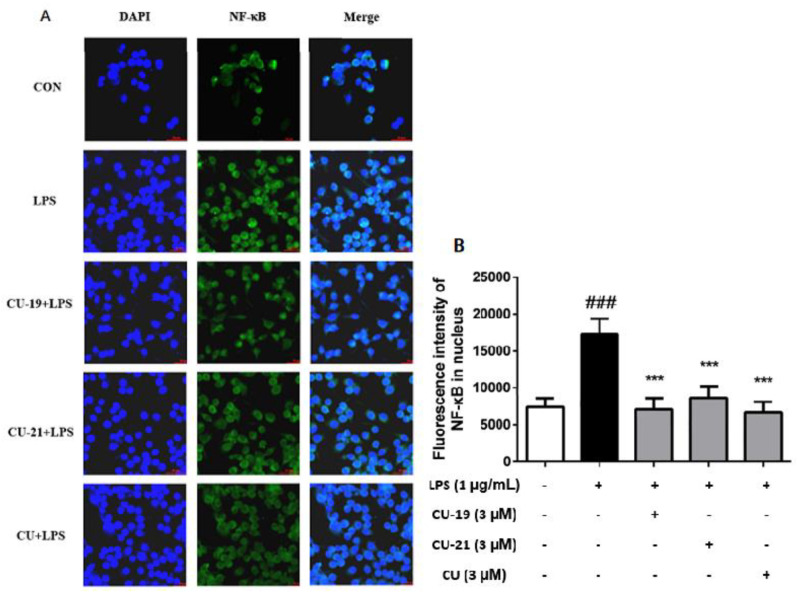
(**A**) The impact of **CU-19** and **CU-21** on LPS-induced NF-*κ*B nuclear translocation. BV2 cells were evaluated following a 24 h treatment with **CU-19**, **CU-21**, **CU** (3 μM) and LPS (1 μg/mL). (**B**) The fluorescence intensity of NF-*κ*B in nucleus. Results were the mean ± SD, *n* = 3; ^###^ *p* < 0.001 vs. control; *** *p* < 0.001 vs. LPS alone.

As nuclear factor-*kappa* B (NF-*κ*B) is an important transcription factor that regulates the expression of most inflammatory mediators, such as iNOS, COX-2, IL-6 and TNF-*α* [25], we examined whether **CU-19** and **CU-21** were able to alter the nuclear translocation of NF-*κ*B using immunofluorescence microscopy. Previous studies showed that **CU** also inhibited NF-*κ*B pathway-mediated inflammation [26]. As shown in Figure 5, **CU-19** and **CU-21** were able to inhibit NF-*κ*B nuclear translocation with a similar efficacy to **CU**.

#### 2.2.6. The Ability of **CU-19** and **CU-21** to Inhibit LPS-Induced MAPK Activation

**Figure 6:**
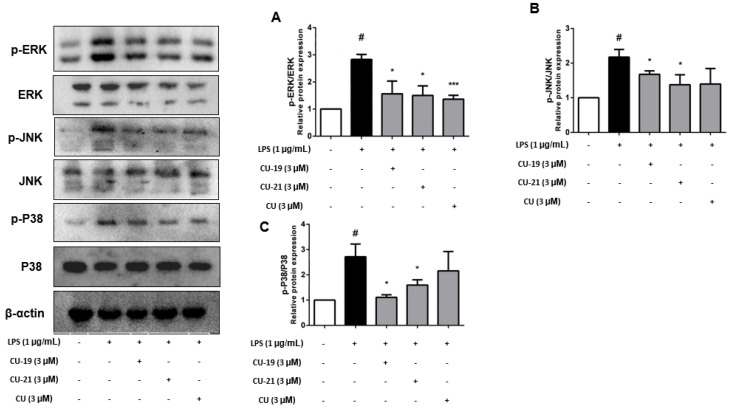
**CU-19**, **CU-21** and **CU** inhibited LPS-induced activation of MAPK signaling pathways in BV2 cells. BV2 cells were pretreated with **CU-19**, **CU-21** and **CU** (3 μM) for 1 h, followed by stimulation with LPS (1 μg/mL) for 24 h, then were subjected to immunoblot analysis using antibodies that specifically recognized phosphorylated and non-phosphorylated p38, JNK, ERK. Results were the mean ± SD, *n* = 3; ^#^ *p* < 0.05 vs. control; * *p* < 0.05, *** *p* < 0.001 vs. LPS alone.

Inflammatory stimuli trigger a signaling cascade mediated by MAPKs, which activates the expression of inflammatory mediators such as COX-2, iNOS, TNF-*α*, IL-1*β* and IL-6 [27,28]. We examined the impact of **CU-19**, **CU-21** and **CU** on the phosphorylation of three MAPKs subtypes, including p38, c-Jun NH_2_-terminal kinase (JNK) and extracellular regulated protein kinases (ERK). As shown in Figure 6, **CU-19** and **CU-21** significantly decreased JNK, ERK and p38 phosphorylation compared to the LPS group.

### 2.3. Pharmacokinetic Study

**Figure 7:**
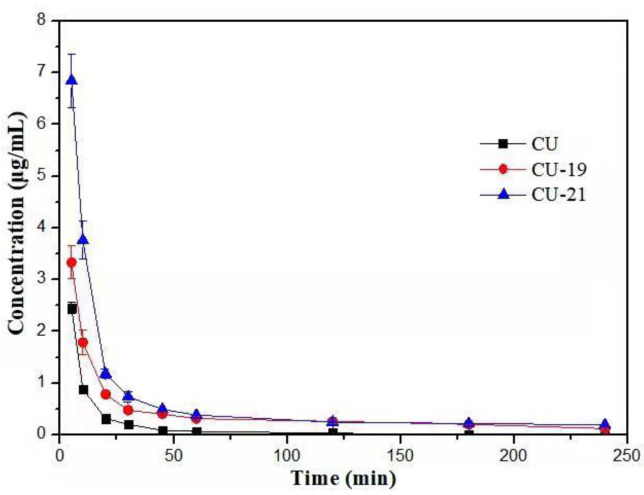
Mean (±SD) plasma concentration–time profiles of **CU-19, CU-21** and **CU** in the plasma of healthy rats (*n* = 4), which were administered a single intravenous dose of 20 mg compound per kg of body weight.

In order to investigate the metabolic stability of the active compounds, a preliminary pharmacokinetic study was conducted in rats. After a single intravenous dose of 20 mg/kg, 0.5 mL of blood was obtained from the fossa orbitalis veniplex of rats at 5, 10, 20, 30, 45, 60, 120, 180 and 240 min. HPLC was used to determine the concentration of each compound in plasma and the DAS software was used for the noncompartmental pharmacokinetic analysis of the plasma concentration–time data. The representative HPLC spectrums are shown in Figures S1–S9 (Supplementary Materials). A metabolite of **CU** was detected (Figures S1–S3), and no obvious metabolites of **CU-19** and **CU-21** were detected (Figures S4–S9). The mean (±SD) plasma concentration–time curves of **CU**, **CU-19** and **CU-21** in the plasma of healthy rats (*n* = 4) are shown in Figure 7. The pharmacokinetic parameters (C_max_, the peak concentration; T_1/2_, the half-life; CL, the total body clearance; AUC, the area under the curve; MRT, the mean residence time) are presented in Table 4. The C_max_ of **CU** was lower than that of **CU-19** and **CU-21**. Additionally, the differences in the AUC also exhibited a large increase from **CU** to **CU-19** and **CU-21**. Furthermore, the T_1/2_ of **CU-19** and **CU-21** increased 4.5- and 5.5-fold in comparison with that of **CU**, respectively. In summary, the results of the preliminary pharmacokinetic study indicate, to some extent, the deletion of *β*-diketone decreases the degree and speed of metabolism of curcuminoids, and the mono-carbonyl analogues of **CU** may possess much better pharmacokinetic profiles than **CU**.

## 3. Conclusions

In summary, we developed a preparation method of 1,7-diphenyl-1,4-heptadien-3-ones, synthesized 28 compounds and evaluated anti-neuroinflammatory effects of the target compounds. Pharmacological results showed that some analogues bearing the methoxy group effectively inhibited the release of LPS-induced NO in BV2 cells. Compounds **CU-19** and **CU-21,** with potent inhibition of NO release, reduced the level of TNF-*α*, IL-6 and PGE-2, downregulated the expression of COX-2 and iNOS in LPS-induced BV2 cells. A study on the mechanism of action revealed that **CU-19** and **CU-21** inhibited the nuclear translocation of NF-*κ*B and the phosphorylation of MAPKs (ERK, JNK and p38). A preliminary pharmacokinetic study showed that the pharmacokinetic properties of **CU-19** and **CU-21** were significantly ameliorated. The research results indicate that **CU-19** and **CU-21** are promising anti-neuroinflammatory agents and are worthy of further study.

## 4. Experimental Section

### 4.1. Chemistry and HPLC Analysis

All of the starting materials, reagents and solvents are commercially available and were used without further purification. Melting points were determined with a Yanaco MP-52581 apparatus, which were uncorrected. All column chromatography was carried out on silica gel (200~300 mesh). The ^1^H NMR and ^13^C NMR spectra were recorded on a Bruker instrument (Bruker AVANCE Ⅲ) shield spectrometer at 600 and 151 MHz, respectively. High-resolution mass spectra (HRMS) were recorded by an Agilent Accurate-Mass QTOF 6530 (Agilent, Santa Clara, CA, USA) instrument in ESI mode. The reactions were monitored by thin-layer chromatography (TLC; HG/T2354-92, GF254).

The purities were determined by high performance liquid chromatography (HPLC). Purities of all final compounds were more than 97% (Shimadzu, LC-10AT). The column was ODS-C18 (4.6 × 250 mm, 5 µm). Chromatography conditions for compounds **CU-3**, **CU-4**, **CU-10**, **CU-14**, **CU-15**, **CU-16**, **CU-21**, **CU-22**, **CU-23**, **CU-28**, mobile phase: MeOH:H_2_O = 80:20; **CU-5**, **CU-6**, **CU-24**, **CU-25**, mobile phase: MeOH:H_2_O = 75:25; **CU-7**, **CU-8**, **CU-9**, **CU-11**, **CU-27**, mobile phase: MeOH:H_2_O = 70:30; **CU-1**, **CU-2**, **CU-12**, **CU-13**, **CU-17**, **CU-18**, **CU-19**, **CU-20**, **CU-26**, mobile phase: MeOH:H_2_O = 55:45; wavelength: 254 nm; column temperature: 30 °C; flow rate: 1.0 mL/min.

#### 4.1.1. The Synthesis of 4-(methoxymethoxy)benzaldehyde **2**

Diisopropylethyl amine (DIPEA, 20.4 mL, 122.8 mmol) was added to a stirred solution of 4-hydroxybenzaldehyde (10.00 g, 81.9 mmol) in CH_2_Cl_2_ (50 mL) cooled in an ice-water bath. Then, MOMBr (6.7 mL, 81.9 mmol) was added dropwise. After the addition was complete, the mixture was stirred at room temperature for 5 h. The reaction was quenched by adding water (50 mL). The organic phase was separated and the aqueous phase was extracted with CH_2_Cl_2_ (3 × 50 mL). The combined organic phase was washed successively with 1 M hydrochloric acid, water, 1 M aqueous sodium hydroxide and brine, and then dried over anhydrous sodium sulfate., The filtrate was concentrated under reduced pressure to afford intermediate **2** (18.90 g, 92.8%) as colorless oil. The product was used directly for the next step without further purification. ^1^H NMR (600 MHz, DMSO-*d*_6_) δ 9.89 (s, 1H, -CHO), 7.88 (d, *J* = 8.7 Hz, 2H, Ar-H), 7.21 (d, *J* = 8.7 Hz, 2H, Ar-H), 5.32 (s, 2H, -OCH_2_O-), 3.40 (s, 3H, -OCH_3_). ESI-HRMS (m/z): calcd. for C_9_H_11_O_3_ [M+H]^+^ 167.0708, found167.0707.

#### 4.1.2. The Synthesis of (*E*)-3-(4-(methoxymethoxy)phenyl)acrylic Acid **3**

Malonic acid (11.30 g, 107.5 mmol), pyridine (15.7 mL, 194.8 mmol), and aniline (0.7 mL, 7.2 mmol) were added to a stirred solution of substituted benzaldehyde **2** (12.00 g, 72.2 mmol) in toluene (20 mL). The resulting mixture was refluxed for 1 h, then the solvent was evaporated under reduced pressure. The residue was poured into 1M hydrochloric acid solution. The precipitate was collected by filtration to provide **3** (14.20 g, 94.2%) as a white solid. The product was of high purity and was used for the next step without further purification. m.p.: 145–147 °C. ^1^H NMR (600 MHz, DMSO-*d*_6_) δ 12.25 (s, 1H, -COOH), 7.64 (d, *J* = 8.7 Hz, 2H, Ar-H), 7.54 (d, *J* = 16.0 Hz, 1H, -CH=), 7.05 (d, *J* = 8.7 Hz, 2H, Ar-H), 6.39 (d, *J* = 16.0 Hz, 1H, -CH=), 5.24 (s, 2H, -OCH_2_O-), 3.38 (s, 3H, -OCH_3_). ESI-HRMS (m/z): calcd. for C_11_H_11_O_4_ [M-H]^−^ 207.0657, found 207.0659.

#### 4.1.3. The Synthesis of Diethyl(*E*)-2-(3-(4-(methoxymethoxy)phenyl)acryloyl)malonate **4** and Diethyl(*E*)-2-(1-hydroxy-3-(4-(methoxymethoxy)phenyl)allylidene)malonate **4′**

A suspension of substituted phenyl acrylic acid **3** (10.00 g, 48.0 mmol) in CH_2_Cl_2_ (300 mL) was cooled to 0 °C and nitrogen was injected continuously into the suspension. Oxalyl chloride (8.1 mL, 96.1 mmol) was added dropwise with stirring. After the addition was complete, the mixture was stirred for 1 h at room temperature. Then, the solvent was evaporated under reduced pressure and the residue was azeotroped twice with CH_2_Cl_2_ to afford acyl chloride. Sodium hydride (60% dispersion in oil, 3.80 g, 96.1 mmol) was added in four batches to a stirred solution of diethyl malonate (14.6 mL, 96.1 mmol) in anhydrous tetrahydrofuran (THF, 70 mL) in 30 min under nitrogen. Then, the solution of acyl chloride prepared above in anhydrous THF (20 mL) was added dropwise and the mixture was stirred for 2 h at room temperature. The reaction was quenched by the careful addition of water (50 mL). The solvent was evaporated under reduced pressure. The residue was adjusted to pH 5 with 1 M hydrochloric acid and then extracted with EtOAc (3 × 50 mL). The combined organic phase was washed with brine and dried over anhydrous sodium sulfate. The filtrate was concentrated under reduced pressure to afford a mixture of intermediate **4** and **4′** (13.80 g, 82.2%) as yellow oil. The product was used directly for the next step without further purification. **4**, ^1^H NMR (600 MHz, DMSO-*d*_6_) δ 7.69 (d, *J* = 8.7 Hz, 2H, Ar-H), 7.65 (d, *J* = 16.1 Hz, 1H, -CH=), 7.09 (d, *J* = 8.6 Hz, 2H, Ar-H), 6.89 (d, *J* = 16.1 Hz, 1H, -CH=), 5.39 (s, 1H, -CH-), 5.26 (s, 2H, -OCH_2_O-), 4.18 (q, *J* = 7.0 Hz, 4H, -OCH_2_CH_3_ × 2), 3.38 (s, 3H, -OCH_3_), 1.19 (t, *J* = 7.1 Hz, 6H, -OCH_2_CH_3_×2). **4′**, ^1^H NMR (600 MHz, DMSO-d_6_) δ 12.93 (s, 1H, -OH), 7.61 (d, *J* = 8.7 Hz, 2H, Ar-H), 7.57 (d, *J* = 15.7 Hz, 1H, -CH=), 7.08 (d, *J* = 8.6 Hz, 2H, Ar-H), 6.88 (d, *J* = 15.7 Hz, 2H, -CH=), 5.25 (s, 2H, -OCH_2_O-), 4.26 (q, *J* = 7.1 Hz, 4H, -OCH_2_CH_3_ × 2), 3.38 (s, 3H, -OCH_3_), 1.26 (t, *J* = 7.1 Hz, 6H, -OCH_2_CH_3_ × 2). ESI-HRMS (m/z): calcd. for C_18_H_22_O_7_Na [M+Na]^+^ 373.1263, found 373.1280.


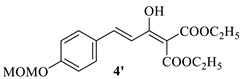


#### 4.1.4. The Synthesis of Ethyl (E)-5-(4-(methoxymethoxy)phenyl)-3-oxo-4-pentenoate **5** and Ethyl (2Z,4E)-3-hydroxy-5-(4-(methoxymethoxy)phenyl)penta-2,4-dienoate **5′**

A mixture of **4** and **4′** (12.00 g, 34.2 mmol), water (1.2 mL, 68.4 mmol) and DMSO (20 mL) was heated at 110 °C for 5 h and then quenched with water (50 mL) and extracted with EtOAc (3×50 mL). The combined organic phase was washed with brine and dried over anhydrous sodium sulfate. The filtrate was concentrated under reduced pressure. The residue was separated by silica gel chromatography with petroleum ether/ethyl acetate (8:1) as an eluting agent to afford a mixture of **5** and **5′** (9.50 g. 42.2%) as yellow oil. **5**, ^1^H NMR (600 MHz, DMSO-*d*_6_) δ 7.68 (d, *J* = 8.7 Hz, 2H, Ar-H), 7.63 (d, *J* = 16.3 Hz, 1H, -CH=), 7.08 (d, *J* = 8.7 Hz, 2H, Ar-H), 6.78 (d, *J* = 16.2 Hz, 1H, -CH=), 5.25 (s, 2H, -OCH_2_O-), 4.11 (q, *J* = 7.1 Hz, 2H, -OCH_2_CH_3_), 3.83 (s, 2H, -CH_2_-), 3.38 (s, 3H, -OCH_3_), 1.19 (t, *J* = 7.1 Hz, 3H, -OCH_2_CH_3_). **5′**, ^1^H NMR (600 MHz, DMSO-*d*_6_) δ 11.94 (s, 1H, -OH), 7.59 (d, *J* = 8.7 Hz, 2H, Ar-H), 7.33 (d, *J* = 16.0 Hz, 1H, -CH=), 7.05 (d, *J* = 8.7 Hz, 2H, Ar-H), 6.68 (d, *J* = 16.0 Hz, 1H, -CH=), 5.33 (s, 1H, -CH=), 5.23 (s, 2H, -OCH_2_O-), 4.19 (q, *J* = 7.1 Hz, 2H, -OCH_2_CH_3_), 3.38 (s, 3H, -OCH_3_), 1.24 (t, *J* = 7.1 Hz, 3H, -OCH_2_CH_3_). ESI-HRMS (m/z): calcd. for C_15_H_19_O_5_ [M+H]^+^ 279.1232, found 279.1250.


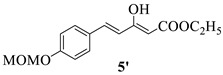


#### 4.1.5. The Synthesis of 3-(4-(methoxymethoxy)phenyl)propionic acid **6**

Pd/C (l0%, 0.30 g) was added to a stirred solution of **3** (10.00 g, 48.0 mmol) in methanol (40 mL), and the reaction mixture was stirred under hydrogen atmosphere for 2 h at room temperature. The catalyst was then filtered off and the solvent was evaporated under reduced pressure to furnish the substituted propionic acid **6** (9.90 g, 98.3%) as a white solid. The product was of high purity and was used in the next step without further purification. m.p.: 50–52 °C. ^1^H NMR (600 MHz, DMSO-*d*_6_) δ 12.08 (s, 1H, -COOH), 7.14 (d, *J* = 8.5 Hz, 2H, Ar-H), 6.92 (d, *J* = 8.5 Hz, 2H, Ar-H), 5.14 (s, 2H, -OCH2O-), 3.36 (s, 3H, -OCH3), 2.75 (t, *J* = 7.6 Hz, 2H, -CH2), 2.48 (t, *J* = 7.6 Hz, 2H, -CH2). ESI-HRMS (m/z): calcd. for C_11_H_13_O_4_ [M-H]^−^ 209.0841, found 209.0829.

#### 4.1.6. The Synthesis of (1*E*,3*E*)-1,7-bis(4-(methoxymethoxy)phenyl)-3-hydroxy-4-(ethoxycarbonyl)-1,3-heptadiene-5-one **8**

A suspension of substituted propionic acid **6** (8.20 g, 39.0 mmol) in CH_2_Cl_2_ (240 mL) was cooled to 0 °C, and nitrogen was injected continuously into the reaction mixture. Oxalyl chloride (6.6 mL, 78.0 mmol) was added with stirring and the solution was stirred for 1.5 h. The solvent was evaporated under reduced pressure and the residue was azeotroped twice with CH_2_Cl_2_ to afford acyl chloride **7**. Magnesium turnings (0.70 g, 31.2 mmol) were added to a solution of absolute ethanol (1.5 mL, 26.0 mmol) and carbon tetrachloride (0.2 mL, 2.6 mmol), and the mixture was stirred for 5 min at room temperature. Then, THF (10 mL) was added and the mixture was heated to 60 °C. A solution of **5** (7.20 g, 26.0 mmol) in THF (10 mL) was added, and the mixture was refluxed for 1 h. Then, a solution of **7** prepared above in THF (5 mL) was added dropwise. After the addition was complete, the mixture was refluxed for 1 h. The reaction was cooled and quenched by careful addition of water (50 mL). After the removal of the solvent under reduced pressure, the residue was adjusted to pH 6 with 1 M hydrochloric acid, then extracted with EtOAc (3×50 mL). The combined organic phase was washed with brine and dried over anhydrous sodium sulfate. The filtrate was concentrated under reduced pressure and the residue was separated using silica gel chromatography with petroleum ether/ethyl acetate (5:1) as an eluting agent to furnish **8** (6.40 g. 52.3%) as yellow oil. ^1^H NMR (600 MHz, DMSO-*d*_6_) δ 7.75 (d, *J* = 15.7 Hz, 1H, -CH=), 7.66 (d, *J* = 8.8 Hz, 2H, Ar-H), 7.14 (d, *J* = 8.5 Hz, 2H, Ar-H), 7.09 (d, *J* = 8.8 Hz, 2H, Ar-H), 7.05 (d, *J* = 15.6 Hz, 1H, -CH=), 6.93 (d, *J* = 8.6 Hz, 2H, Ar-H), 5.26 (s, 2H, -OCH_2_O-), 5.14 (s, 2H, -OCH_2_O-), 4.29 (q, *J* = 7.1 Hz, 2H, -OCH_2_CH_3_), 3.38 (s, 3H, -OCH_3_), 3.36 (s, 3H, -OCH_3_), 2.96 (t, *J* = 7.7 Hz, 2H, -CH_2_), 2.84 (t, *J* = 7.6 Hz, 2H, -CH_2_), 1.28 (t, *J* = 7.1 Hz, 3H, -OCH_2_CH_3_). ESI-HRMS (m/z): calcd. for C_26_H_30_O_8_Na [M+Na]^+^ 493.1838, found 493.1824.

#### 4.1.7. The Synthesis of (1*E*,3*E*)-1,7-bis(4-(methoxymethoxy)phenyl)-3-hydroxy-1,3-heptadiene-5-one **9**

A mixture of **8** (3.10 g, 6.4 mmol), water (0.2 mL, 12.8 mmol) and DMSO (15 mL) was heated at 120 °C for 5 h, then quenched with water (30 mL) and extracted with EtOAc (3 × 30 mL). The combined organic phase was washed with brine and dried over anhydrous sodium sulfate. The filtrate was concentrated under reduced pressure and the residue was separated by silica gel chromatography with petroleum ether/ethyl acetate (5:1) as an eluting agent to furnish **9** (1.40 g, 54.2%) as yellow oil. ^1^H NMR (600 MHz, DMSO-*d*_6_) δ 15.51 (s, 1H, -OH), 7.64 (d, *J* = 8.8 Hz, 2H, Ar-H), 7.52 (d, *J* = 15.9 Hz, 1H, -CH=), 7.16 (d, *J* = 8.6 Hz, 2H, Ar-H), 7.06 (d, *J* = 8.8 Hz, 2H, Ar-H), 6.93 (d, *J* = 8.6 Hz, 2H, Ar-H), 6.67 (d, *J* = 15.9 Hz, 1H, -CH=), 5.91 (s, 1H, -CH=), 5.24 (s, 2H, -OCH_2_O-), 5.14 (s, 2H, -OCH_2_O-), 3.38 (s, 3H, -OCH_3_), 3.35 (s, 3H, -OCH_3_), 2.84 (t, *J* = 7.4 Hz, 2H, -CH_2_), 2.70 (t, *J* = 7.6 Hz, 2H, -CH_2_). ESI-HRMS (m/z): calcd. for C_23_H_26_O_6_Na [M+Na]^+^ 421.1627, found 421.1614.

#### 4.1.8. The Synthesis of (*E*)-1,7-bis(4-(methoxymethoxy)phenyl)-5-hydroxy-1-heptene-3-one **10**

An amount of 1 M BH_3_·THF (2.5 mL, 2.5 mmol) was added dropwise to a stirred solution of **9** (1.10 g, 2.5 mmol) in anhydrous THF (40 mL) at 0 °C under nitrogen atmosphere. After the addition was complete, the reaction mixture was stirred for 1 h at room temperature and then quenched by careful addition of 1 M sodium hydroxide and concentrated under reduced pressure. The residue was adjusted to pH 6.0 with 1 M hydrochloric acid and then extracted with EtOAc (3 × 20 mL). The combined organic phase was washed with brine and dried over anhydrous sodium sulfate. The filtrate was concentrated under reduced pressure and the residue was separated by silica gel chromatography with petroleum ether/ethyl acetate (4:1) as an eluting agent to furnish **10** (0.60 g, 58.2%) as a pale yellow solid, m.p.: 45–47 °C. ^1^H NMR (600 MHz, DMSO-*d*_6_) δ 7.66 (d, *J* = 8.4 Hz, 2H, Ar-H), 7.54 (d, *J* = 16.2 Hz, 1H, -CH=), 7.11 (d, *J* = 8.1 Hz, 2H, Ar-H), 7.07 (d, *J* = 8.4 Hz, 2H, Ar-H), 6.92 (d, *J* = 8.1 Hz, 2H, Ar-H), 6.77 (d, *J* = 16.2 Hz, 1H, -CH=), 5.24 (s, 2H, -OCH_2_O-), 5.13 (s, 2H, -OCH_2_O-), 4.70 (d, *J* = 5.6 Hz, 1H, -OH), 4.01–3.92 (m, 1H, -CH-), 3.38 (s, 3H, -OCH_3_), 3.36 (s, 3H, -OCH_3_), 2.81 (dd, *J* = 14.9, 7.9 Hz, 1H, -CH_2_), 2.73–2.62 (m, 2H, -CH_2_), 2.59–2.53 (m, 1H, -CH_2_), 1.72–1.58 (m, 2H, -CH_2_). ESI-HRMS (m/z): calcd. for C_23_H_28_O_6_Na [M+Na]^+^ 423.1784, found 423.1835.

#### 4.1.9. The Synthesis of (1*E*,4*E*)-1,7-bis(4-(methoxymethoxy)phenyl)-1,4-heptadiene-3-one **11**

*p*-TsOH (0.02 g, 0.1 mmol) was added to a stirred solution of **10** (0.50 g, 1.3 mmol) in dry EtOAc (20 mL). The reaction was stirred for 2 h under nitrogen atmosphere at 60 °C and then cooled to room temperature. The organic phase was separated and washed successively with saturated aqueous sodium bicarbonate, water and brine and dried over anhydrous sodium sulfate. The filtrate was concentrated under reduced pressure and the residue was separated by silica gel chromatography with petroleum ether/ethyl acetate (5:1) as an eluting agent to furnish 11 (0.40 g, 86.4%) as yellow oil. ^1^H NMR (600 MHz, CDCl_3_) δ 7.58 (d, *J* = 15.9 Hz, 1H, -CH=), 7.51 (d, *J* = 8.6 Hz, 2H, Ar-H), 7.12 (d, *J* = 8.4 Hz, 2H, Ar-H), 7.05 (d, *J* = 8.5 Hz, 2H, Ar-H), 7.03–6.95 (m, 3H, Ar-H and -CH=), 6.83 (d, *J* = 15.9 Hz, 1H, -CH=), 6.44 (d, *J* = 15.7 Hz, 1H, -CH=), 5.21 (s, 2H, -OCH2O-), 5.15 (s, 2H, -OCH2O-), 3.48 (s, 3H, -OCH3), 3.48 (s, 3H, -OCH3), 2.78 (t, *J* = 7.7 Hz, 2H, -CH2), 2.60–2.54 (m, 2H, -CH2). ESI-HRMS (m/z): calcd. for C_23_H_26_O_5_Na [M+Na]+ 405.1678, found 405.1718.

#### 4.1.10. The Synthesis of the Target Compounds

Concentrated hydrochloric acid (0.2 mL, 2.5 mmol) was added to a solution of **11** (0.20 g, 0.5 mmol) in EtOAc (20 mL) and the reaction mixture was stirred for 1 h at room temperature. The reaction solution was adjusted to pH 6 with 1M aqueous sodium hydroxide and then extracted with EtOAc (3 × 20 mL). The combined organic phase was washed with brine and dried over anhydrous sodium sulfate. The filtrate was concentrated under reduced pressure and the residue was separated by silica gel chromatography with petroleum ether/ethyl acetate (5:1) as an eluting agent to furnish **CU-1** (0.10 g, 83.2%) as a yellow solid.

All the target compounds were synthesized using a similar method to the one described for the synthesis of **CU-1**. For the compounds without phenolic hydroxyl groups, such as **CU-2,** the corresponding steps of introducing the protective groups and removing them in the above route were not required.

##### (1*E*,4*E*)-1,7-bis(4-hydroxyphenyl)-1,4-heptadien-3-one (**CU-1**)

Yellow solid; yield: 83.2%; m.p.: 154–156 °C. ^1^H NMR (600 MHz, DMSO-*d*_6_) δ 10.04 (s, 1H, Ar-OH), 9.16 (s, 1H, Ar-OH), 7.60 (d, *J* = 8.6 Hz, 2H, Ar-H), 7.55 (d, *J* = 16.0 Hz, 1H, -CH=), 7.03 (d, *J* = 8.4 Hz, 2H, Ar-H), 7.00–6.95 (m, 2H, -CH= and -CH=), 6.82 (d, *J* = 8.6 Hz, 2H, Ar-H), 6.68 (d, *J* = 8.4 Hz, 2H, Ar-H), 6.51 (d, *J* = 15.7 Hz, 1H, -CH=), 2.68 (t, *J* = 7.7 Hz, 2H, -CH_2_), 2.53–2.49 (m, 2H, -CH_2_). ^13^C NMR (151 MHz, DMSO-*d*_6_) δ 188.5, 160.2, 155.8, 146.8, 143.2, 131.4, 130.9, 129.8, 129.5, 126.0, 122.1, 116.1, 115.4, 34.4, 33.3. ESI-HRMS (m/z): calcd. for C_19_H_18_O_3_[M-H]^−^ 293.1178, found 293.1183. Purity: 99.0% (t_R_ = 7.00 min).

##### (1*E*,4*E*)-1,7-bis(3,4-dimethoxyphenyl)-1,4-heptadien-3-one (**CU-2**)

Yellow solid; yield: 51.7%; m.p.: 97–99 °C. ^1^H NMR (600 MHz, CDCl_3_) δ 7.57 (d, *J* = 15.9 Hz, 1H, -CH=), 7.15 (d, *J* = 8.3 Hz, 1H, Ar-H), 7.09 (s, 1H, Ar-H), 7.02 (dt, *J* = 14.6, 6.8 Hz, 1H, -CH=), 6.88 (d, *J* = 8.3 Hz, 1H, Ar-H), 6.82–6.79 (m, 2H, Ar-H and -CH=), 6.75–6.72 (m, 2H, Ar-H), 6.47 (d, *J* = 15.5 Hz, 1H, -CH=), 3.93 (s, 6H, Ar-OCH_3_), 3.88 (s, 3H, Ar-OCH_3_), 3.86 (s, 3H, Ar-OCH_3_), 2.78 (t, *J* = 7.7 Hz, 2H, -CH_2_), 2.61–2.57 (m, 2H, -CH_2_). ^13^C NMR (151 MHz, CDCl_3_) δ 188.8, 151.2, 149.1, 148.8, 147.3, 146.3, 143.1, 133.4, 129.4, 127.6, 123.0, 120.1, 111.7, 111.2, 111.0, 109.7, 55.8, 55.8, 55.8, 55.7, 34.5, 34.0. ESI-HRMS (m/z): calcd. for C_23_H_26_O_5_Na [M+Na]^+^ 405.1678, found 405.1693. Purity: 98.2% (t_R_ = 14.87 min).

##### (1*E*,4*E*)-7-phenyl-1-(4-methoxyphenyl)-1,4-heptadien-3-one (**CU-3**)

Pale yellow solid; yield: 51.4%; m.p.: 48–50 °C. ^1^H NMR (600 MHz, CDCl_3_) δ 7.59 (d, *J* = 15.9 Hz, 1H, -CH=), 7.52 (d, *J* = 8.7 Hz, 2H, Ar-H), 7.31 (t, *J* = 7.6 Hz, 2H, Ar-H), 7.24–7.18 (m, 3H, Ar-H), 7.01 (dt, *J* = 15.6, 6.8 Hz, 1H, -CH=), 6.92 (d, *J* = 8.7 Hz, 2H, Ar-H), 6.82 (d, *J* = 15.9 Hz, 1H, -CH=), 6.44 (d, *J* = 15.6 Hz, 1H, -CH=), 3.85 (s, 3H, Ar-OCH_3_), 2.83 (t, *J* = 7.8 Hz, 2H, -CH_2_), 2.68–2.51 (m, 2H, -CH_2_). ^13^C NMR (151 MHz, CDCl_3_) δ 189.1, 161.5, 146.2, 143.0, 140.9, 130.0, 129.7, 128.5, 128.4, 127.5, 126.1, 122.7, 114.4, 55.4, 34.5, 34.4. ESI-HRMS (m/z): calcd. for C_20_H_20_O_2_Na [M+Na]^+^ 315.1361, found 315.1354. Purity: 95.7% (t_R_ = 11.00 min).

##### (1*E*,4*E*)-7-phenyl-1-(3,4,5-trimethoxyphenyl)-1,4-heptadien-3-one (**CU-4**)

Yellow solid; yield: 50.9%; m.p.: 95–97 °C. ^1^H NMR (600 MHz, CDCl_3_) δ 7.53 (d, *J* = 15.8 Hz, 1H, -CH=), 7.31 (t, *J* = 7.6 Hz, 2H, Ar-H), 7.23-7.20 (m, 3H, Ar-H), 7.03 (dt, *J* = 15.6, 6.8 Hz, 1H, -CH=), 6.83 (d, *J* = 15.9 Hz, 1H, -CH=), 6.79 (s, 2H, Ar-H), 6.47 (d, *J* = 15.6 Hz, 1H, -CH=), 3.90 (s, 6H, Ar-OCH_3_), 3.89 (s, 3H, Ar-OCH_3_), 2.84 (t, *J* = 7.8 Hz, 2H, -CH_2_), 2.64–2.58 (m, 2H, -CH_2_). ^13^C NMR (151 MHz, CDCl_3_) δ 188.9, 153.4, 146.7, 143.2, 140.8, 140.2, 130.2, 129.4, 128.5, 128.3, 126.2, 124.4, 105.4, 60.9, 56.1, 34.5, 34.3. ESI-HRMS (m/z): calcd. for C_22_H_24_O_4_Na [M+Na]^+^ 375.1572, found 375.1567. Purity: 98.1% (t_R_ = 8.15 min).

##### (1*E*,4*E*)-1-(2-hydroxyphenyl)-7-phenyl-1,4-heptadien-3-one (**CU-5**)

Earth-yellow solid; yield: 82.1%; m.p.: 134–136 °C. ^1^H NMR (600 MHz, DMSO-*d*_6_) δ 10.21 (s, 1H, Ar-OH), 7.84 (d, *J* = 16.1 Hz, 1H, -CH=), 7.67 (d, *J* = 7.7 Hz, 1H, Ar-H), 7.30 (t, *J* = 7.5 Hz, 2H, Ar-H), 7.27–7.23 (m, 3H, Ar-H), 7.21–7.17 (m, 2H, Ar-H and -CH=), 7.00 (dt, *J* = 15.7, 6.8 Hz, 1H, -CH=), 6.91 (d, *J* = 8.1 Hz, 1H, Ar-H), 6.85 (t, *J* = 7.5 Hz, 1H, Ar-H), 6.49 (d, *J* = 15.7 Hz, 1H, -CH=), 2.80 (t, *J* = 7.7 Hz, 2H, -CH_2_), 2.61–2.55 (m, 2H, -CH_2_). ^13^C NMR (151 MHz, CDCl_3_) δ 191.3, 156.4, 147.5, 140.8, 140.5, 131.9, 129.3, 129.1, 128.5, 128.4, 126.2, 125.3, 121.9, 120.4, 116.7, 34.5, 34.5. ESI-HRMS (m/z): calcd. for C_19_H_19_O_2_[M+H]^+^ 279.1385, found 279.1390. Purity: 98.5% (t_R_ = 11.37 min).

##### (1*E*,4*E*)-1-(3-hydroxyphenyl)-7-phenyl-1,4-heptadien-3-one (**CU-6**)

Yellow solid; yield: 83.3%; m.p.: 94–96 °C. ^1^H NMR (600 MHz, DMSO-*d*_6_) δ 9.62 (s, 1H, Ar-OH), 7.53 (d, *J* = 15.9 Hz, 1H, -CH=), 7.30 (t, *J* = 7.5 Hz, 2H, Ar-H), 7.27–7.22 (m, 3H, Ar-H), 7.21 -7.16 (m, 2H, Ar-H), 7.13–7.08 (m, 2H, Ar-H and -CH=), 7.05 (dt, *J* = 15.4, 6.8 Hz, 1H, -CH=), 6.85 (d, *J* = 7.8 Hz, 1H, Ar-H), 6.55 (d, *J* = 15.7 Hz, 1H, -CH=), 2.81 (t, *J* = 7.7 Hz, 2H, -CH_2_), 2.62–2.55 (m, 2H, -CH_2_). ^13^C NMR (151 MHz, CDCl_3_) δ 190.0, 156.5, 147.8, 143.8, 140.7, 136.0, 130.1, 129.4, 128.5, 128.3, 126.2, 124.8, 120.7, 118.0, 115.0, 34.4, 34.4. ESI-HRMS (m/z): calcd. for C_19_H_19_O_2_[M+H]^+^ 279.1385, found 279.1399. Purity: 98.6% (t_R_ = 9.59 min).

##### (1*E*,4*E*)-1-(4-hydroxyphenyl)-7-phenyl-1,4-heptadien-3-one (**CU-7**)

Grayish yellow solid; yield: 80.2%; m.p.: 121–123 °C. ^1^H NMR (600 MHz, CDCl_3_) δ 7.58 (d, *J* = 15.9 Hz, 1H, -CH=), 7.48 (d, *J* = 8.6 Hz, 2H, Ar-H), 7.30 (t, *J* = 7.6 Hz, 2H, Ar-H), 7.21 (m, 3H, Ar-H), 7.02 (dt, *J* = 15.4, 6.8 Hz, 1H, -CH=), 6.86 (d, *J* = 8.5 Hz, 2H, Ar-H), 6.82 (d, *J* = 15.9 Hz, 1H, -CH=), 6.44 (d, *J* = 15.7 Hz, 1H, -CH=), 5.32 (s, 1H, Ar-OH), 2.83 (t, *J* = 7.8 Hz, 2H, -CH_2_), 2.63–2.58 (m, 2H, -CH_2_). ^13^C NMR (151 MHz, CDCl_3_) δ 190.1, 158.7, 147.2, 144.1, 140.7, 130.4, 129.5, 128.5, 128.3, 127.0, 126.2, 122.2, 116.1, 34.4, 34.4. ESI-HRMS (m/z): calcd. for C_19_H_17_O_2_ [M-H]^−^ 277.1229, found 277.1247. Purity: 99.0% (t_R_ = 15.09 min).

##### (1*E*,4*E*)-1-(3-hydroxy-4-methoxyphenyl)-7-phenyl-1,4-heptadien-3-one (**CU-8**)

Yellow solid; yield: 81.6%; m.p.: 72–74 °C. ^1^H NMR (600 MHz, DMSO-*d*_6_) δ 9.16 (s, 1H, Ar-OH), 7.50 (d, *J* = 15.9 Hz, 1H, -CH=), 7.36–7.23 (m, 4H, Ar-H), 7.23–7.13 (m, 3H, Ar-H), 7.07–6.96 (m, 2H, Ar-H and -CH=), 6.94 (d, *J* = 16.0 Hz, 1H, -CH=), 6.54 (d, *J* = 15.7 Hz, 1H, -CH=), 3.83 (s, 3H, Ar-OCH_3_), 2.81 (t, *J* = 7.6 Hz, 2H, -CH_2_), 2.63–2.54 (m, 2H, -CH_2_). ^13^C NMR (151 MHz, CDCl_3_) δ 189.1, 148.6, 146.3, 145.8, 143.0, 140.7, 129.7, 128.4, 128.3, 128.2, 126.1, 123.0, 122.3, 112.9, 110.4, 55.9, 34.4, 34.3. ESI-HRMS (m/z): calcd. for C_20_H_20_O_3_Na [M+Na]^+^ 331.1310, found 331.1319. Purity: 98.9% (t_R_ = 10.27 min).

##### (1*E*,4*E*)-1-(4-hydroxy-3-methoxyphenyl)-7-phenyl-1,4-heptadien-3-one (**CU-9**)

Yellow solid; yield: 82.7%; m.p.: 114–116 °C. ^1^H NMR (600 MHz, DMSO-*d*_6_) δ 9.66 (s, 1H, Ar-OH), 7.56 (d, *J* = 15.9 Hz, 1H, -CH=), 7.36 (d, *J* = 2.0 Hz, 1H, Ar-H), 7.31 (t, *J* = 7.5 Hz, 2H, Ar-H), 7.26 (d, *J* = 6.6 Hz, 2H, Ar-H), 7.20 (t, *J* = 7.2 Hz, 1H, Ar-H), 7.18 (dd, *J* = 8.2, 2.0 Hz, 1H, Ar-H), 7.07–6.98 (m, 2H, -CH= and -CH=), 6.83 (d, *J* = 8.1 Hz, 1H, Ar-H), 6.54 (d, *J* = 15.7 Hz, 1H, -CH=), 3.84 (s, 3H, Ar-OCH_3_), 2.81 (t, *J* = 7.7 Hz, 2H, -CH_2_), 2.61–2.56 (m, 2H, -CH_2_). ^13^C NMR (151 MHz, CDCl_3_) δ 189.2, 148.2, 146.4, 143.5, 140.8, 129.5, 128.5, 128.4, 127.3, 126.1, 123.4, 122.8, 114.8, 109.7, 55.9, 34.5, 34.4. ESI-HRMS (m/z): calcd. for C_20_H_20_O_3_Na [M+Na]^+^ 331.1310, found 331.1317. Purity: 96.7% (t_R_ = 11.38 min).

##### (1*E*,4*E*)-1-(4-chlorophenyl)-7-phenyl-1,4-heptadien-3-one (**CU-10**)

Pale yellow solid; yield: 80.2%; m.p.: 70–72 °C. ^1^H NMR (600 MHz, DMSO-*d*_6_) δ 7.79 (dd, *J* = 8.8, 2.0 Hz, 2H, Ar-H), 7.61 (d, *J* = 16.0 Hz, 1H, -CH=), 7.53–7.48 (m, 2H, Ar-H), 7.31–7.24 (m, 5H, -CH= and Ar-H), 7.19 (t, *J* = 7.2 Hz, 1H, Ar-H), 7.09 (dt, *J* = 15.7, 6.8 Hz, 1H, -CH=), 6.52 (d, *J* = 15.8 Hz, 1H, -CH=), 2.81 (t, *J* = 7.7 Hz, 2H, -CH_2_), 2.61–2.57 (m, 2H, -CH_2_). ^13^C NMR (151 MHz, CDCl_3_) δ 188.9, 147.1, 141.7, 140.7, 136.2, 133.3, 129.7, 129.4, 129.2, 128.5, 128.3, 126.2, 125.1, 34.4, 34.4. ESI-HRMS (m/z): calcd. for C_19_H_18_ClO [M+H]^+^ 297.1046, found 297.1039. Purity: 96.0% (t_R_ = 16.00 min).

##### (1E,4E)-1-(4-hydroxyphenyl)-7-(p-tolyl)-1,4-heptadien-3-one (**CU-11**)

Yellow solid; yield: 83.4%; m.p.: 132–134 °C. ^1^H NMR (600 MHz, CDCl_3_) δ 7.51 (d, *J* = 15.9 Hz, 1H, -CH=), 7.41 (d, *J* = 8.6 Hz, 2H, Ar-H), 7.06–7.00 (m, 4H, Ar-H), 6.94 (dt, *J* = 15.5, 6.8 Hz, 1H, -CH=), 6.79 (d, *J* = 8.6 Hz, 2H, Ar-H), 6.75 (d, *J* = 15.8 Hz, 1H, -CH=), 6.37 (d, *J* = 15.7 Hz, 1H, -CH=), 5.31 (s, 1H, Ar-OH), 2.72 (t, *J* = 7.8 Hz, 2H, -CH_2_), 2.54–2.48 (m, 2H, -CH_2_), 2.25 (s, 3H, Ar-CH_3_). ^13^C NMR (151 MHz, CDCl_3_) δ 190.1, 158.7, 147.3, 144.1, 137.7, 135.6, 130.4, 129.4, 129.2, 128.2, 127.0, 122.2, 116.1, 34.6, 34.0, 21.0. ESI-HRMS (m/z): calcd. for C_20_H_20_O_2_Na [M+Na]^+^ 315.1361, found 315.1360. Purity: 97.9% (t_R_ = 8.69 min).

##### (1*E*,4*E*)-7-(4-hydroxy-3-methoxyphenyl)-1-(4-hydroxyphenyl)-1,4-heptadien-3-one (**CU-12**)

Grayish yellow solid; yield: 81.2%; m.p.: 122–124 °C. ^1^H NMR (600 MHz, DMSO-*d*_6_) δ 10.03 (s, 1H, Ar-OH), 8.69 (s, 1H, Ar-OH), 7.62–7.57 (m, 2H, Ar-H), 7.54 (d, *J* = 16.0 Hz, 1H, -CH=), 7.03–6.94 (m, 2H, -CH= and -CH=), 6.83–6.79 (m, 3H, Ar-H), 6.68 (d, *J* = 7.9 Hz, 1H, Ar-H), 6.62 (dd, *J* = 8.0, 2.0 Hz, 1H, Ar-H), 6.51 (d, *J* = 15.7 Hz, 1H, -CH=), 3.74 (s, 3H, Ar-OCH_3_), 2.69 (t, *J* = 7.6 Hz, 2H, -CH_2_), 2.55–2.52 (m, 2H, -CH_2_). ^13^C NMR (151 MHz, DMSO-*d*_6_) δ 188.5, 160.3, 147.7, 146.8, 145.0, 143.2, 132.1, 130.8, 129.8, 126.0, 122.1, 120.7, 116.1, 115.6, 112.9, 55.8, 34.4, 33.8. ESI-HRMS (m/z): calcd. for C_20_H_20_O_4_Na [M+Na]^+^ 347.1259, found 347.1260. Purity: 98.8% (t_R_ = 8.97 min).

##### (1*E*,4*E*)-1-(4-hydroxyphenyl)-7-(4-methoxyphenyl)-1,4-heptadien-3-one (**CU-13**)

Yellow solid; yield: 83.8%; m.p.: 136–138 °C. ^1^H NMR (600 MHz, CDCl_3_) δ 7.59 (d, *J* = 15.9 Hz, 1H, -CH=), 7.46 (d, *J* = 8.5 Hz, 2H, Ar-H), 7.11 (d, *J* = 8.5 Hz, 2H, Ar-H), 7.02 (dt, *J* = 15.6, 6.9 Hz, 1H, -CH=), 6.89 (d, *J* = 8.5 Hz, 2H, Ar-H), 6.84 (d, *J* = 8.5 Hz, 2H, Ar-H), 6.81 (d, *J* = 15.9 Hz, 1H, -CH=), 6.45 (d, *J* = 15.6 Hz, 1H, -CH=), 3.78 (s, 3H, Ar-OCH_3_), 2.76 (t, *J* = 7.7 Hz, 2H, -CH_2_), 2.60–2.53 (m, 2H, -CH_2_). ^13^C NMR (151 MHz, CDCl_3_) δ 190.0, 158.7, 157.9, 147.2, 144.0, 130.4, 129.5, 129.3, 122.2, 116.1, 113.9, 55.3, 34.7, 33.6. ESI-HRMS (m/z): calcd. for C_20_H_20_O_3_Na [M+Na]^+^ 331.1310, found 331.1319. Purity: 98.6% (t_R_ = 11.22 min).

##### (1*E*,4*E*)-1,7-bis(4-methoxyphenyl)-1,4-heptadien-3-one (**CU-14**)

Pale yellow solid; yield: 80.1%; m.p.: 65–67 °C. ^1^H NMR (600 MHz, CDCl_3_) δ 7.58 (d, *J* = 15.9 Hz, 1H, -CH=), 7.52 (d, *J* = 8.7 Hz, 2H, Ar-H), 7.12 (d, *J* = 8.6 Hz, 2H, Ar-H), 6.99 (dt, *J* = 15.6, 6.9 Hz, 1H, -CH=), 6.91 (d, *J* = 8.8 Hz, 2H, Ar-H), 6.84 (d, *J* = 8.6 Hz, 2H, Ar-H), 6.82 (d, *J* = 15.9 Hz, 1H, -CH=), 6.43 (d, *J* = 15.6 Hz, 1H, -CH=), 3.85 (s, 3H, Ar-OCH_3_), 3.79 (s, 3H, Ar-OCH_3_), 2.77 (t, *J* = 7.7 Hz, 2H, -CH_2_), 2.58–2.55 (m, 2H, -CH_2_). ^13^C NMR (151 MHz, CDCl_3_) δ 189.1, 161.4, 146.3, 142.9, 132.8, 129.9, 129.6, 129.2, 127.4, 122.7, 114.3, 113.8, 55.3, 55.1, 34.5, 33.5. ESI-HRMS (m/z): calcd. for C_21_H_22_O_3_Na [M+Na]^+^ 345.1467, found 345.1466. Purity: 98.8% (t_R_ = 10.67 min).

##### (1*E*,4*E*)-1,7-diphenyl-1,4-heptadien-3-one (**CU-15**)

Pale yellow solid; yield: 53.4%; m.p.: 28–30 °C. ^1^H NMR (600 MHz, DMSO-*d*_6_) δ 7.76–7.75 (m, 2H, Ar-H), 7.62 (d, *J*= 15.96 Hz, 1H, -CH=), 7.45–7.43 (m, 3H, Ar-H), 7.30 (t, *J*= 7.5 Hz, 2H, Ar-H), 7.26–7.25 (m, 2H, Ar-H), 7.23 (d, *J* = 16.1 Hz, 1H, -CH=), 7.20 (t, *J* = 7.2 Hz, 1H, Ar-H), 7.07 (dt, *J* = 15.7, 6.7 Hz, 1H, -CH=), 6.55 (d, *J* = 15.7 Hz, 1H, -CH=), 2.81 (t, *J* = 7.7 Hz, 2H, -CH_2_), 2.62–2.56 (m, 2H, -CH_2_); ^13^C NMR (151 MHz, CDCl_3_) δ 189.2, 146.8, 143.2, 140.7, 134.7, 130.3, 129.5, 128.8, 128.4, 128.3, 128.2, 126.1, 124.7, 34.4, 34.3. ESI-HRMS (m/z): calcd. for C_19_H_19_O[M+H]^+^ 263.1433, found 263.1436, calcd. for C_19_H_18_ONa [M+Na]^+^ 285.1255, found 285.1259. Purity: 98.8% (t_R_ = 17.36 min).

##### (1*E*,4*E*)-7-phenyl-1-(p-tolyl)-1,4- heptadien-3-one (**CU-16**)

Pale yellow solid; yield: 55.5%; m.p.: 46–48 °C. ^1^H NMR (600 MHz, CDCl_3_) δ 7.59 (d, *J* = 15.9 Hz, 1H, -CH=), 7.46 (d, *J* = 7.9 Hz, 2H, Ar-H), 7.31 (t, *J* = 7.5 Hz, 2H, Ar-H), 7.23–7.20 (m, 5H, Ar-H), 7.02 (dt, *J* = 15.4, 6.8 Hz, 1H, -CH=), 6.90 (d, *J* = 15.9 Hz, 1H, -CH=), 6.45 (d, *J* = 15.7 Hz, 1H, -CH=), 2.83 (t, *J* = 7.8 Hz, 2H, -CH_2_), 2.66–2.57 (m, 2H, -CH_2_), 2.38 (s, 3H, Ar-CH_3_). ^13^C NMR (151 MHz, CDCl_3_) δ 189.2, 146.5, 143.2, 140.9, 140.8, 132.0, 129.6, 128.5, 128.4, 128.3, 126.2, 124.0, 34.5, 34.4, 21.5. ESI-HRMS (m/z): calcd. for C_20_H_20_ONa [M+Na]^+^ 299.1412, found 299.1413. Purity: 99.6% (t_R_ = 13.31 min).

##### (1*E*,4*E*)-1-(4-hydroxy-3-methoxyphenyl)-7-(4-hydroxyphenyl)-1,4-heptadien-3-one (**CU-17**)

Red solid; yield: 83.2%; m.p.: 141–143 °C. ^1^H NMR (600 MHz, DMSO- d_6_) δ 9.63 (s, 1H, Ar-OH), 9.15 (s, 1H, Ar-OH), 7.54 (d, *J* = 15.9 Hz, 1H, -CH=), 7.34 (d, *J* = 1.9 Hz, 1H, Ar-H), 7.16 (dd, *J* = 8.1, 2.0 Hz, 1H, Ar-H), 7.04–7.01 (m, 3H, Ar-H and -CH=), 6.98 (dt, *J* = 15.6, 6.7 Hz, 1H, -CH=), 6.81 (d, *J* = 8.1 Hz, 1H, Ar-H), 6.68 (d, *J* = 8.2 Hz, 2H, Ar-H), 6.51 (d, *J* = 15.6 Hz, 1H, -CH=), 3.83 (s, 3H, Ar-OCH_3_), 2.68 (t, *J* = 7.7 Hz, 2H, -CH_2_), 2.53–2.49 (m, 2H, -CH_2_). ^13^C NMR (151 MHz, DMSO-*d*_6_) δ 188.5, 155.8, 149.8, 148.3, 146.7, 143.6, 131.4, 129.8, 129.5, 126.5, 123.8, 122.4, 115.9, 115.4, 111.7, 56.0, 34.4, 33.3. ESI-HRMS (m/z): calcd. for C_20_H_20_O_4_Na [M+Na]^+^ 347.1259, found 347.1264. Purity: 98.9% (t_R_ = 9.05 min).

##### (1*E*,4*E*)-1-(3-hydroxy-4-methoxyphenyl)-7-(4-hydroxyphenyl)-1,4-heptadien-3-one (**CU-18**)

Yellow solid; yield: 83.6%; m.p.: 152–154 °C. ^1^H NMR (600 MHz, DMSO-*d*_6_) δ 9.17 (s, 1H, Ar-OH), 9.15 (s, 1H, Ar-OH), 7.50 (d, *J* = 15.9 Hz, 1H, -CH=), 7.17–7.15 (m, 2H, Ar-H), 7.03 (d, *J* = 8.4 Hz, 2H, Ar-H), 7.00–6.96 (m, 2H, Ar-H and -CH=), 6.94 (d, *J* = 16.0 Hz, 1H, -CH=), 6.68 (d, *J* = 8.4 Hz, 2H, Ar-H), 6.52 (d, *J* = 15.7 Hz, 1H, -CH=), 3.82 (s, 3H, Ar-OCH_3_), 2.68 (t, *J* = 7.6 Hz, 2H, -CH_2_), 2.52–2.48 (m, 2H, -CH_2_). ^13^C NMR (151 MHz, DMSO-*d*_6_) δ 188.5, 155.8, 150.4, 147.0, 147.0, 143.2, 131.3, 129.7, 129.5, 127.8, 123.0, 121.9, 115.4, 114.7, 112.2, 56.0, 34.4, 33.3. ESI-HRMS (m/z): calcd. for C_20_H_20_O_4_Na [M+Na]^+^ 347.1259, found 347.1261. Purity: 99.0% (t_R_ = 8.28 min).

##### (1*E*,4*E*)-1-(3,4-dimethoxyphenyl)-7-phenyl-1,4-heptadien-3-one (**CU-19**)

Yellow oil; yield: 53.6%; ^1^H NMR (600 MHz, CDCl_3_) δ 7.57 (d, *J* = 15.9 Hz, 1H, -CH=), 7.32–7.29 (m, 2H, Ar-H), 7.23–7.20 (m, 3H, Ar-H), 7.15 (dd, *J* = 8.3, 2.0 Hz, 1H, Ar-H), 7.09 (d, *J* = 2.0 Hz, 1H, Ar-H), 7.02 (dt, *J* = 15.6, 6.9 Hz, 1H, -CH=), 6.88 (d, *J* = 8.3 Hz, 1H, Ar-H), 6.81 (d, *J* = 15.9 Hz, 1H, -CH=), 6.47 (dt, *J* = 15.6, 1.6 Hz, 1H, -CH=), 3.93 (s, 3H, Ar-OCH_3_), 3.92 (s, 3H, Ar-OCH_3_), 2.84 (t, *J* = 7.3 Hz, 2H, -CH_2_), 2.63–2.59 (m, 2H, -CH_2_). ^13^C NMR (151 MHz, CDCl_3_) δ 189.0, 146.3, 143.2, 140.7, 129.4, 128.4, 128.3, 127.6, 126.0, 123.0, 111.0, 109.7, 55.9, 55.8, 34.4, 34.3. ESI-HRMS (m/z): calcd. for C_21_H_22_O_3_Na [M+Na]^+^ 345.1467, found 345.1480. Purity: 99.8% (t_R_ = 21.02 min).

##### (1*E*,4*E*)-1,7-bis(4-hydroxy-3-methoxyphenyl)-1,4-heptadien-3-one (**CU-20**)

Yellow solid; yield: 80.4%; m.p.: 103–105 °C. ^1^H NMR (600 MHz, DMSO-*d*_6_) δ 9.64 (s, 1H, Ar-OH), 8.70 (s, 1H, Ar-OH), 7.55 (d, *J* = 15.9 Hz, 1H, -CH=), 7.35 (d, *J* = 2.0 Hz, 1H, Ar-H), 7.17 (dd, *J* = 8.2, 2.0 Hz, 1H, Ar-H), 7.04 (d, *J* = 16.0 Hz, 1H, -CH=), 7.00 (dt, *J* = 15.6, 6.7 Hz, 1H, -CH=), 6.82–6.80 (m, 2H, Ar-H), 6.68 (d, *J* = 7.9 Hz, 1H, Ar-H), 6.63 (dd, *J* = 8.0, 2.0 Hz, 1H, Ar-H), 6.53 (d, *J* = 15.7 Hz, 1H, -CH=), 3.84 (s, 3H, Ar-OCH_3_), 3.75 (s, 3H, Ar-OCH_3_), 2.70 (dd, *J* = 8.7, 6.6 Hz, 2H, -CH_2_), 2.56–2.52 (m, 2H, -CH_2_). ^13^C NMR (151 MHz, CDCl_3_) δ 189.1, 148.1, 146.7, 146.4, 146.3, 143.8, 143.5, 132.7, 129.3, 127.2, 123.2, 122.7, 120.8, 114.7, 114.2, 110.9, 109.6, 55.8, 55.8, 34.6, 34.1. ESI-HRMS (m/z): calcd. for C_21_H_22_O_5_Na [M+Na]^+^ 377.1365, found 377.1380. Purity: 98.5% (t_R_ = 6.89 min).

##### (1*E*,4*E*)-7-(4-methoxyphenyl)-1-phenyl-1,4-heptadien-3-one (**CU-21**)

Pale yellow solid; yield: 51.6%; m.p.: 58–60 °C. ^1^H NMR (600 MHz, CDCl_3_) δ 7.61 (d, *J* = 15.9 Hz, 1H, -CH=), 7.56 (dd, *J* = 6.6, 2.9 Hz, 2H, Ar-H), 7.40-7.39 (m, 3H, Ar-H), 7.12 (d, *J* = 8.4 Hz, 2H, Ar-H), 7.02 (dt, *J* = 15.7, 6.9 Hz, 1H, -CH=), 6.94 (d, *J* = 15.9 Hz, 1H, -CH=), 6.85 (d, *J* = 8.5 Hz, 2H, Ar-H), 6.44 (d, *J* = 15.7 Hz, 1H, -CH=), 3.79 (s, 3H, -OCH_3_), 2.78 (t, *J* = 7.7 Hz, 2H, -CH_2_), 2.60–2.55 (m, 2H, -CH_2_). ^13^C NMR (151 MHz, CDCl_3_) δ 189.1, 146.9, 143.0, 132.7, 130.3, 129.5, 129.2, 128.8, 128.2, 124.8, 113.8, 55.1, 34.6, 33.5. ESI-HRMS (m/z): calcd. for C_20_H_20_O_2_Na [M+Na]^+^ 315.1361, found 315.1368. Purity: 98.7% (t_R_ = 11.03 min).

##### (1*E*,4*E*)-1-phenyl-7-(p-tolyl)-1,4-heptadien-3-one (**CU-22**)

Pale yellow solid; yield: 54.9%; m.p.: 62–64 °C. ^1^H NMR (600 MHz, DMSO-*d*_6_) δ 7.77–7.73 (m, 2H, Ar-H), 7.62 (d, *J* = 16.0 Hz, 1H, -CH=), 7.45–7.42 (m, 3H, Ar-H), 7.23 (d, *J* = 16.1 Hz, 1H, -CH=), 7.13 (d, *J* = 8.0 Hz, 2H, Ar-H), 7.11–7.04 (m, 3H, Ar-H and -CH=), 6.53 (d, *J* = 15.7 Hz, 1H, -CH=), 2.76 (t, *J* = 7.6 Hz, 2H, -CH_2_), 2.58–2.54 (m, 2H, -CH_2_), 2.26 (s, 3H, Ar-CH_3_). ^13^C NMR (151 MHz, CDCl_3_) δ 189.2, 147.0, 143.1, 137.7, 135.6, 134.8, 130.4, 129.6, 129.2, 128.9, 128.3, 128.2, 124.8, 34.5, 34.0, 21.0. ESI-HRMS (m/z): calcd. for C_20_H_21_O [M+H]^+^ 277.1592, found 277.1584. Purity: 99.0% (t_R_ = 10.49 min).

##### (1*E*,4*E*)-7-(3,4-dimethoxyphenyl)-1-phenyl-1,4-heptadien-3-one (**CU-23**)

Pale yellow oil; yield: 53.5%; ^1^H NMR (600 MHz, CDCl_3_) δ 7.62 (d, *J* = 16.0 Hz, 1H, -CH=), 7.59–7.55 (m, 2H, Ar-H), 7.40-7.39 (m, 3H, Ar-H), 7.03 (dt, *J* = 15.7, 6.9 Hz, 1H, -CH=), 6.94 (d, *J* = 16.0 Hz, 1H, -CH=), 6.81 (d, *J* = 8.1 Hz, 1H, Ar-H), 6.75–6.72 (m, 2H, Ar-H), 6.45 (d, *J* = 15.7 Hz, 1H, -CH=), 3.88 (s, 3H, -OCH_3_), 3.86 (s, 3H, -OCH_3_), 2.79 (t, *J* = 7.7 Hz, 2H, -CH_2_), 2.62–2.57 (m, 2H, -CH_2_). ^13^C NMR (151 MHz, CDCl_3_) δ 189.1, 148.8, 146.8, 143.1, 133.3, 130.3, 129.5, 128.8, 128.2, 124.7, 120.1, 111.6, 111.2, 55.8, 55.7, 34.5, 34.0. ESI-HRMS (m/z): calcd. for C_21_H_22_O_3_Na [M+Na]^+^ 345.1467, found 345.1467. Purity: 97.8% (t_R_ = 6.99 min).

##### (1*E*,4*E*)-7-(3-hydroxyphenyl)-1-phenyl-1,4-heptadien-3-one (**CU-24**)

Pale yellow solid; yield: 81.7%; m.p.: 70–72 °C. ^1^H NMR (600 MHz, DMSO-*d*_6_) δ 9.27 (s, 1H, Ar-OH), 7.77–7.74 (m, 2H, Ar-H), 7.62 (d, *J* = 16.0 Hz, 1H, -CH=), 7.44–7.43 (m, 3H, Ar-H), 7.23 (d, *J* = 15.8 Hz, 1H, -CH=), 7.09–7.04 (m, 2H, Ar-H and -CH=), 6.66 (d, *J* = 7.4 Hz, 1H, Ar-H), 6.63 (s, 1H, Ar-H), 6.58 (d, *J* = 8.0 Hz, 1H, Ar-H), 6.54 (d, *J* = 15.9 Hz, 1H, -CH=), 2.70 (t, *J* = 8.1 Hz, 2H, -CH_2_), 2.57–2.53 (m, 2H, -CH_2_). ^13^C NMR (151 MHz, CDCl_3_) δ 189.9, 156.1, 147.6, 143.8, 142.5, 134.6, 130.6, 129.7, 129.5, 128.9, 128.4, 124.7, 120.5, 115.4, 113.3, 34.3, 34.2. ESI-HRMS (m/z): calcd. for C_19_H_18_O_2_Na [M+H]^+^ 279.1385, found 279.1396. Purity: 99.2% (t_R_ = 7.89 min).

##### (1*E*,4*E*)-7-(4-hydroxyphenyl)-1-phenyl-1,4-heptadien-3-one (**CU-25**)

Red solid; yield: 81.2%; m.p.: 75–77 °C. ^1^H NMR (600 MHz, DMSO-*d*_6_) δ 9.16 (s, 1H, Ar-OH), 7.76–7.75 (m, 2H, Ar-H), 7.62 (d, *J* = 16.1 Hz, 1H, -CH=), 7.46–7.44 (m, 3H, Ar-H), 7.23 (d, *J* = 16.0 Hz, 1H, -CH=), 7.08–7.02 (m, 3H, Ar-H and -CH=), 6.69–6.67 (m, 2H, Ar-H), 6.52 (d, *J* = 15.7 Hz, 1H, -CH=), 2.69 (t, *J* = 8.0 Hz, 2H, -CH_2_), 2.54–2.51 (m, 2H, -CH_2_). ^13^C NMR (151 MHz, CDCl_3_) δ 189.7, 154.2, 147.5, 143.6, 130.5, 129.5, 129.4, 128.9, 128.3, 124.7, 115.4, 34.7, 33.6. ESI-HRMS (m/z): calcd. for C_19_H_18_O_2_Na [M+H]^+^ 279.1385, found 279.1394. Purity: 98.9% (t_R_ = 6.61 min).

##### (1*E*,4*E*)-7-(4-hydroxy-3-methoxyphenyl)-1-phenyl-1,4-heptadien-3-one (**CU-26**)

Pale yellow solid; yield: 80.1%; m.p.: 72–74 °C. ^1^H NMR (600 MHz, DMSO-*d*_6_) δ 8.80 (s, 1H, Ar-OH), 7.75 (dd, *J* = 6.7, 2.9 Hz, 2H, Ar-H), 7.62 (d, *J* = 16.1 Hz, 1H, -CH=), 7.46–7.43 (m, 3H, Ar-H), 7.22 (d, *J* = 16.1 Hz, 1H, -CH=), 7.05 (dt, *J* = 15.7, 6.7 Hz, 1H, -CH=), 6.83 (d, *J* = 8.2 Hz, 1H, Ar-H), 6.67 (d, *J* = 2.1 Hz, 1H, Ar-H), 6.62 (dd, *J* = 8.2, 2.1 Hz, 1H, Ar-H), 6.54 (d, *J* = 15.8 Hz, 1H, -CH=), 3.73 (s, 3H, Ar-OCH_3_), 2.67 (t, *J* = 7.6 Hz, 2H, -CH_2_), 2.55–2.52 (m, 2H, -CH_2_). ^13^C NMR (151 MHz, CDCl_3_) δ 147.0, 145.6, 145.0, 143.1, 134.8, 134.1, 130.3, 129.6, 128.9, 128.3, 124.8, 119.7, 114.5, 110.7, 56.0, 34.5, 33.8. ESI-HRMS (m/z): calcd. for C_20_H_20_O_3_Na [M+Na]^+^ 331.131, found 331.1316. Purity: 99.0% (t_R_ = 15.05 min).

##### (1*E*,4*E*)-1-phenyl-7-(3,4,5-trimethoxyphenyl)-1,4-heptadien-3-one (**CU-27**)

Pale yellow oil; yield: 50.3%. ^1^H NMR (600 MHz, CDCl_3_) δ 7.63 (d, *J* = 16.0 Hz, 1H, -CH=), 7.57 (dd, *J* = 6.7, 3.0 Hz, 2H, Ar-H), 7.41–7.40 (m, 3H, Ar-H), 7.04 (dt, *J* = 15.6, 6.9 Hz, 1H, -CH=), 6.95 (d, *J* = 15.9 Hz, 1H, -CH=), 6.48 (d, *J* = 15.7 Hz, 1H, -CH=), 6.42 (s, 2H, Ar-H), 3.85 (s, 6H, Ar-OCH_3_), 3.83 (s, 3H, Ar-OCH_3_), 2.78 (t, *J* = 7.7 Hz, 2H, -CH_2_), 2.63–2.59 (m, 2H, -CH_2_). ^13^C NMR (151 MHz, CDCl_3_) δ 153.1, 146.8, 143.3, 136.2, 134.6, 130.4, 129.5, 128.8, 128.6, 128.2, 127.1, 124.7, 105.2, 60.7, 56.0, 34.8, 34.4. ESI-HRMS (m/z): calcd. for C_22_H_24_O_4_Na [M+Na]^+^ 375.1572, found 375.1569. Purity: 97.7% (t_R_ = 10.33 min).

##### (1*E*,4*E*)-7-(4-chlorophenyl)-1-phenyl-1,4-heptadien-3-one (**CU-28**)

Pale yellow solid; yield: 56.8%; m.p.: 48–50 °C. ^1^H NMR (600 MHz, CDCl_3_) δ 7.61 (d, *J* = 16.0 Hz, 1H, -CH=), 7.57 (dd, *J* = 6.7, 2.9 Hz, 2H, Ar-H), 7.41–7.39 (m, 3H, Ar-H), 7.27 (d, *J* = 8.4 Hz, 2H, Ar-H), 7.13 (d, *J* = 8.3 Hz, 2H, Ar-H), 6.99 (dt, *J* = 15.6, 6.8 Hz, 1H, -CH=), 6.93 (d, *J* = 16.0 Hz, 1H, -CH=), 6.44 (d, *J* = 15.6 Hz, 1H, -CH=), 2.81 (t, *J* = 7.7 Hz, 2H, -CH_2_), 2.60–2.57 (m, 2H, -CH_2_). ^13^C NMR (151 MHz, CDCl_3_) δ 189.0, 151.3, 149.2, 147.4, 146.4, 143.3, 133.4, 129.4, 127.7, 123.1, 123.0, 120.2, 111.7, 111.3, 111.0, 109.8, 34.6, 34.1. ESI-HRMS (m/z): calcd. for C_19_H_17_ClONa [M+Na]^+^ 319.0866, found 319.0875. Purity: 97.9% (t_R_ = 17.37 min).

### 4.2. Biological Evaluation

#### 4.2.1. Cell Culture

The BV2 microglial cells were purchased from the China Center for Type Culture Collection, CCTCC (Wuhan, Hubei). BV2 microglia cells were incubated in DMEM media supplemented with 10% (*v/v*) FBS, 100 U/mL penicillin G and 100 mg/mL streptomycin at 37 °C with 5% CO_2_.

#### 4.2.2. Determination of Cell Viability

Cell viability assays were evaluated using a 3-(4,5-dimethylthiazol-2-yl)-2,5-diphenyltetrazolium bromide (MTT) assay. BV2 microglia cells were seeded in 96-well plates at a density of 1.6 × 10^5^ cells/well in complete medium and incubated for 24 h. Then, the culture medium was sucked and discarded, and sterile PBS was added for washing, and then PBS was removed. Then the cells were treated with different concentrations of target compounds for 24 h. An amount equal to 100 μL of MTT was added to each well and the cells were further incubated for 2–4 h. An amount equal to 150 μL of DMSO was added into a 96-well plate and vibrated with micro-oscillator for 10 min. The optical density (A) was measured at 630 nm on a microplate reader. The inhibitory rate of the various concentrations of agents in BV2 cells was calculated using the following formula:

Cell viability (%) = [A492 (sample) − A492 (blank)]/[A492 (control) − A492 (blank)] × 100%

#### 4.2.3. Assay for NO Production

Nitrite, a stable product of nitric oxide, was used to assess NO production. To study the effects of the compounds on NO production, BV2 microglia cells in the logarithmic phase were plated at 1 × 10^5^ cells/well in a 24-well microplate and incubated in DMEM medium (10% FBS) overnight. The cells were pretreated with various concentrations of the test compounds for 1 h and further co-cultured with 1 μg/mL LPS and 5% CO_2_ for 24 h in a 37 °C incubator; then, the supernatant was taken and stored at −20 °C for NO detection. The target compound and LPS were configured with DMEM medium (2% FBS). The determination of NO was carried out according to the instructions. Unpretreated and unstimulated BV2 microglia cells were conducted as the blank control group. The absorbance value was determined at 540 nm, and the corresponding NO content was calculated by using the standard curve. The NO inhibitory rate of the various concentrations of agents in BV2 cells was calculated as per the following formula:

Inhibition rate (%) = (LPS_(NO concentration)_ − sample_(NO concentration)_)/(LPS_(NO concentration)_ − negative control_(NO concentration)_) × 100%.

#### 4.2.4. Inflammatory Mediator Assays

BV2 microglia cells were seeded at 1 × 10^5^ cells/well in a 24-well microplate and incubated overnight. The cells were pretreated with 3 µM of **CU-19**, **CU-21** and curcumin for 1 h and then stimulated with 1 μg/mL of LPS for 24 h. **CU-19**, **CU-21** and LPS were configured with DMEM medium (2% FBS). According to the protocol of the manufacturer, supernatant was harvested at the 24th hour for the assay of IL-6, TNF-*α* and PGE-2 with ELISA kits (Absin Shanghai, China). Briefly, 100 μL of biotinylated antibody reagent and the culture supernatant were added to anti-mouse IL-6, TNF-*α* and PGE-2-precoated 96-well plates, and the plates were incubated for 2.5 h at room temperature. The plate was washed with a washing buffer and subsequently incubated with 100 μL of the streptavidin–HRP solution for 20 min at room temperature. The plate was washed and incubated with 100 μL of TMB substrate solution for 20 min at room temperature in the dark. The reaction was stopped by adding 50 μL of stop solution, and then the absorbance was measured at 450 nm by a plate reader. The average value was substituted into the standard curve to obtain the antibody concentration.

#### 4.2.5. Western Blot Analysis

BV2 microglia cells (5 × 10^5^ cells/well) plated onto 6-well plates were incubated for 18 h and treated with 3.0 µM of **CU-19**, **CU-21** and curcumin for 1 h and then stimulated with 1.0 μg/mL of LPS for 24 h. **CU-19**, **CU-21** and LPS were configured with DMEM medium (2% FBS). The cells were collected and washed three times with ice-cold PBS. The lysate (containing 1% protease inhibitor and phosphatase inhibitor) was added to the collected cells for suspension, and the cells were lysed by ultrasonic crusher. Then the cell lysates were centrifuged (14,000 rpm, 10 min, 4 °C) for 5 min and collect the supernatant. Aliquots of the lysates were separated on 10% sodium dodecyl sulphate (SDS)-polyacrylamide gel electrophoresis (PAGE) and then electro blotted onto a polyvinylidene difluoride (PVDF) membrane. The blots were blocked with 5% (*w/v*) nonfat dry milk for 2 h at room temperature, followed by incubation with specific primary antibodies at 4 °C overnight. Blots were washed with PBST and incubated with secondary antibody for 2 h, after which an ECL reagent was used for chemiluminescent detection.

#### 4.2.6. Immunofluorescent Staining

Cells were added to 6-well plates (5 × 10^5^/well) for 24 h, after which they were treated with a combination of LPS (1 mg/mL) and 3.0 µM of **CU-19**, **CU-21** and curcumin. **CU-19**, **CU-21** and LPS were configured with DMEM medium (2% FBS). After 24 h of treatment, cells were fixed with 4% paraformaldehyde for 18 min and washed three times with PBS (5 min per wash). The fixed cells were then permeabilized in 1% Triton X-100 (0.15%) for 15 min and washed three times with PBS (5 min per wash). Nonspecific binding sites were blocked with 5% goat serum for 30 min. These cells were then incubated with anti-NF-κB antibody at a dilution of 1:400 overnight at 4 °C. After washing with PBS three times, cells were incubated with fluorescent-conjugated secondary antibody (1:150) for 1 h, and counterstained with DAPI (1:50) at room temperature for 15 min in the dark. Cells were then washed and imaged via confocal microscopy.

#### 4.2.7. Statistical Analysis

Statistical analyses were realized by using GraphPad Prism 6.0 software (GraphPad Software, San Diego, CA, USA). Significant differences between the groups were determined by one-way and two-way analyses of variance (ANOVA), which were followed by Fisher’s LSD tests for multiple comparisons. Obtained results were presented as mean ± standard deviation (SD) of independently performed experiments, and the experiment was repeated at least three times. Differences between the datasets were accepted as significant when *p* value < 0.05.

### 4.3. In Vivo Pharmacokinetic Study in Rats

Sprague–Dawley male rats, 200–240 g, were housed in cages in an airconditioned room with light dark cycle of 12 h before the experiment started. The animals were fasted for 12 h with water ad libitum. The rats were divided into 3 groups each of 3 animals. Doses of **CU**, **CU-19** and **CU-21**, were calculated as 20 mg compound per kg of body weight. The administration volume was 6 mL per kg of body weight. The compounds were dissolved in PEG400 (10% of the administration volume) and water (90% of the administration volume) and sonicated for 30 min. After tail vein injection administration at a dose of 20 mg/kg, 0.5 mL blood was obtained from fossa orbitalis veniplex of rats at 5, 10, 20, 30, 45, 60, 120, 180 and 240 min. Whole blood samples were collected in heparinized tubes and the plasma was immediately centrifuged (10,000 rpm, 10 min, 4 °C) and then stored at −20 °C until analysis. The calibration standards and the plasma samples were extracted by protein precipitation using methanol. The concentrations of compounds in the extracted standards and plasma samples were quantified by HPLC with a reversed-phase column (Waters Spherisorb ODS column (250 × 4.6 mm, 5 μm) (column temperature = 30 ± 2 °C) and methanol: 0.1% triethylamine (70:30 for **CU**, 80:20 for **CU-19** and **CU-21**) as the mobile phase. The flow rate was maintained at 1 mL/min and UV detection at 390 nm (**CU**), 340 nm (**CU-19)**, 300 nm (**CU-21**). The pharmacokinetics parameters were calculated using DAS software. **CU** was used as the internal standard of **CU-19** and **CU-21**; Kaempferol was used as the internal standard of **CU**.

## Figures and Tables

**Table 1:** The structures of the target compounds.

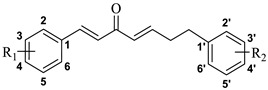					
Compd.	R_1_	R_2_	Compd.	R_1_	R_2_
**CU-1**	4-OH	4′-OH	**CU-15**	H	H
**CU-2**	3-OCH_3_, 4- OCH_3_	3′-OCH_3_, 4′- OCH_3_	**CU-16**	4-CH_3_	H
**CU-3**	4-OCH_3_	H	**CU-17**	3-OCH_3_, 4-OH	4′-OH
**CU-4**	3-OCH_3_, 4-OCH_3_, 5-OCH_3_	H	**CU-18**	3-OH, 4-OCH_3_	4′-OH
**CU-5**	2-OH	H	**CU-19**	3-OCH_3_, 4-OCH_3_	H
**CU-6**	3-OH	H	**CU-20**	3-OCH_3_, 4-OH	3′-OCH_3_, 4′-OH
**CU-7**	4-OH	H	**CU-21**	H	4′-OCH_3_
**CU-8**	3-OH, 4-OCH_3_	H	**CU-22**	H	4′-CH_3_
**CU-9**	3-OCH_3_, 4-OH	H	**CU-23**	H	3′-OCH_3_, 4′- OCH_3_
**CU-10**	4-Cl	H	**CU-24**	H	3′-OH
**CU-11**	4-OH	4′-CH_3_	**CU-25**	H	4′-OH
**CU-12**	4-OH	3′-OCH_3_, 4′-OH	**CU-26**	H	3′-OCH_3_, 4′-OH
**CU-13**	4-OH	4′-OCH_3_	**CU-27**	H	3′-OCH_3_, 4′-OCH_3_, 5′-OCH_3_
**CU-14**	4-OCH_3_	4′-OCH_3_	**CU-28**	H	4′-Cl

**Table 2:** The cell viability and inhibition of LPS-induced NO release in BV2 cells.

Compd.	Cell Viability (%) ^a^			NO Inhibition (%)	
	1 µM	3 µM	10 µM	1 µM	3 µM
**CU-1**	88.07 ± 0.19	49.84 ± 0.27	23.70 ± 0.38	0	-
**CU-2**	76.92 ± 0.19	49.16 ± 0.92	10.57 ± 1.13	0	-
**CU-4**	107.23 ± 0.09	73.92 ± 0.30	16.61 ± 0.45	3.10	-
**CU-5**	95.68 ± 0.11	77.39 ± 0.30	15.92 ± 0.61	0	-
**CU-7**	93.46 ± 0.35	52.80 ± 0.50	14.26 ± 0.70	0	-
**CU-12**	92.03 ± 0.29	75.55 ± 0.17	12.67 ± 0.59	0	-
**CU-17**	99.36 ± 0.01	80.72 ± 0.20	32.42 ± 0.67	0	-
**CU-18**	96.14 ± 0.03	46.56 ± 1.16	8.63 ± 0.373	2.15	-
**CU-20**	97.31 ± 0.03	51.82 ± 0.60	12.24 ± 0.21	16.9	-
**CU-3**	106.72 ± 0.14	101.55 ± 0.01	98.50 ± 0.01	-	35.2
**CU-6**	106.85 ± 0.10	100.07 ± 0.01	101.56 ± 0.03	-	22.1
**CU-8**	101.18 ± 0.04	97.40 ± 0.07	18.20 ± 0.86	-	18.8
**CU-9**	99.38 ± 0.01	94.65 ± 0.07	14.75 ± 0.75	-	7.52
**CU-10**	95.54 ± 0.03	95.27 ± 0.16	48.66 ± 2.93	-	0
**CU-11**	99.17 ± 0.01	91.61 ± 0.09	11.24 ± 0.56	-	0
**CU-13**	109.45 ± 0.27	91.40 ± 0.32	28.53 ± 1.30	-	0
**CU-14**	97.96 ± 0.01	97.44 ± 0.03	18.06 ± 0.10	-	49.7
**CU-15**	104.99 ± 0.03	103.11 ± 0.03	84.19 ± 0.21	-	13.7
**CU-16**	95.00 ± 0.08	88.57 ± 0.28	33.33 ± 1.20	-	15.4
**CU-19**	96.94 ± 0.03	96.33 ± 0.02	67.91 ± 0.37	-	50.3
**CU-21**	96.94 ± 0.01	94.55 ± 0.09	77.89 ± 0.50	-	66.1
**CU-22**	95.14 ± 0.05	88.76 ± 0.06	46.65 ± 0.59	-	48.2
**CU-23**	106.47 ± 0.14	100.85 ± 0.01	12.35 ± 0.84	-	31.4
**CU-24**	103.49 ± 0.02	91.48 ± 0.07	23.17 ± 0.09	-	0
**CU-25**	128.58 ± 0.14	96.93 ± 0.01	20.30 ± 0.59	-	0
**CU-26**	107.17 ± 0.07	96.83 ± 0.02	12.85 ± 0.14	-	37.6
**CU-27**	116.10 ± 0.44	101.31 ± 0.03	38.93 ± 1.19	-	7.02
**CU-28**	95.71 ± 0.07	94.75 ± 0.05	81.65 ± 0.22	-	33.6
**CU**	97.57 ± 0.05	95.26 ± 0.05	28.15 ± 0.19	-	37.4

^a^ Cell viability was the mean ± SD, *n* = 3.

**Table 3:** The IC_50_ value of active compounds to inhibit LPS-induced NO release in BV2 cells. Results were the mean ± SD, *n* = 3.

Compd.	CU-14	CU-19	CU-21	CU-22	CU
IC_50_ (μM)	2.43 ± 0.45	1.60 ± 0.31	1.74 ± 0.66	>3	>3

**Table 4:** Pharmacokinetic characteristics of **CU-19, CU-21** and **CU** in the plasma of healthy rats (*n* = 4), which were administrated a single intravenous dose of 20 mg compound per kg of body weight. Results are shown as the mean ± SD, *n* = 4.

**Parameter**	CU	CU-19	CU-21
C_max_/(μg·mL^−1^)	2.45 ± 0.132	3.33 ± 0.393	6.85 ± 0.634
T_1/2_/min	24.7 ± 16.907	135 ± 6.244	163 ± 36.772
CL (L/h/Kg)	0.500 ± 0.069	0.150 ± 0.021	0.100 ± 0.012
AUC _(0-t)_/(μg·min·mL^−1^)	39.1 ± 4.186	106 ± 11.908	170 ± 11.121
AUC _(0-∞)_/(μg·min·mL^−1^)	40.7 ± 5.663	133 ± 16.995	212 ± 24.724
MRT _(0-t)_/min	14.1 ± 3.862	61.2 ± 3.189	45.4 ± 2.659
MRT _(0-∞)_/min	20.5 ± 9.253	127 ± 3.423	139 ± 22.909

## Data Availability

Not applicable.

## Supplementary Materials

The following are available online at https://www.mdpi.com/article/10.3390/molecules27113537/s1, Figure S1. Representative chromatogram of the determination of CU after intravenous administration 5 min, Figure S2. Representative chromatogram of the determination of CU after intravenous administration 10 min, Figure S3. Representative chromatogram of the determination of CU after intravenous administration 20 min, Figure S4. Representative chromatogram of the determination of CU-19 after intravenous administration 5 min, Figure S5. Representative chromatogram of the determination of CU-19 after intravenous administration 10 min, Figure S6. Representative chromatogram of the determination of CU-19 after intravenous administration 20 min, Figure S7. Representative chromatogram of the determination of CU-21 after intravenous administration 5 min, Figure S8. Representative chromatogram of the determination of CU-21 after intravenous administration 10 min, Figure S9. Representative chromatogram of the determination of CU-21 after intravenous administration 20 min, Spectrums of compounds 2–11.
