# Supplementary material for: Synthesis and Anti-Neuroinflammatory Activity of 1,7-diphenyl-1,4-heptadien-3-ones in LPS-Stimulated BV2 Microglia Via Inhibiting NF-κB/MAPK Signaling Pathways
Source: Molecules. 2022 May 31;27(11):3537. doi: 10.3390/molecules27113537 (PMC9181915; doi:10.3390/molecules27113537)
Supplement: Supplementary file 1 [file molecules-27-03537-s001.zip › molecules-1724775-supplementary.pdf]

Supplementary Materials

Synthesis and anti-neuroinflammatory activity of 1,7-diphenyl-1,4-heptadien-3-ones in LPS-stimulated BV2 microglia *via* inhibiting NF-κB/MAPK signaling pathways

Xuan Zhao [a], Jiqing Fang [b], Yu Jia [a], Zi Wu [b], Meihui Zhang [a], Mingyu Xia [b, *] and Jinhua Dong [a, *]

[a] Key Laboratory of Structure-Based Drug Design & Discovery, Ministry of Education, Shenyang Pharmaceutical University, 103 Wenhua Road, Shenhe District, Shenyang City, Liaoning Province, 110016, P.R. China
[b] School of Life Science and Biopharmaceutics, Shenyang Pharmaceutical University, 103 Wenhua Road, Shenhe District, Shenyang City, Liaoning Province, 110016, P.R. China

* Correspondence: author: E-mail: xmywd@vip.sina.com (M. Y. Xia); dongjh66@hotmail.com (J. H. Dong)

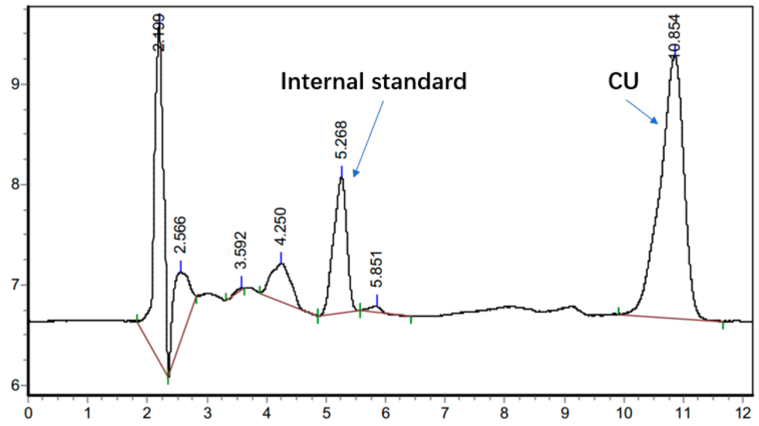

Figure S1 Representative chromatogram of the determination of CU after intravenous administration 5 min.

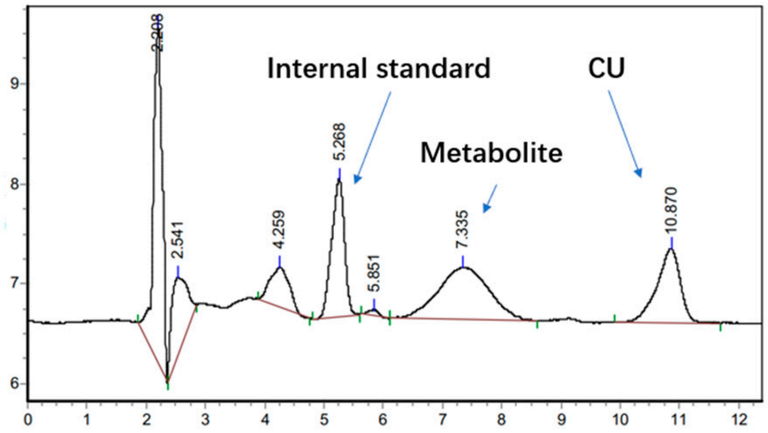

Figure S2 Representative chromatogram of the determination of CU after intravenous administration 10 min.

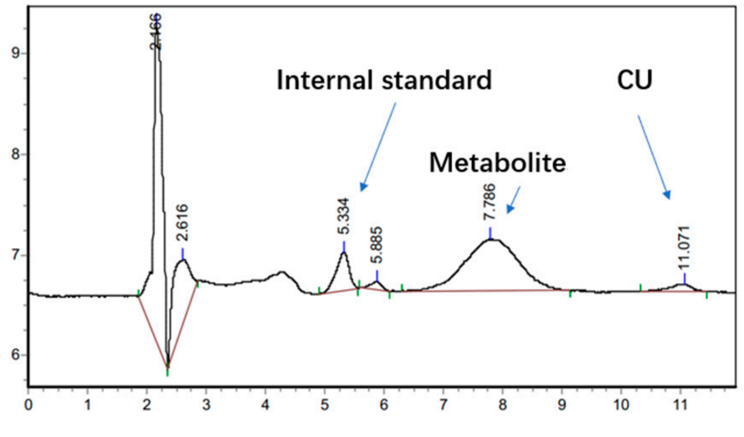

Figure S3 Representative chromatogram of the determination of CU after intravenous administration 20 min.

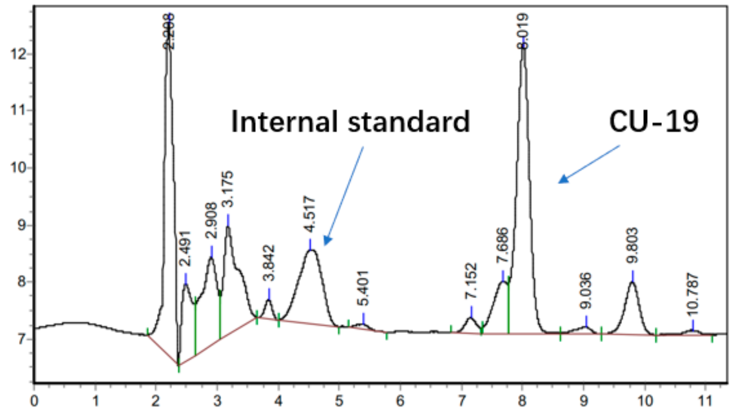

Figure S4 Representative chromatogram of the determination of CU-19 after intravenous administration 5 min.

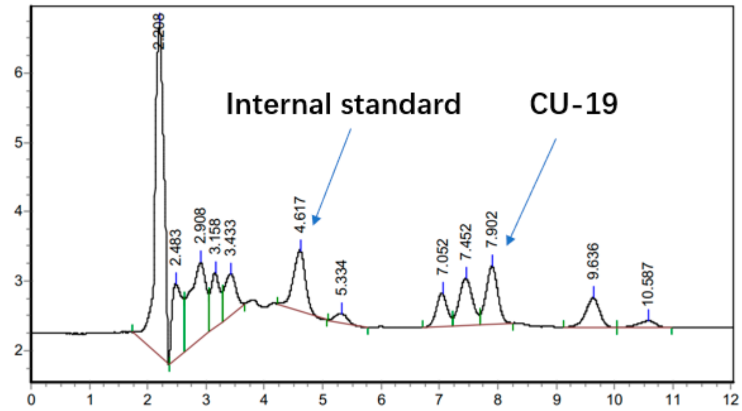

Figure S5 Representative chromatogram of the determination of CU-19 after intravenous administration 10 min.

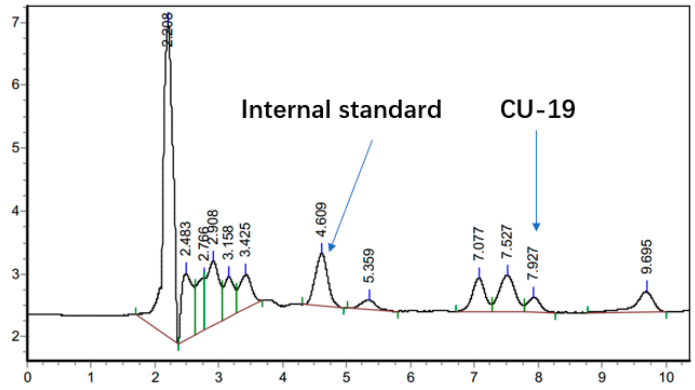

Figure S6 Representative chromatogram of the determination of CU-19 after intravenous administration 20 min.

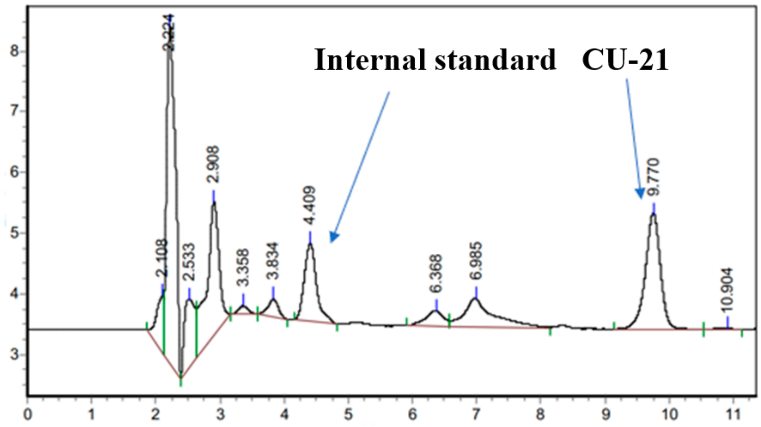

Figure S7 Representative chromatogram of the determination of CU-21 after intravenous administration 5 min.

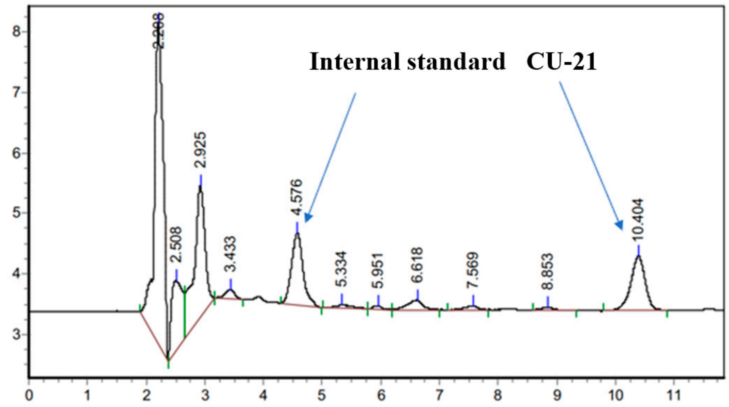

Figure S8 Representative chromatogram of the determination of CU-21 after intravenous administration 10 min.

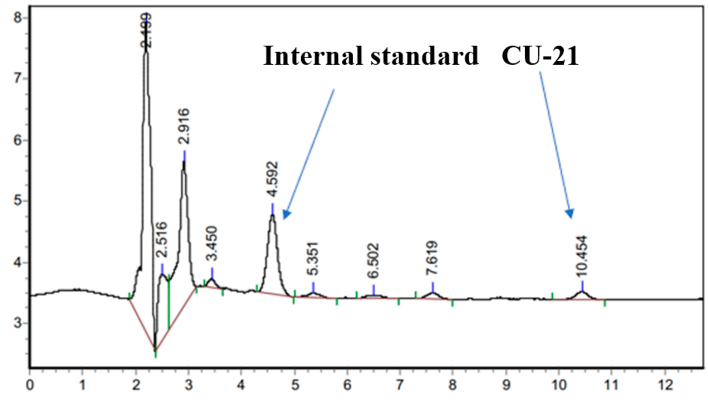

Figure S9 Representative chromatogram of the determination of CU-21 after intravenous administration 20 min.

Spectrums of compounds 2~11

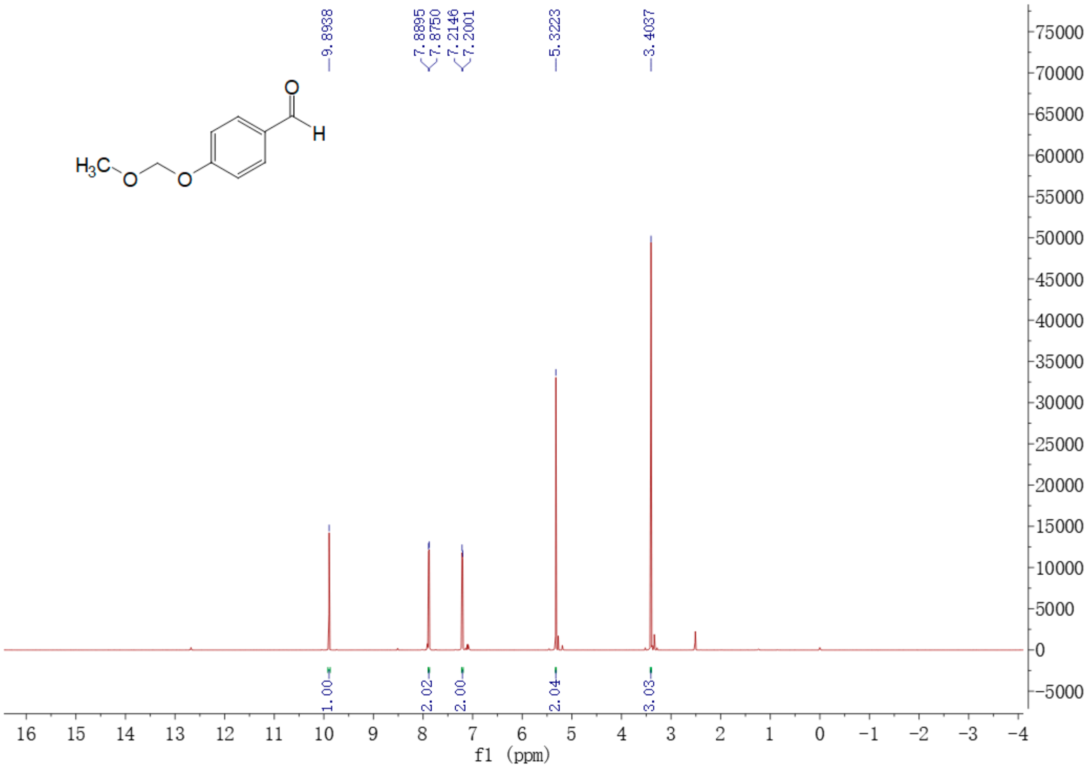

^{1}H NMR spectrum of **2** (600 MHz, DMSO-d_6)

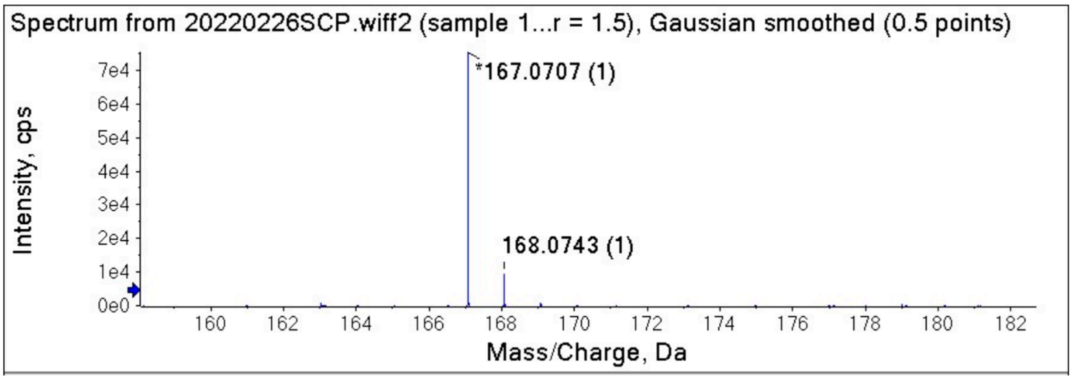

HRMS spectrum of **2**

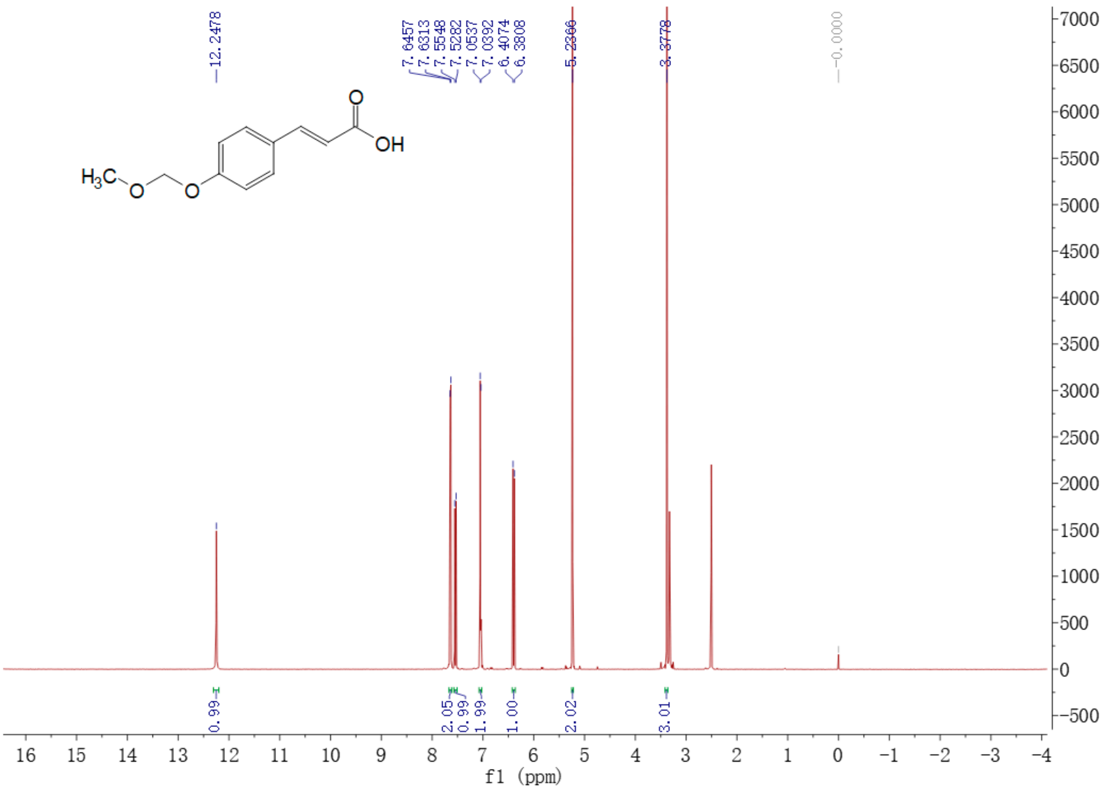

^{1}H NMR spectrum of **3** (600 MHz, DMSO-d_6)

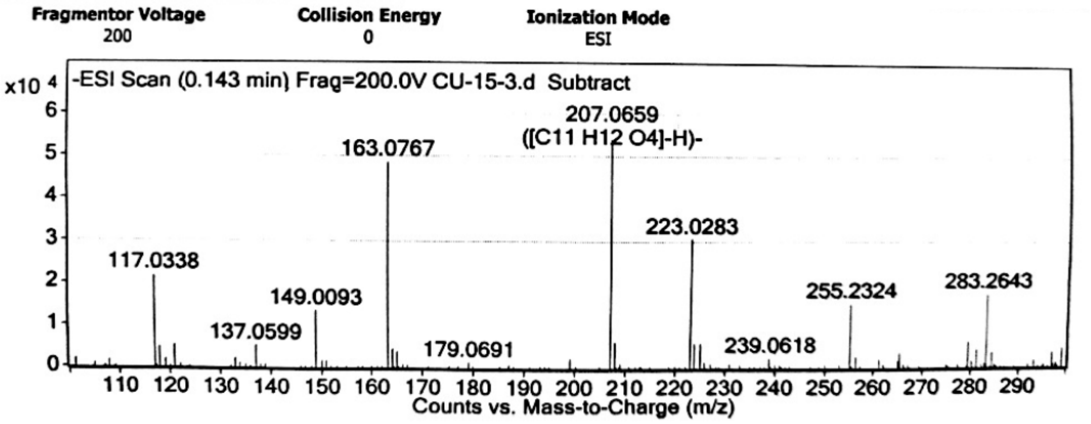

HRMS spectrum of **3**

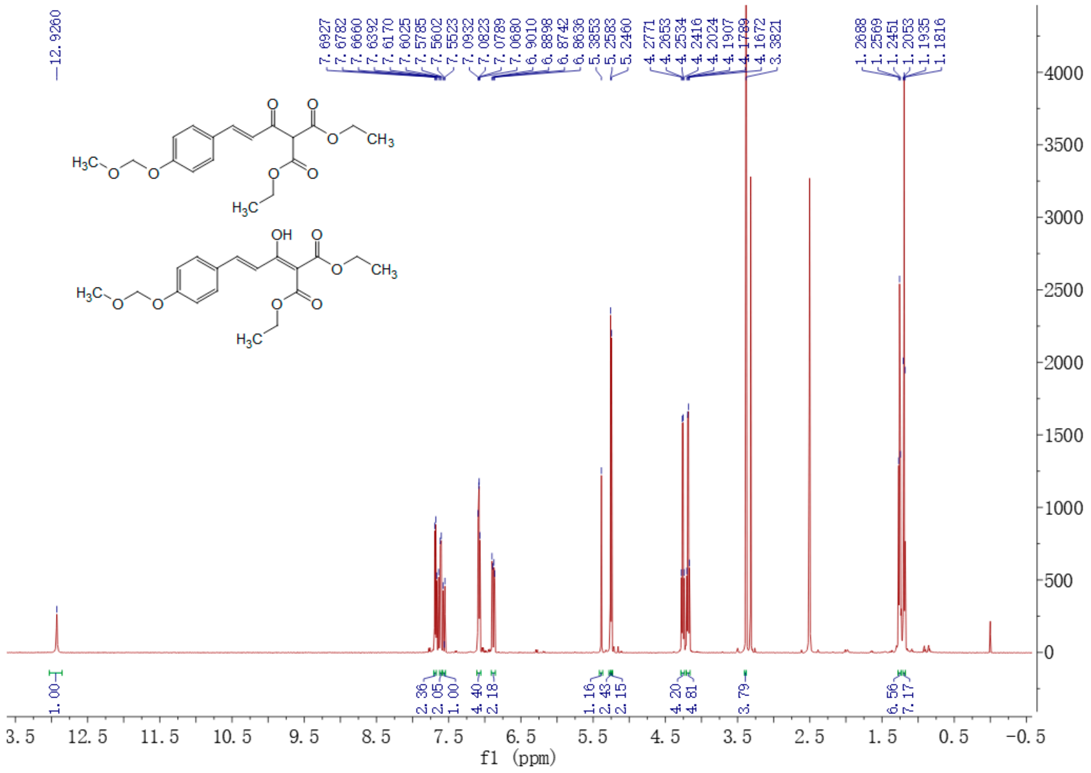

^{1}H NMR spectrum of **4** and **4’** (600 MHz, DMSO-d_6)

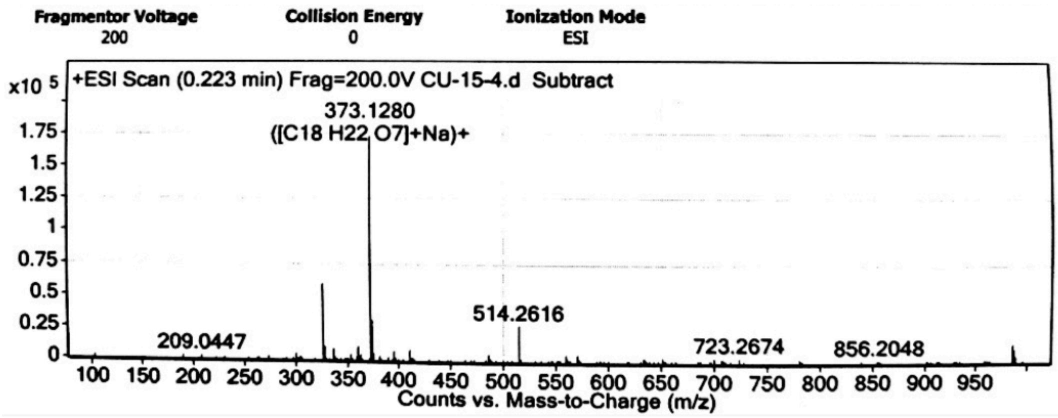

HRMS spectrum of **4** and **4’**

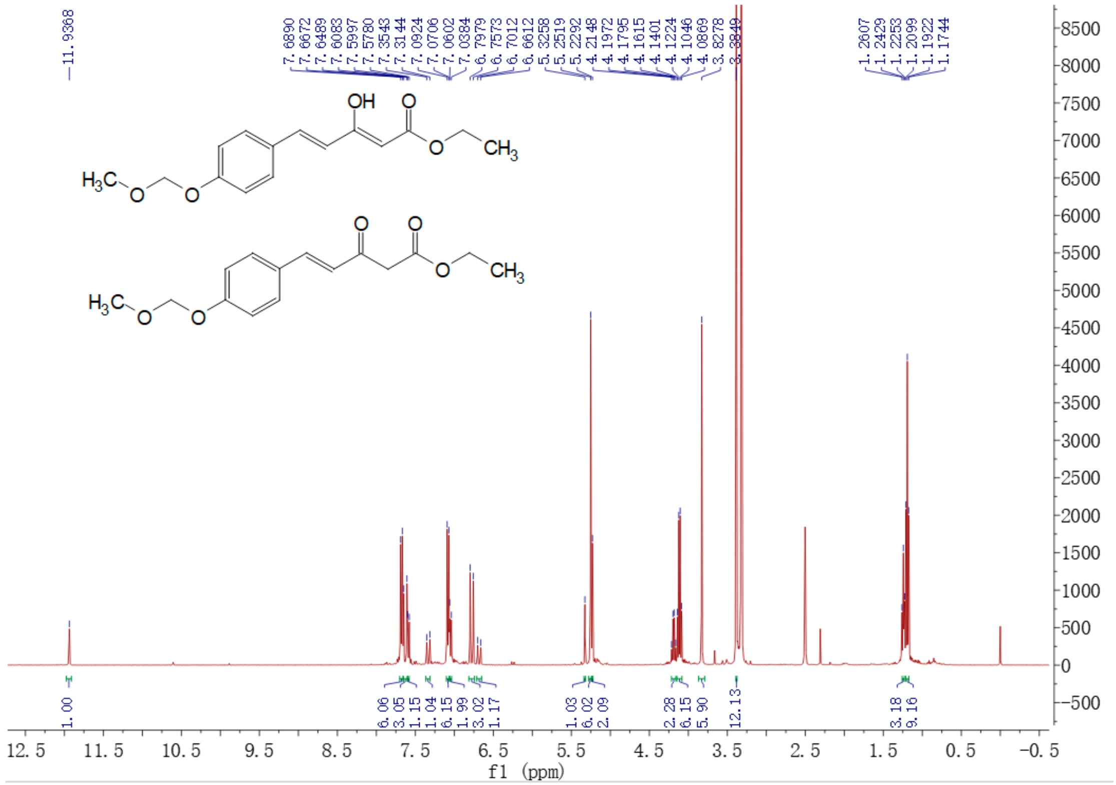

^{1}H NMR spectrum of **5** and **5’** (600 MHz, DMSO-d_6)

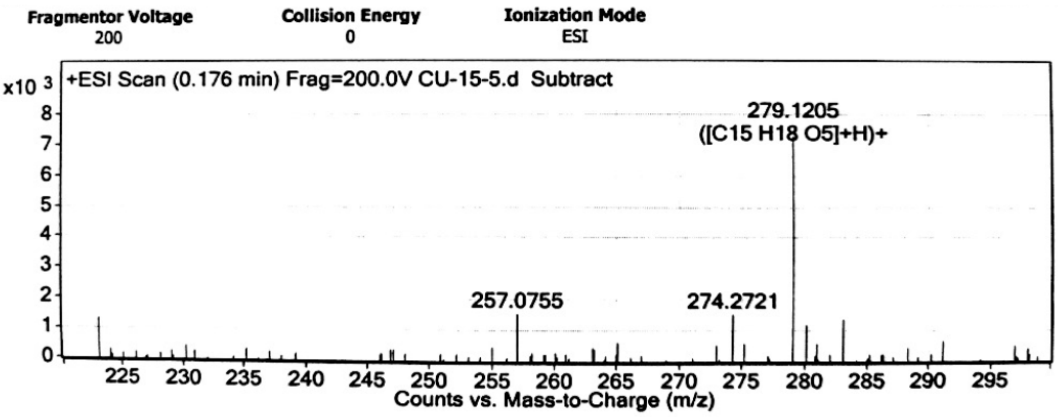

HRMS spectrum of **5** and **5’**

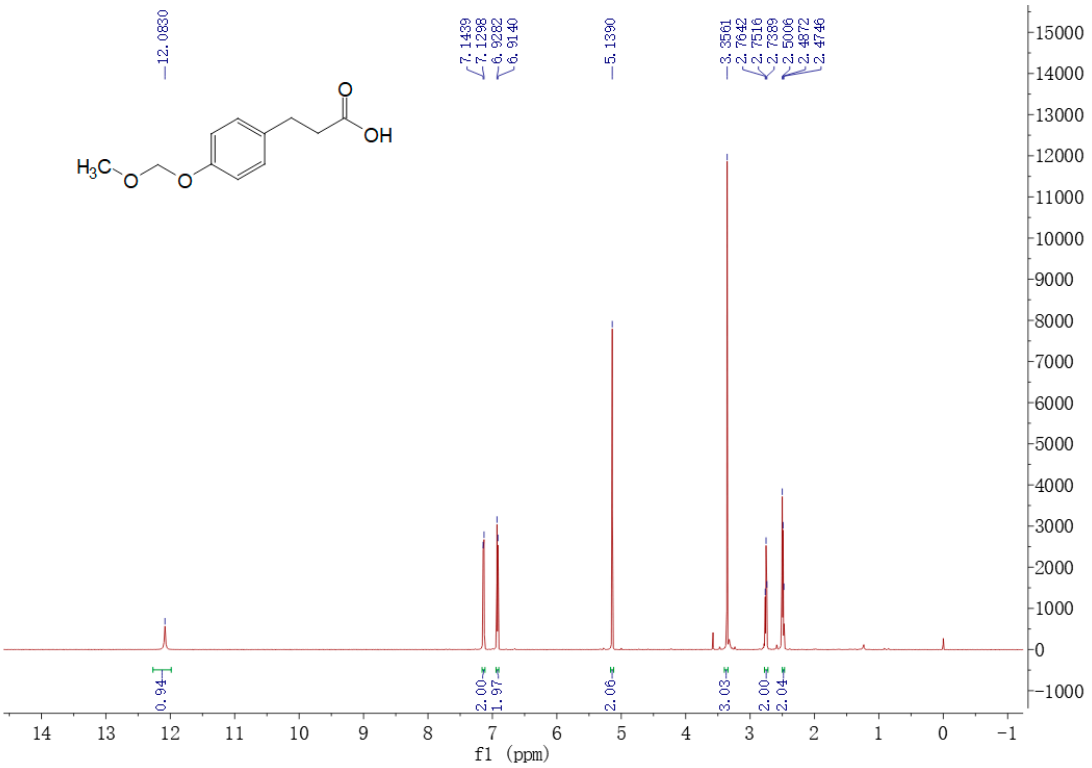

^{1}H NMR spectrum of **6** (600 MHz, DMSO-d_6)

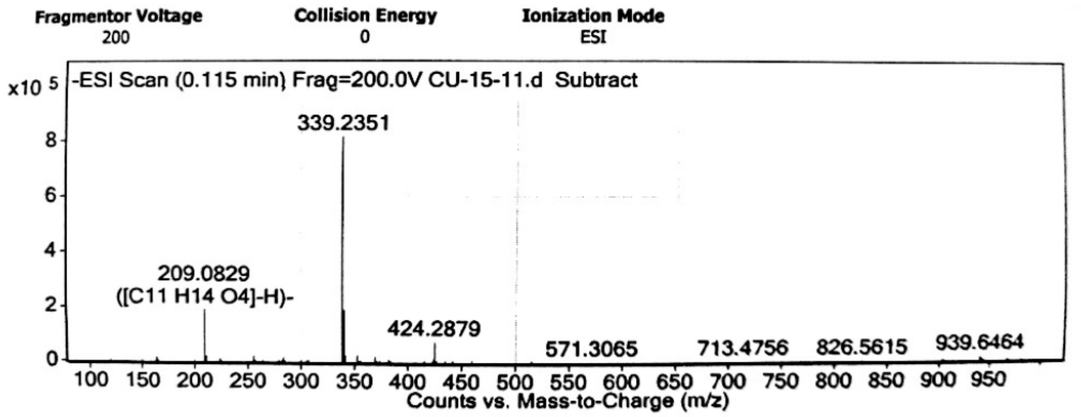

HRMS spectrum of **6**

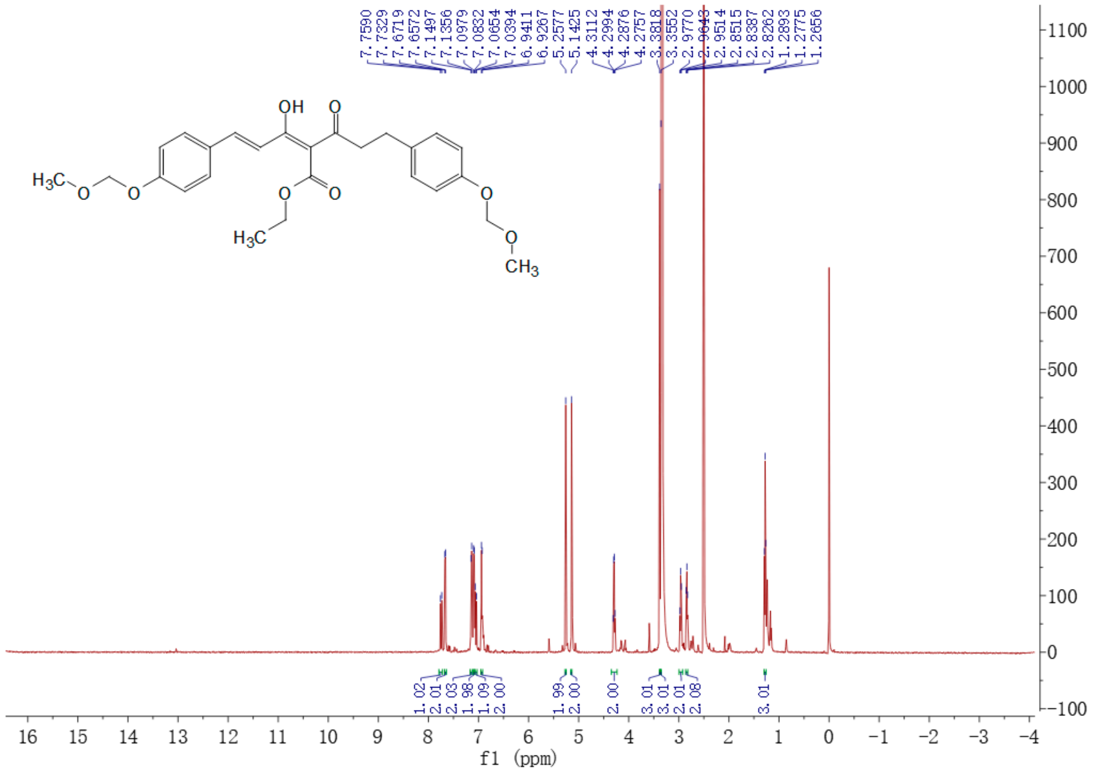

^{1}H NMR spectrum of **8** (600 MHz, DMSO-d_6)

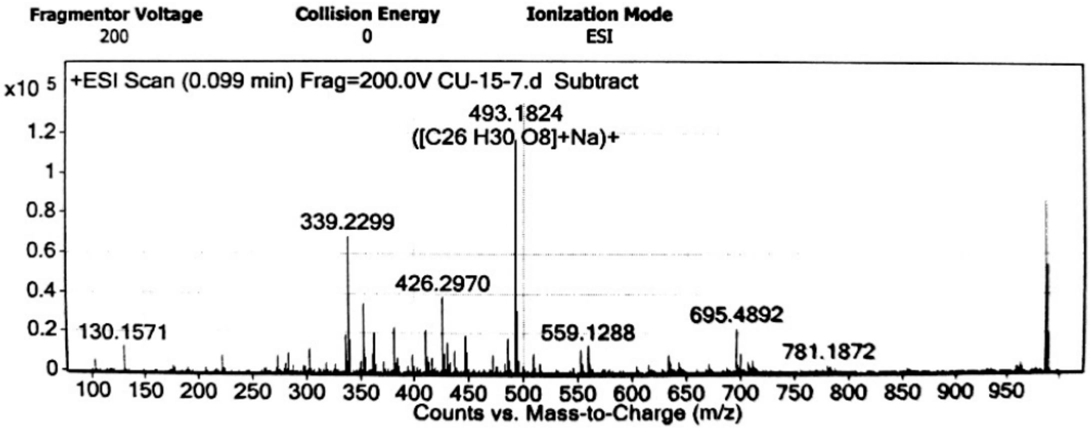

HRMS spectrum of **8**

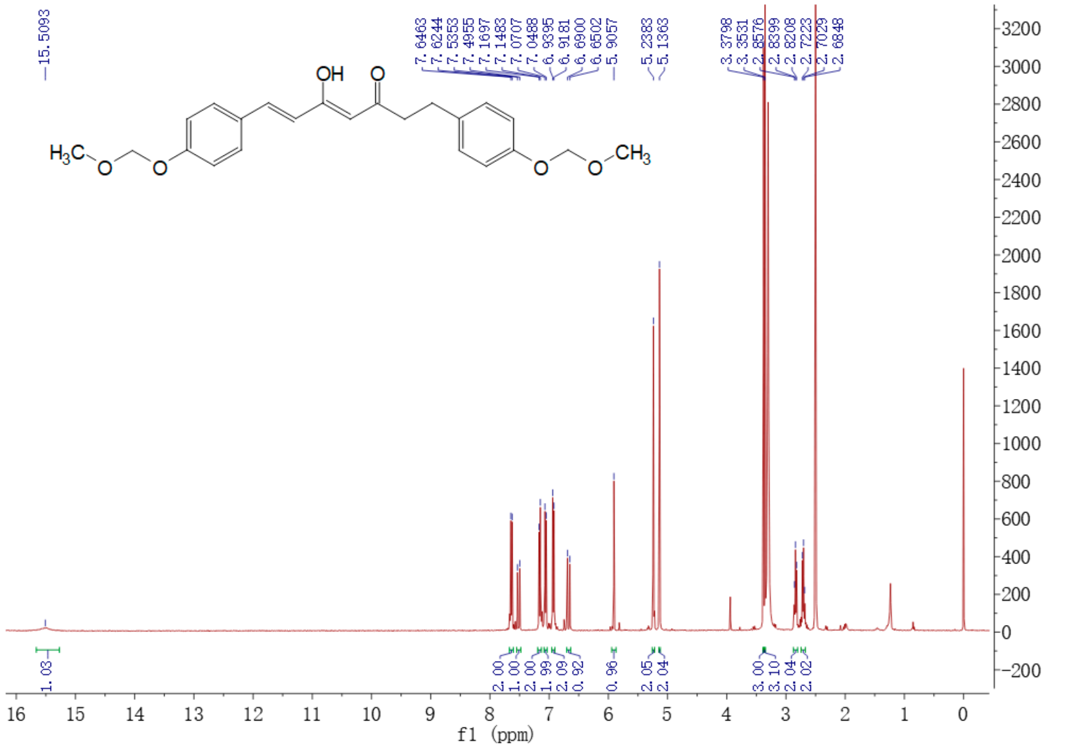

^{1}H NMR spectrum of **9** (600 MHz, DMSO-d_6)

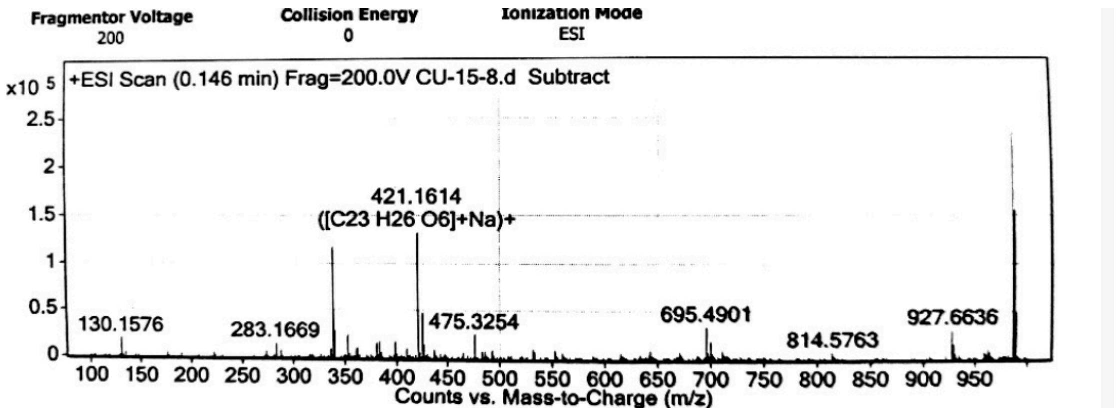

HRMS spectrum of **9**

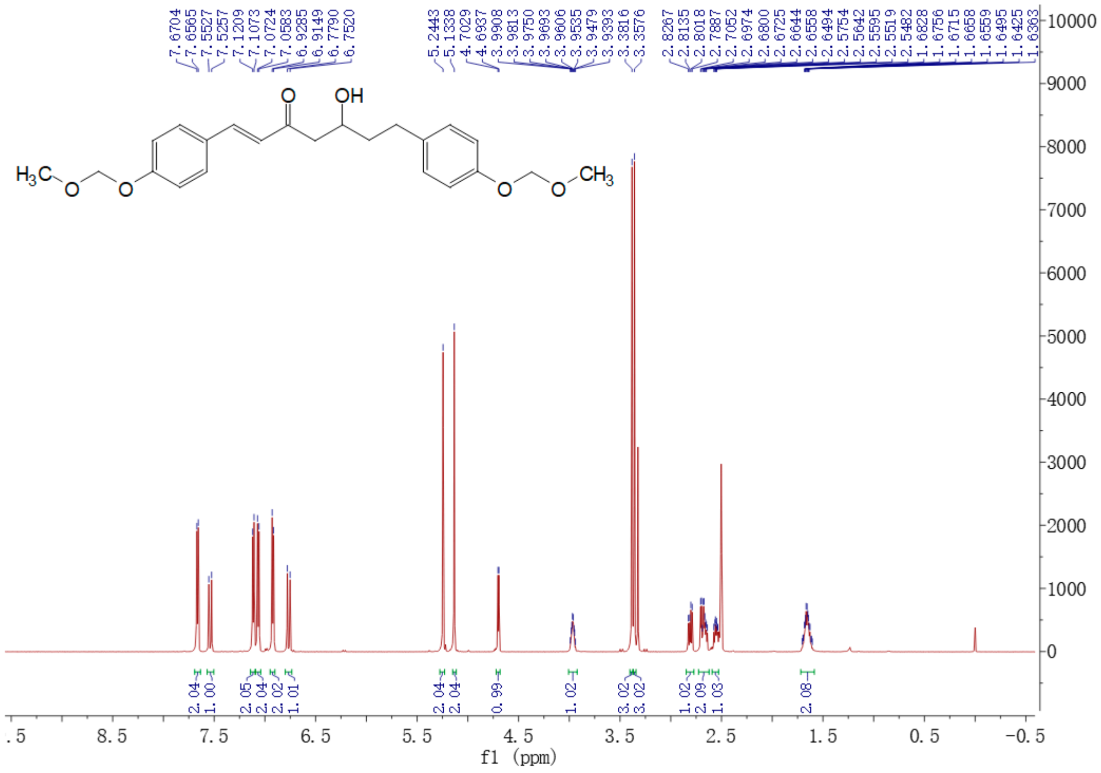

^{1}H NMR spectrum of **10** (600 MHz, DMSO-d_6)

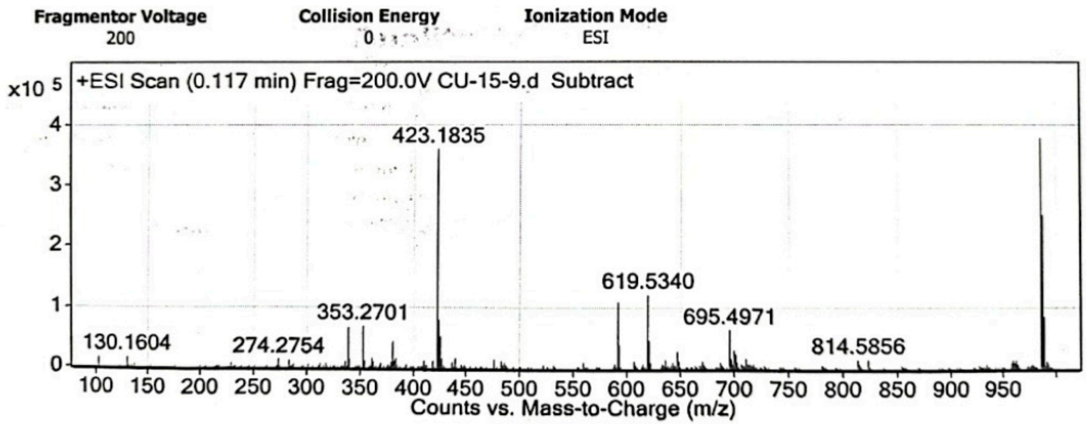

HRMS spectrum of **10**

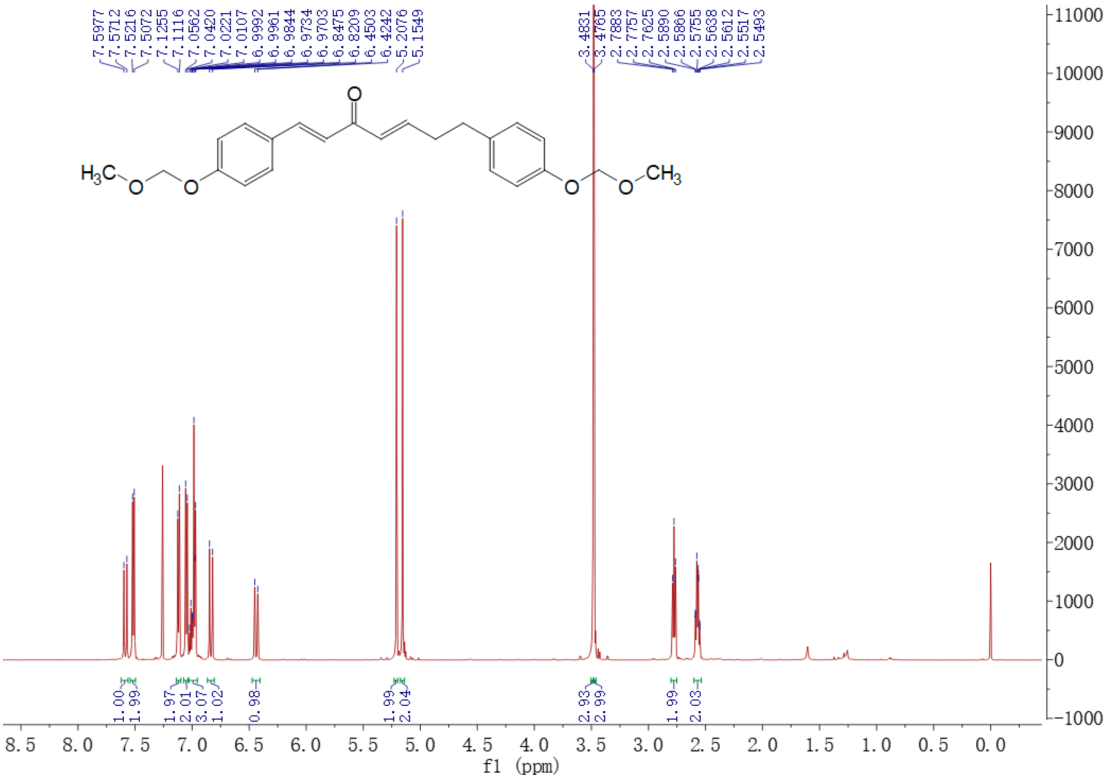

^{1}H NMR spectrum of **11** (600 MHz, $CDCl_3$)

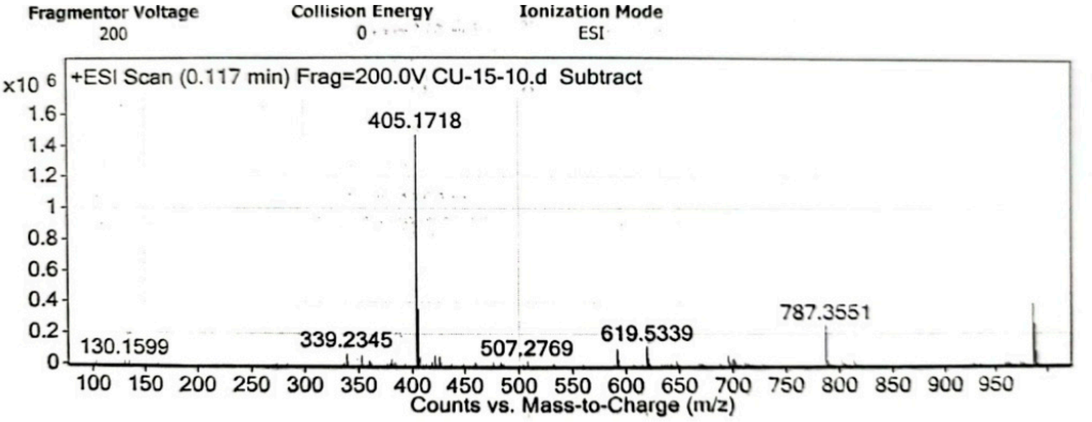

HRMS spectrum of **11**

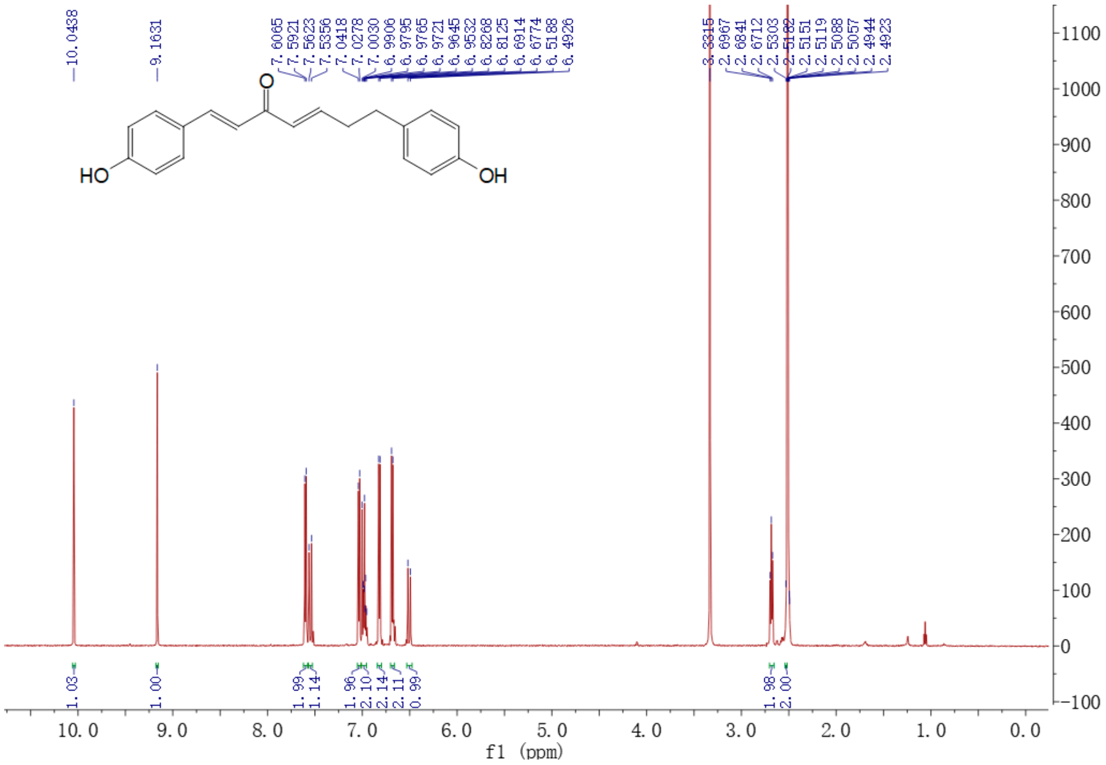

^{1}H NMR spectrum of **CU-1** (600 MHz, DMSO-d_6)

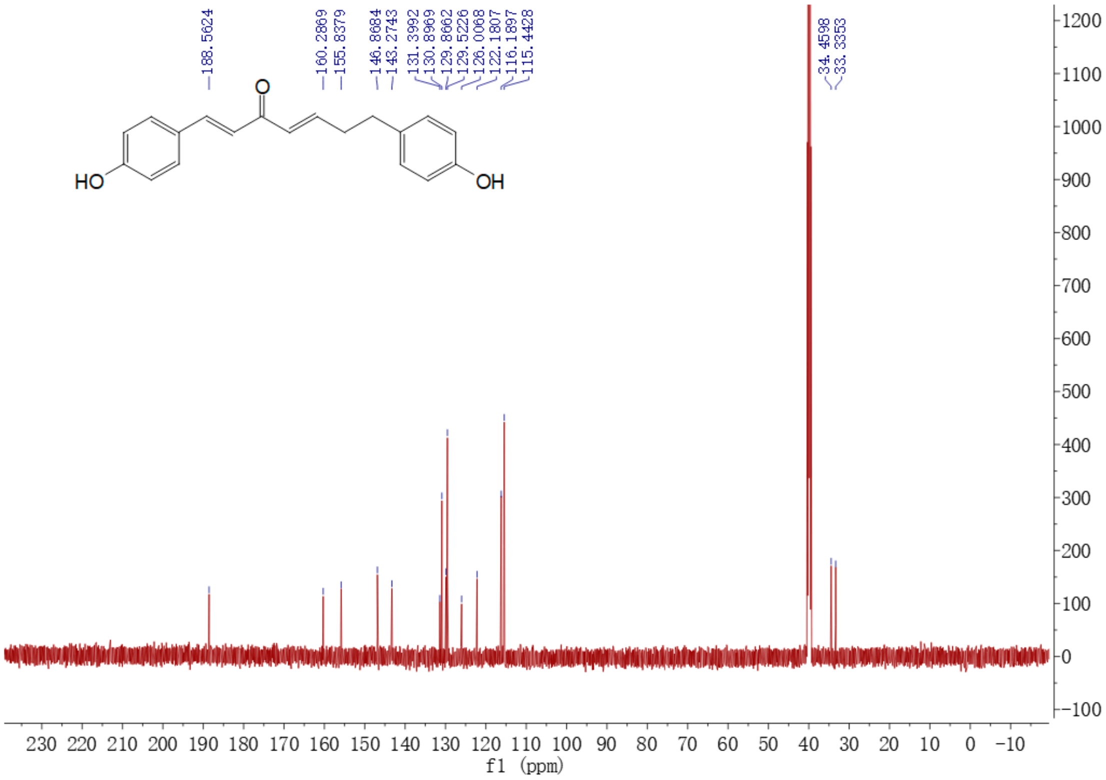

^{13}C NMR spectrum of **CU-1** (151 MHz, DMSO-d_6)

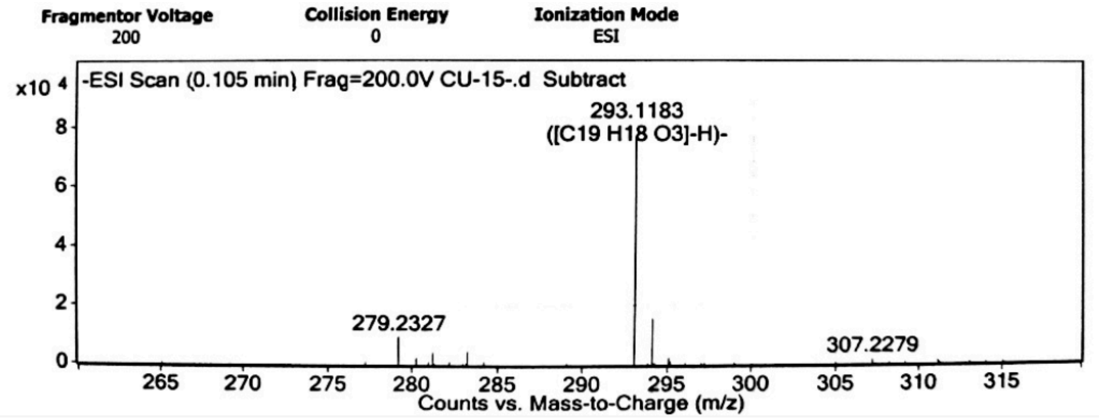

HRMS spectrum of **CU-1**

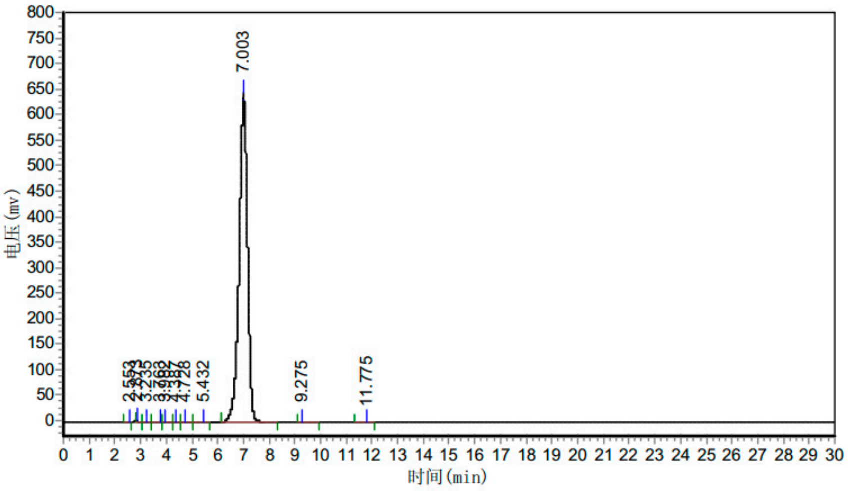

分析结果表

峰号	峰名	保留时间	峰高	峰面积	含量
1		2. 553	1548. 185	18139. 182	0. 0980
2		2. 873	3101. 354	19163. 109	0. 1036
3		3. 235	906. 018	17409. 188	0. 0941
4		3. 763	1168. 499	22575. 072	0. 1220
5		3. 982	1991. 770	35422. 590	0. 1914
6		4. 387	1059. 317	15540. 724	0. 0840
7		4. 728	831. 542	20692. 996	0. 1118
8		5. 432	720. 256	22374. 178	0. 1209
9		7. 003	645418. 750	18320394. 000	99. 0143
10		9. 275	367. 351	9083. 099	0. 0491
11		11. 775	86. 595	1988. 392	0. 0107
**总计**			657199. 638	18502782. 528	100. 0000

The HPLC spectrum of **CU-1**

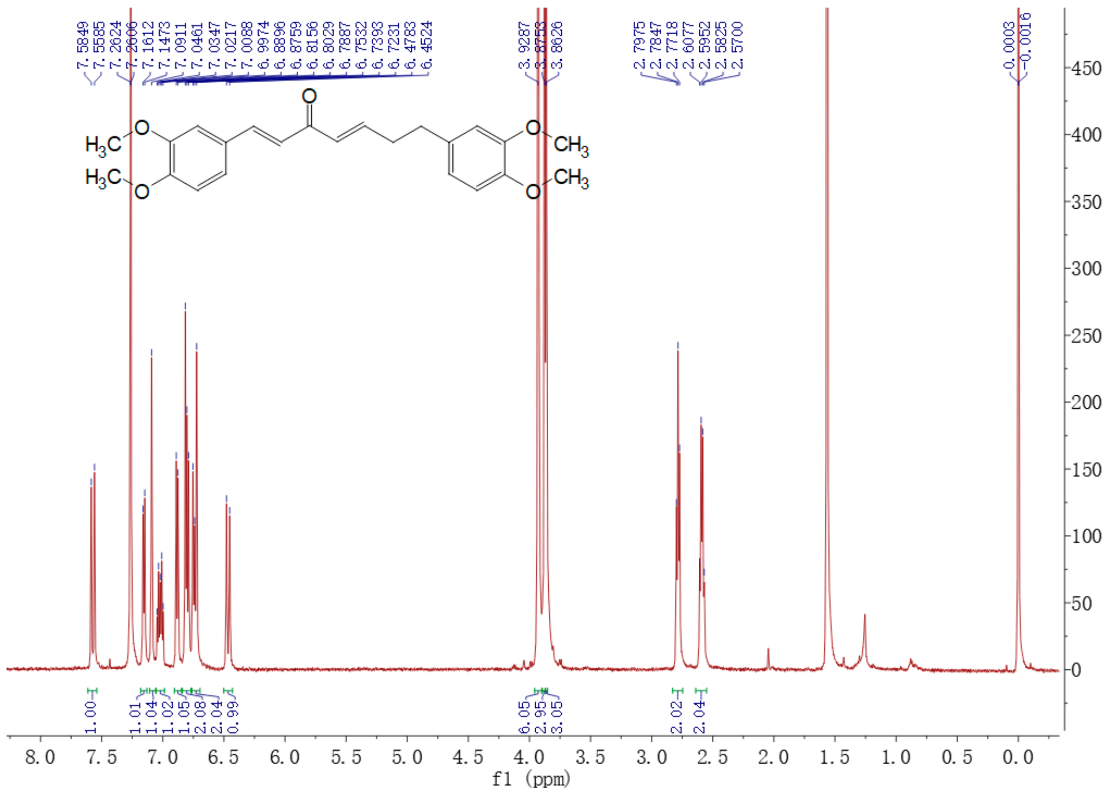

^{1}H NMR spectrum of **CU-2** (600 MHz, $CDCl_3$)

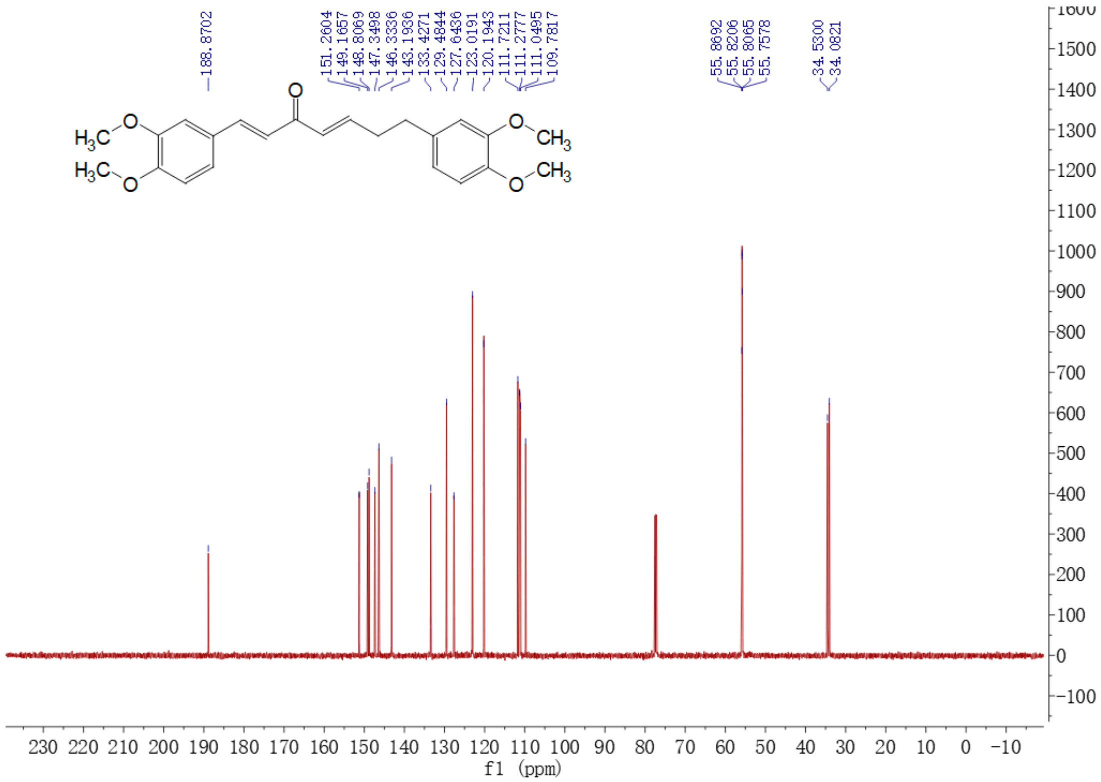

^{13}C NMR spectrum of **CU-2** (151 MHz, $CDCl_3$)

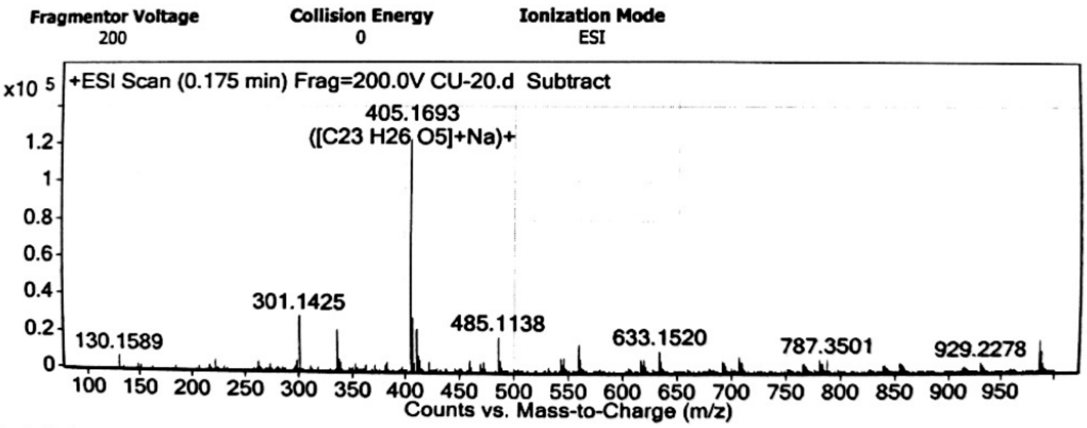

HRMS spectrum of **CU-2**

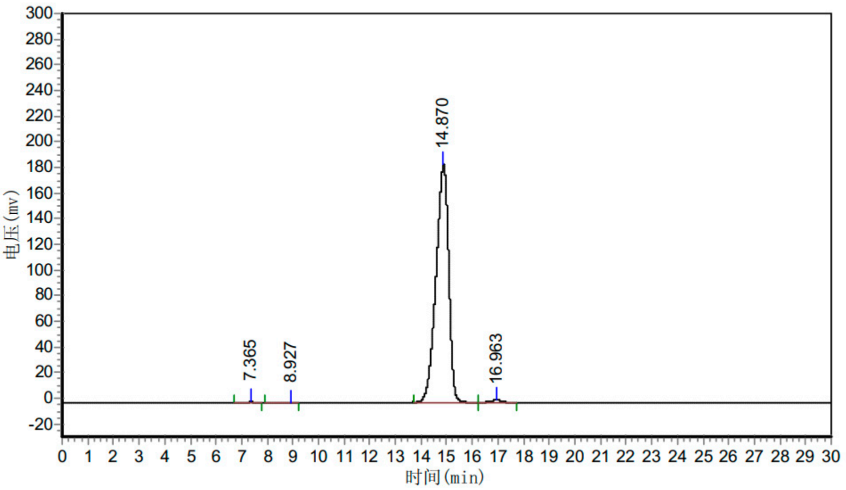

分析结果表

峰号	峰名	保留时间	峰高	峰面积	含量
1		7.365	804.965	22712.398	0.3468
2		8.927	168.721	3926.471	0.0600
3		14.870	186109.031	6434450.000	98.2499
4		16.963	2187.258	87979.414	1.3434
总计			189269.975	6549068.283	100.0000

The HPLC spectrum of **CU-2**

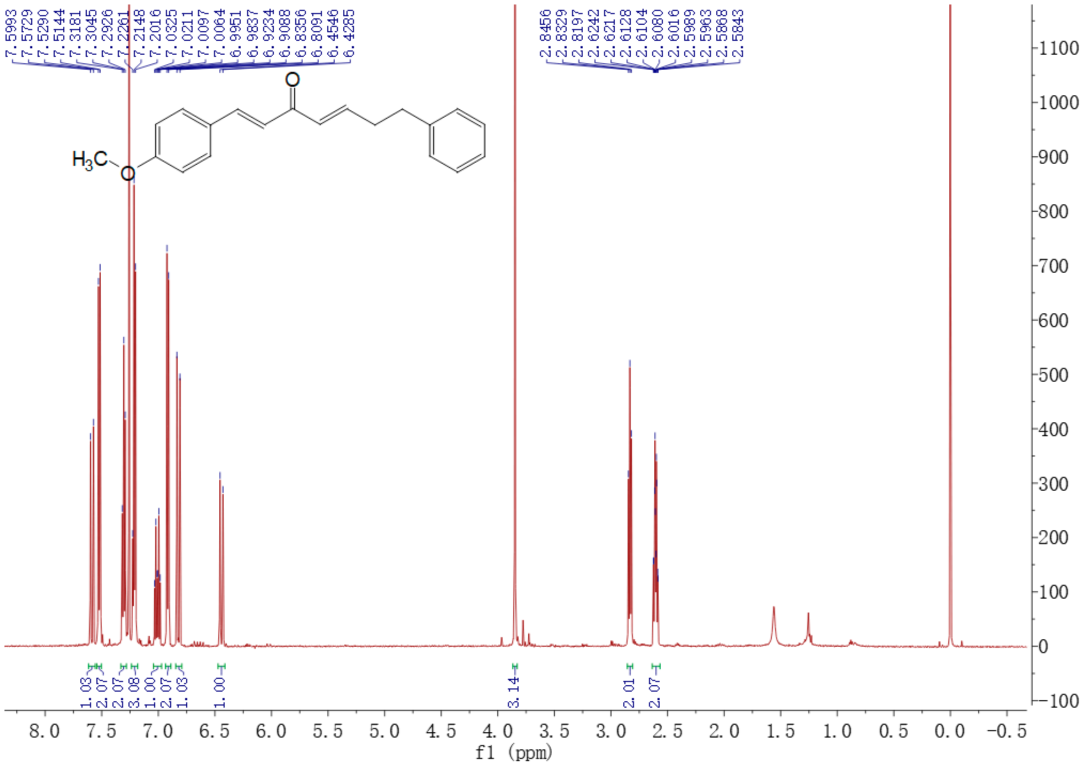

^{1}H NMR spectrum of **CU-3** (600 MHz, $CDCl_3$)

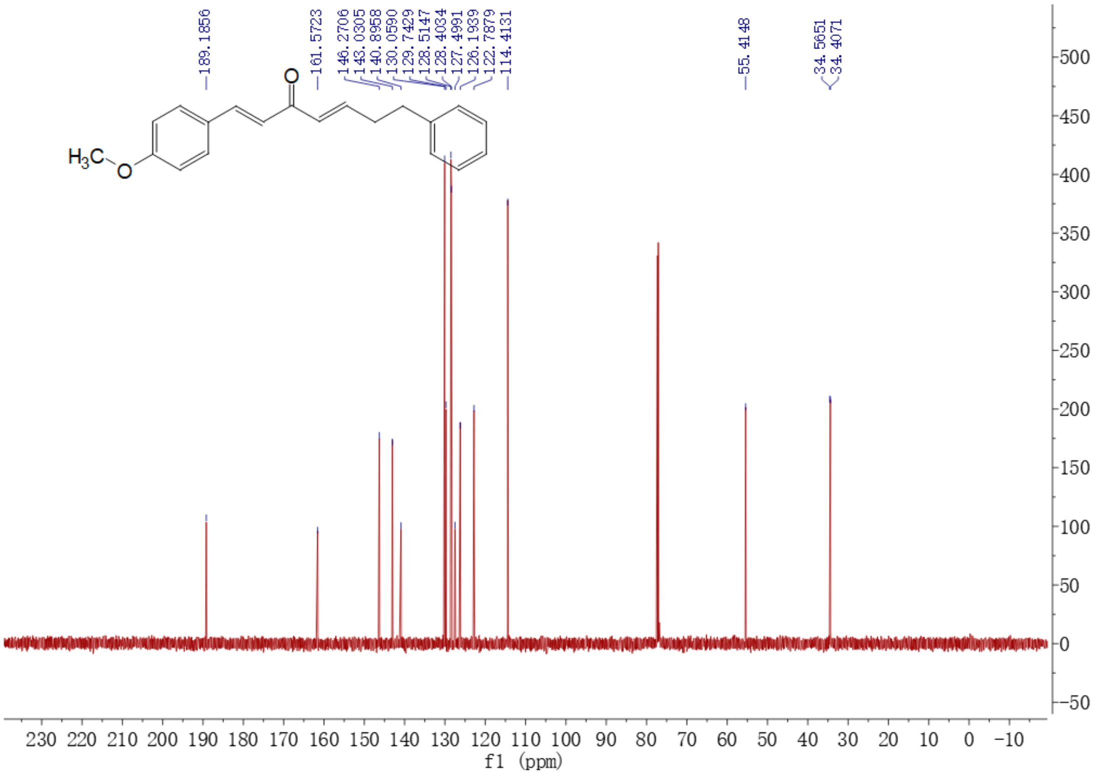

^{13}C NMR spectrum of **CU-3** (151 MHz, $CDCl_3$)

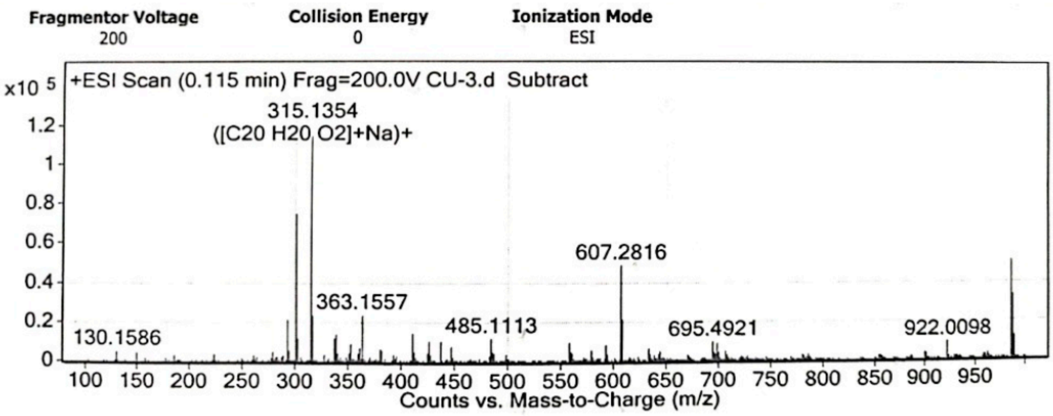

HRMS spectrum of **CU-3**

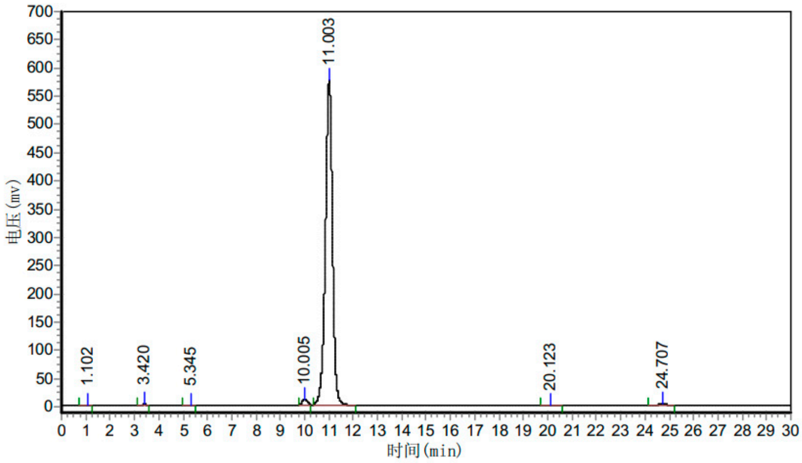

分析结果表

峰号	峰名	保留时间	峰高	峰面积	含量
1		1. 102	203. 081	3744. 752	0. 0311
2		3. 420	1888. 172	20904. 396	0. 1737
3		5. 345	743. 343	17359. 500	0. 1442
4		10. 005	9726. 727	388304. 169	3. 2265
5		11. 003	576958. 688	11518896. 067	95. 7129
6		20. 123	887. 903	28426. 102	0. 2362
7		24. 707	1559. 765	57209. 051	0. 4754
**总计**			591967. 677	12034841. 769	100. 0000

The HPLC spectrum of **CU-3**

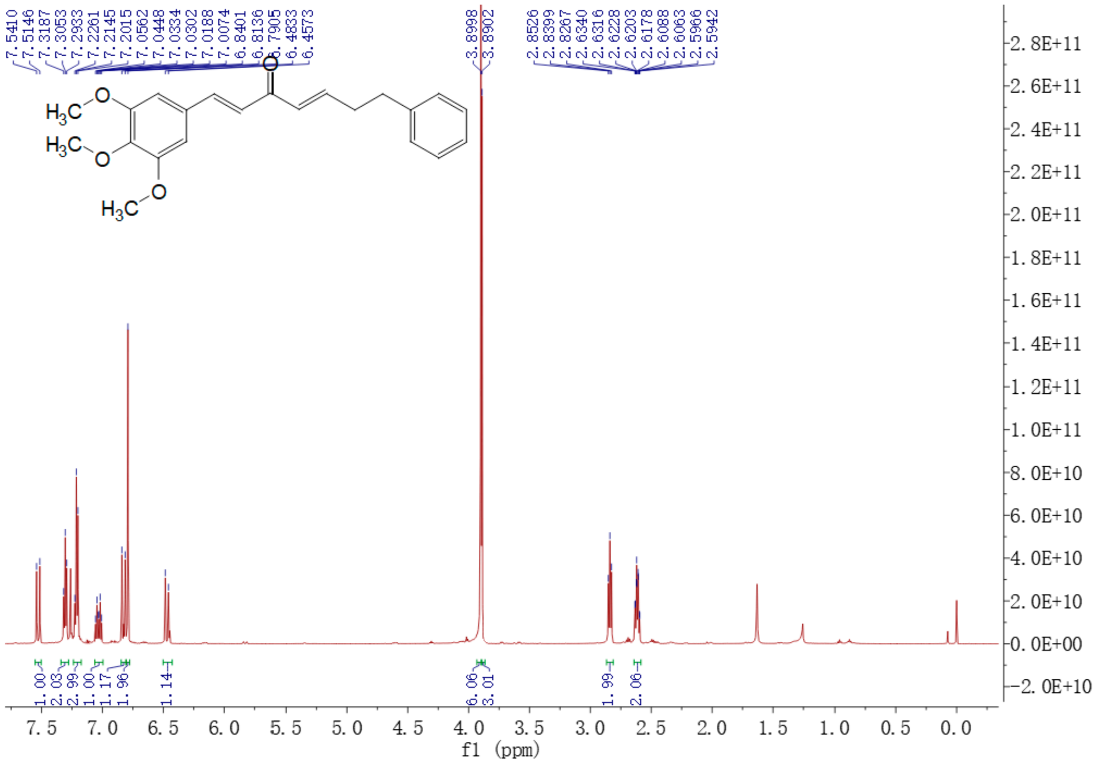

^{1}H NMR spectrum of **CU-4** (600 MHz, $CDCl_3$)

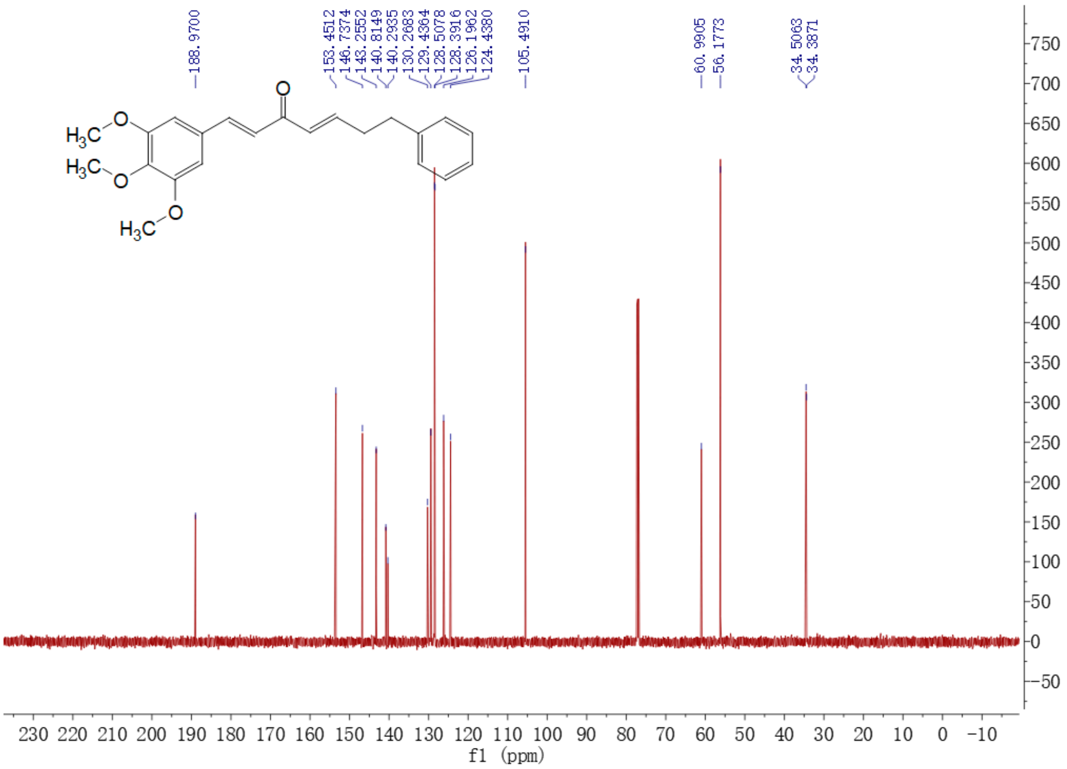

^{13}C NMR spectrum of **CU-4** (151 MHz, $CDCl_3$)

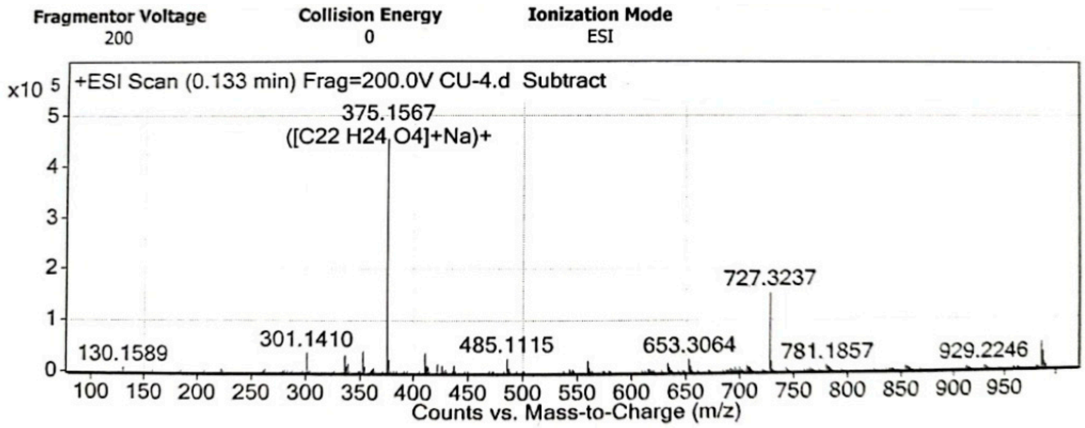

HRMS spectrum of **CU-4**

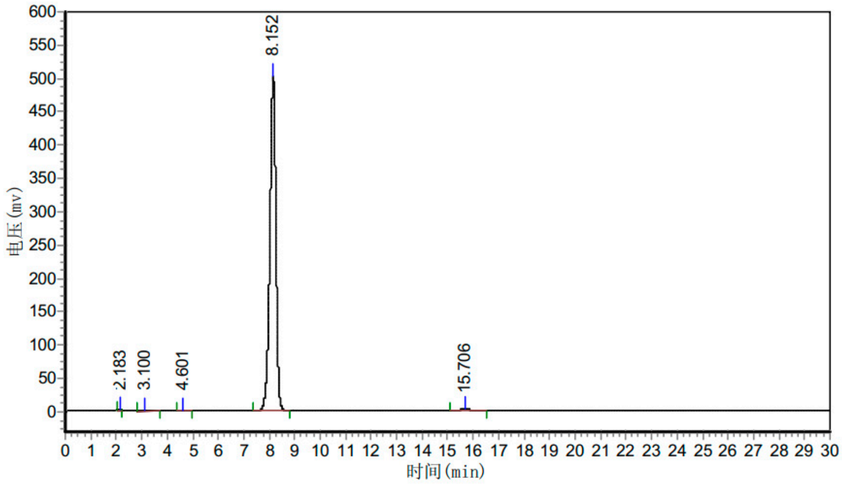

分析结果表

峰号	峰名	保留时间	峰高	峰面积	含量
1		2.183	2660.237	17210.066	0.2034
2		3.100	1213.497	29638.703	0.3503
3		4.601	1762.348	16199.650	0.1915
4		8.152	501713.938	8296078.500	98.0636
5		15.706	2893.486	100767.570	1.1911
总计			510243.505	8459894.490	100.0000

The HPLC spectrum of **CU-4**

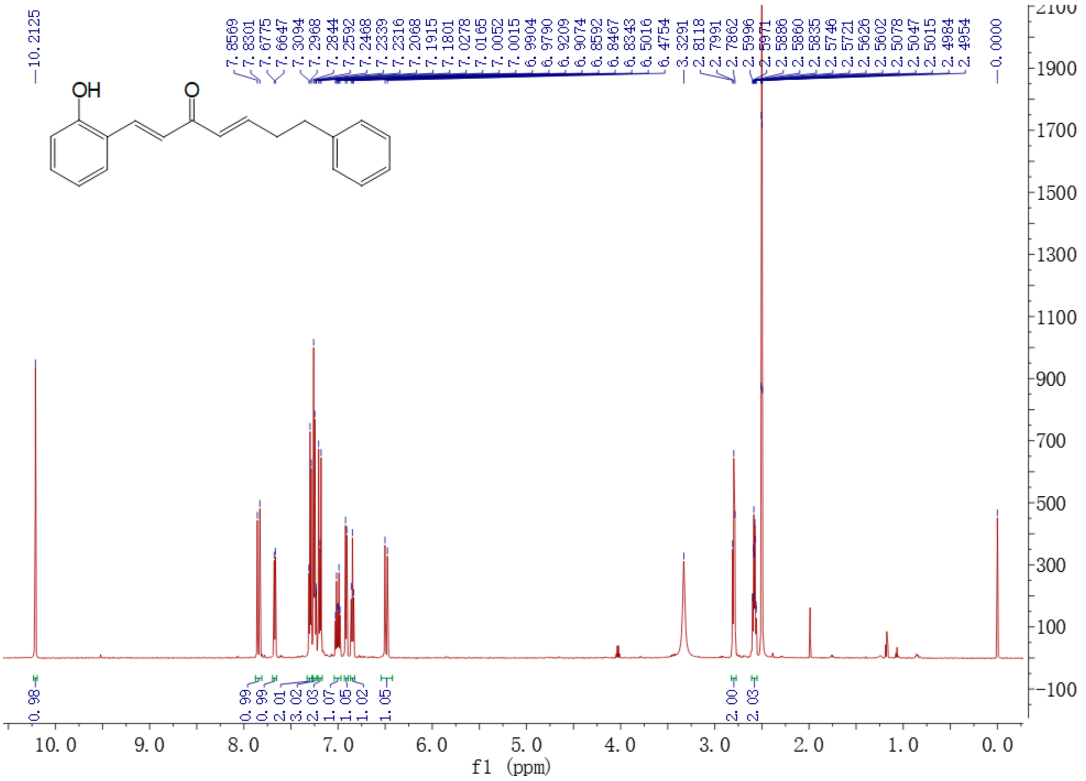

^{1}H NMR spectrum of **CU-5** (600 MHz, DMSO-d_6)

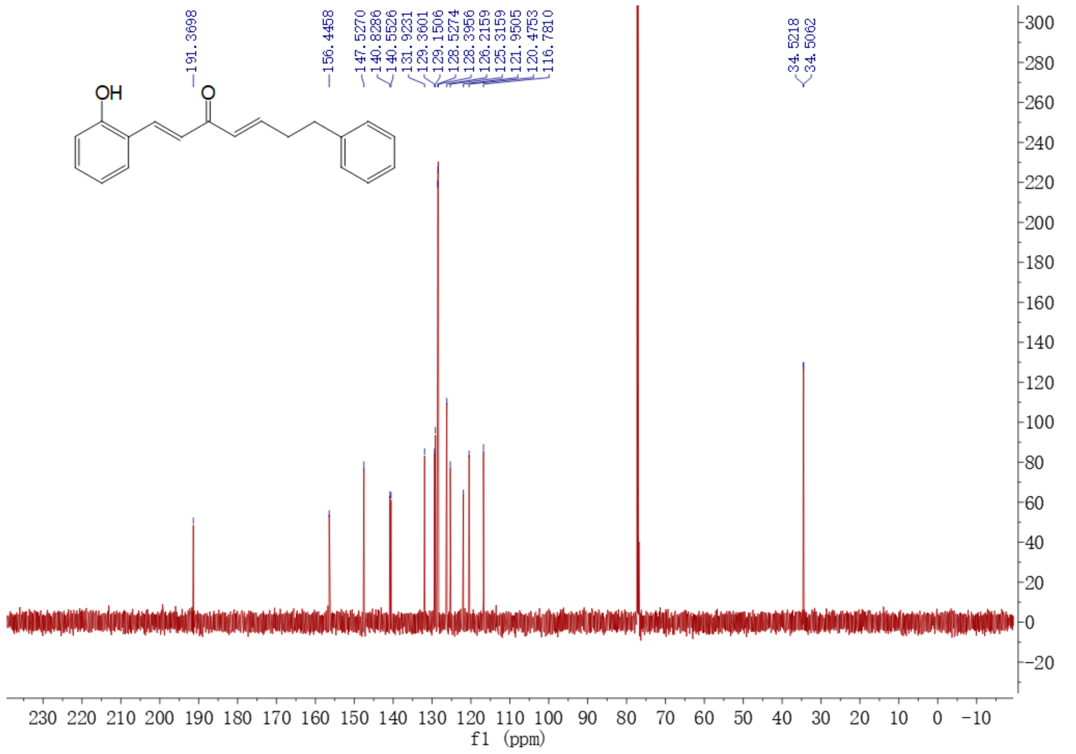

^{13}C NMR spectrum of **CU-5** (151 MHz, $CDCl_3$)

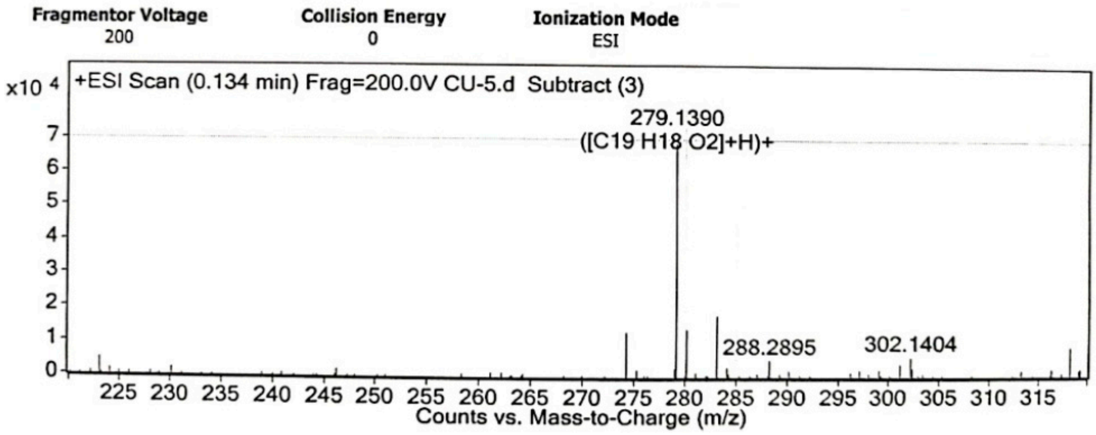

HRMS spectrum of **CU-5**

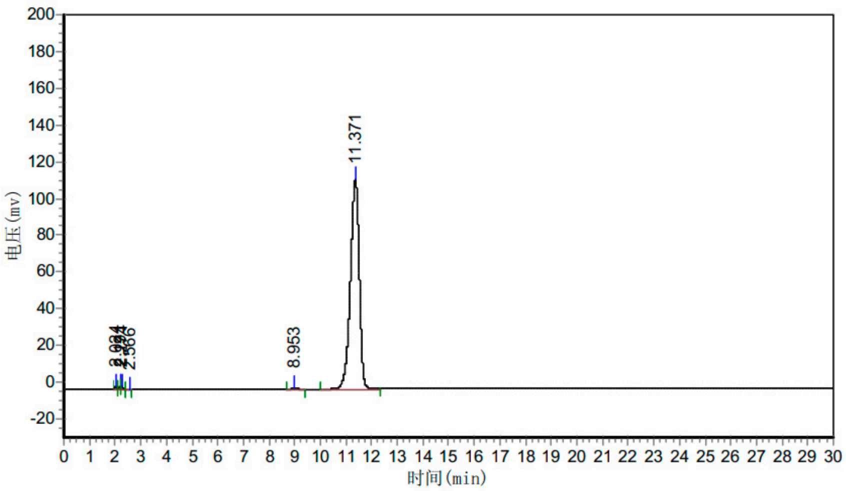

分析结果表

峰号	峰名	保留时间	峰高	峰面积	含量
1		2.024	1564.333	13489.207	0.4843
2		2.191	2114.852	12113.872	0.4349
3		2.274	1850.111	10287.735	0.3693
4		2.566	470.276	4738.137	0.1701
5		8.953	98.844	2490.900	0.0894
6		11.371	113559.117	2742349.000	98.4520
总计			119657.534	2785468.851	100.0000

The HPLC spectrum of **CU-5**

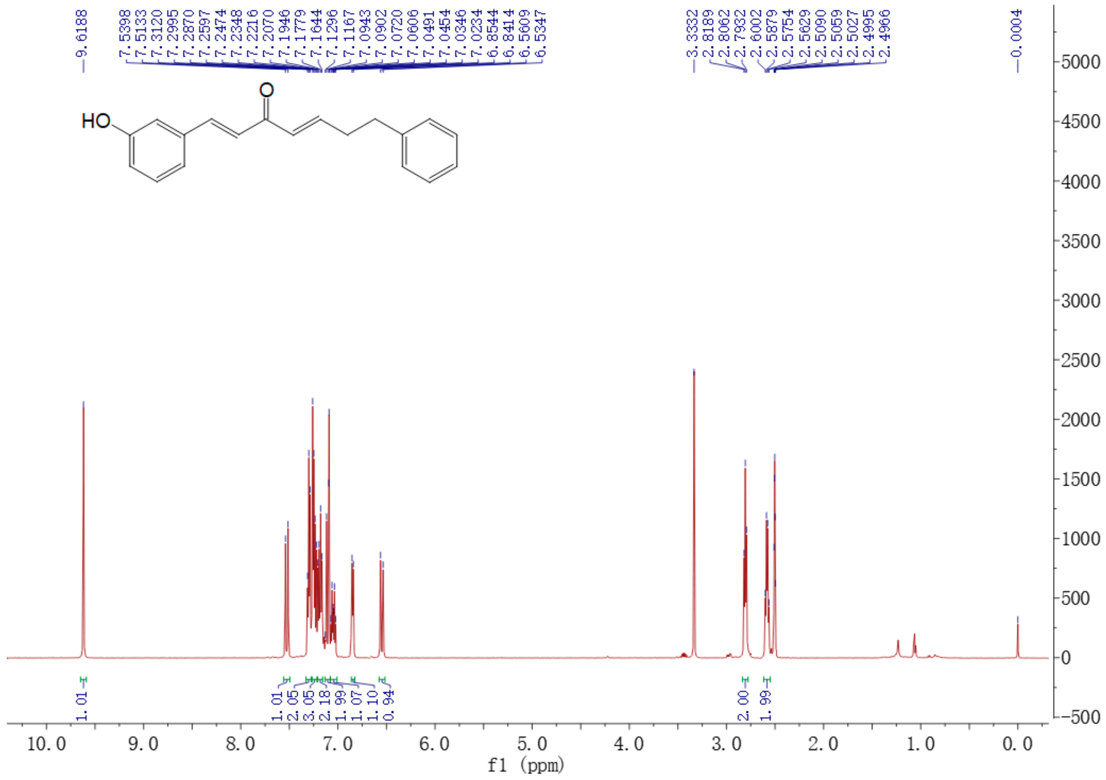

^{1}H NMR spectrum of **CU-6** (600 MHz, DMSO-d_6)

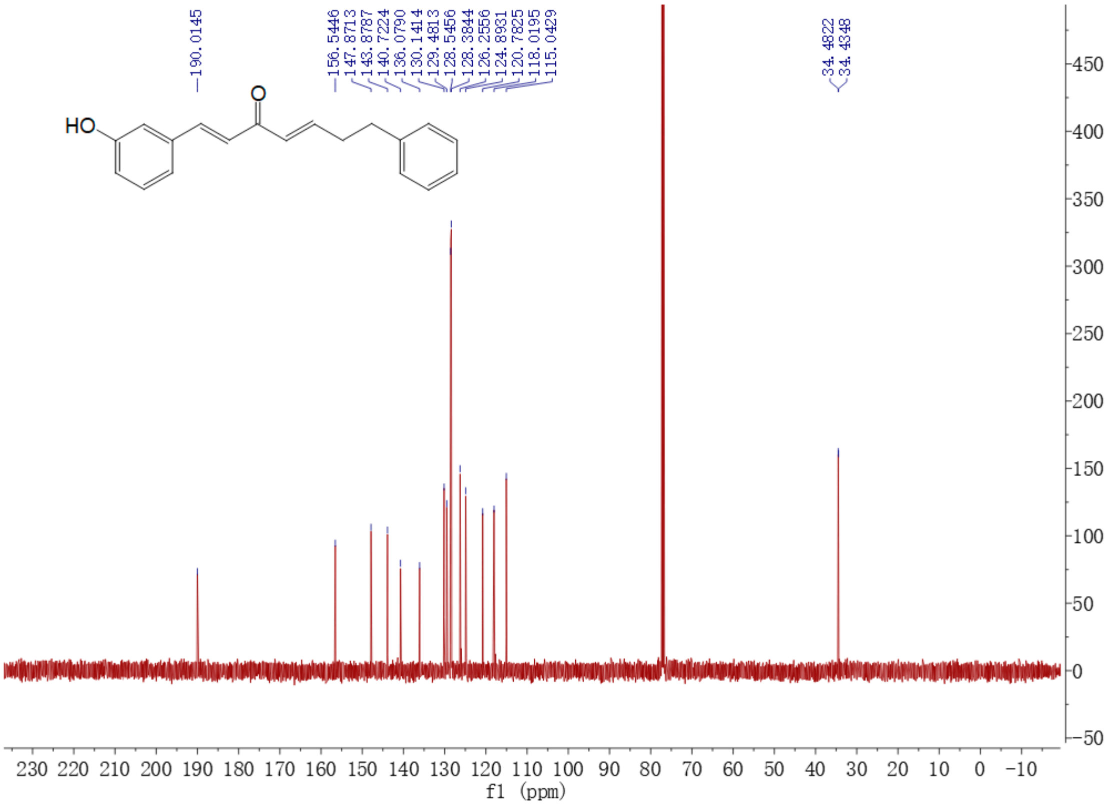

^{13}C NMR spectrum of **CU-6** (151 MHz, $CDCl_3$)

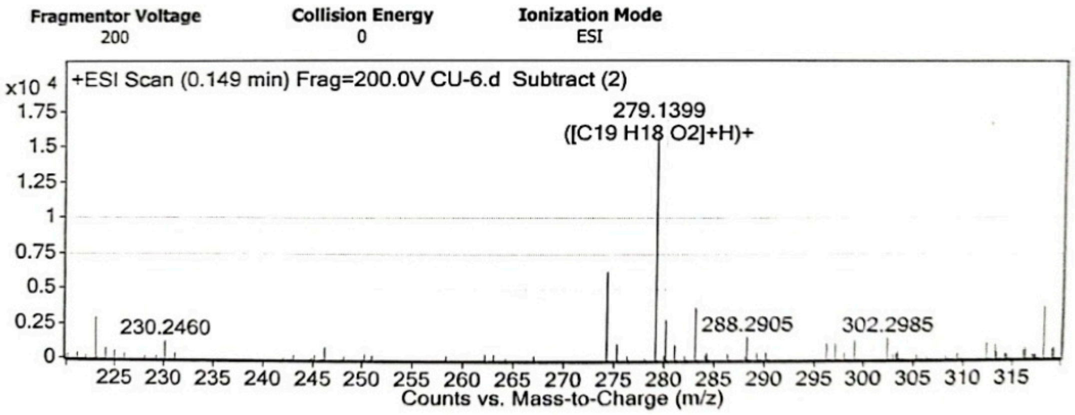

HRMS spectrum of **CU-6**

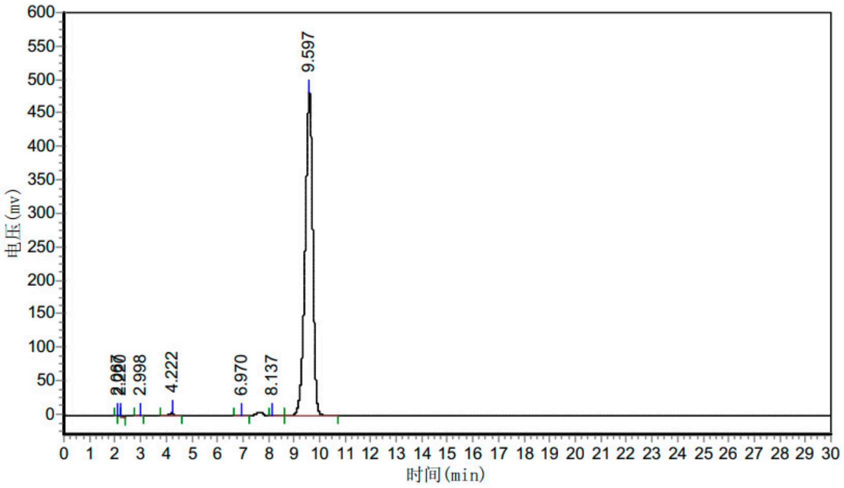

分析结果表

峰号	峰名	保留时间	峰高	峰面积	含量
1		2.067	1474.099	9773.155	0.0955
2		2.220	2127.150	14409.883	0.1408
3		2.998	995.628	13624.840	0.1332
4		4.222	4162.896	63434.148	0.6200
5		6.970	1579.448	35169.191	0.3437
6		8.137	164.289	3126.660	0.0306
7		9.597	484405.719	10091741.000	98.6362
总计			494909.230	10231278.878	100.0000

The HPLC spectrum of **CU-6**

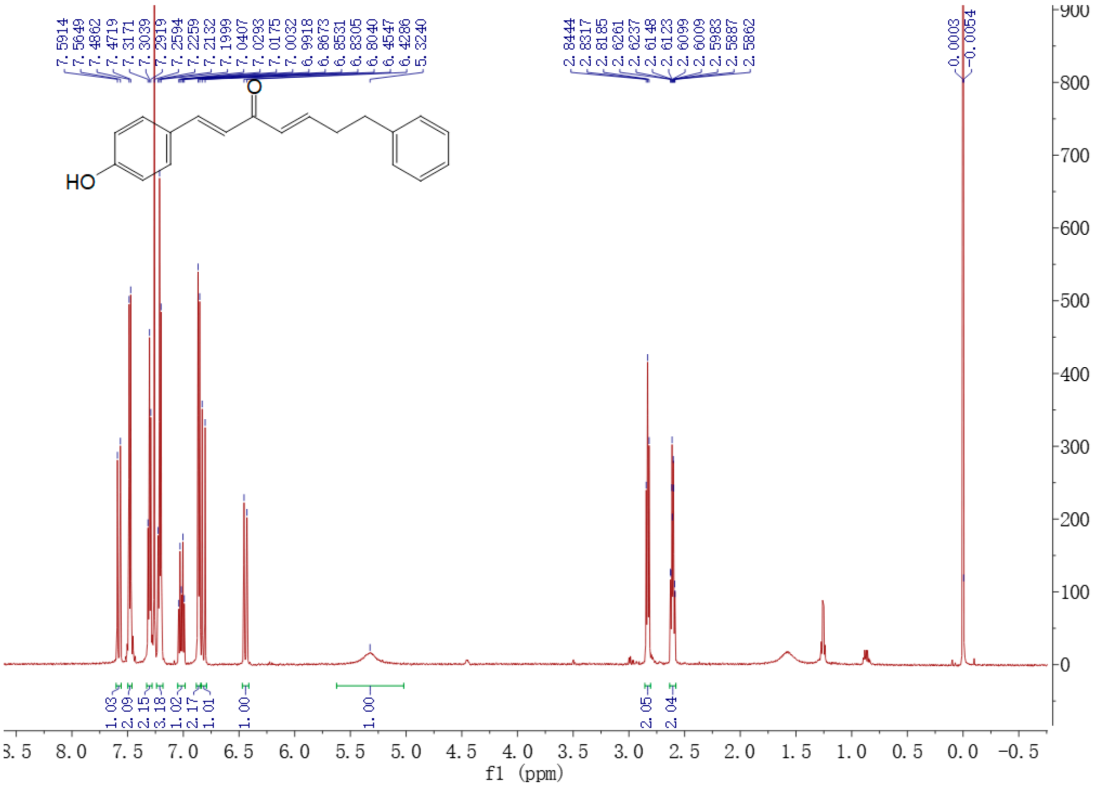

^{1}H NMR spectrum of **CU-7** (600 MHz, $CDCl_3$)

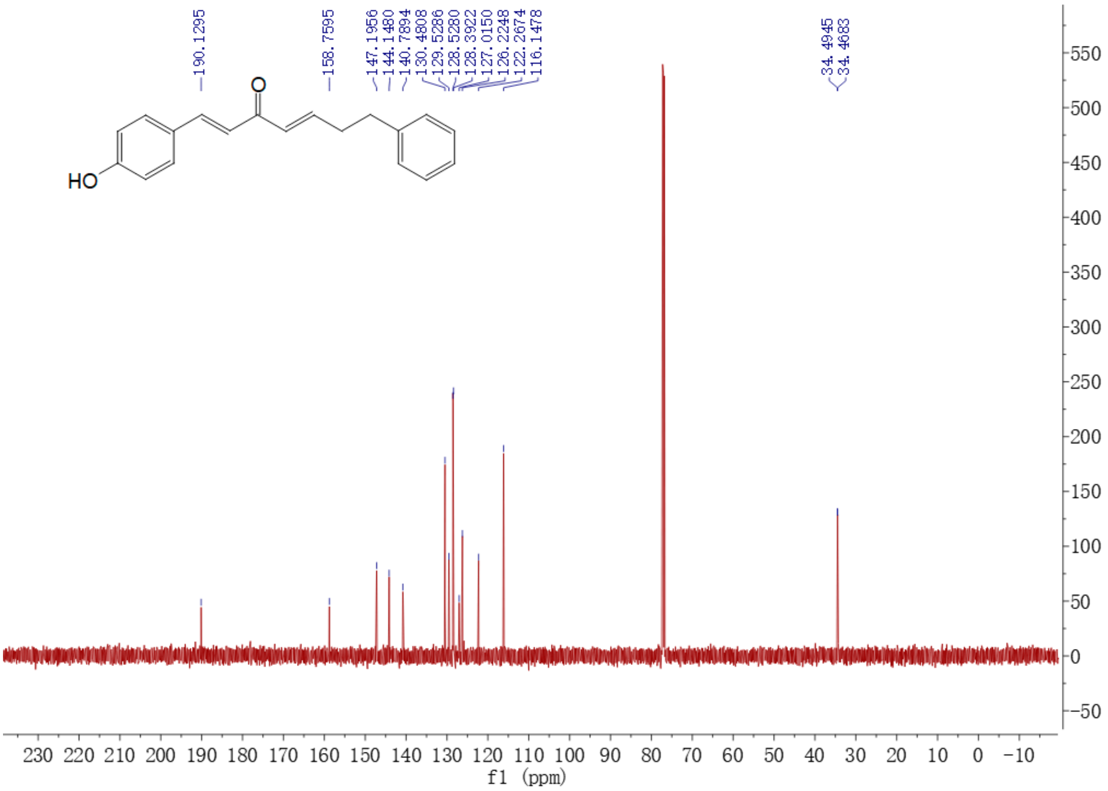

^{13}C NMR spectrum of **CU-7** (151 MHz, $CDCl_3$)

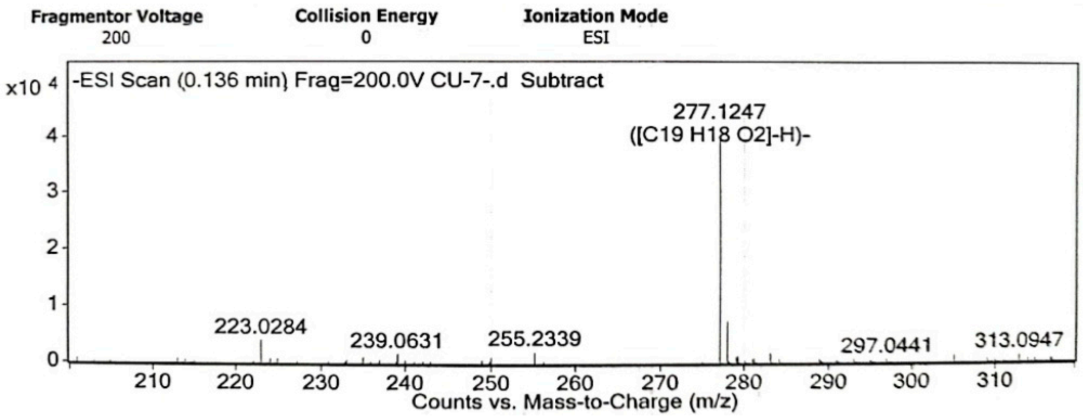

HRMS spectrum of **CU-7**

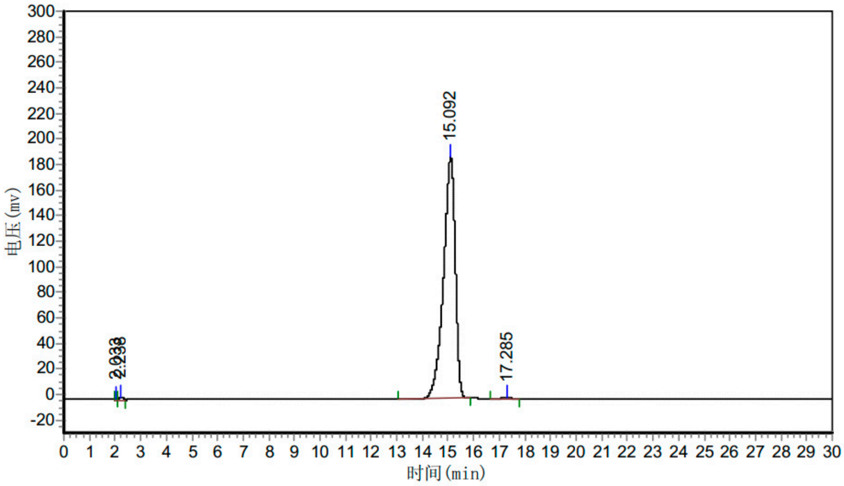

分析结果表

峰号	峰名	保留时间	峰高	峰面积	含量
1		2.033	1032.042	7517.198	0.1233
2		2.238	2266.143	22497.465	0.3690
3		15.092	188682.109	6035332.000	98.9917
4		17.285	1016.723	31461.752	0.5160
总计			192997.017	6096808.415	100.0000

The HPLC spectrum of **CU-7**

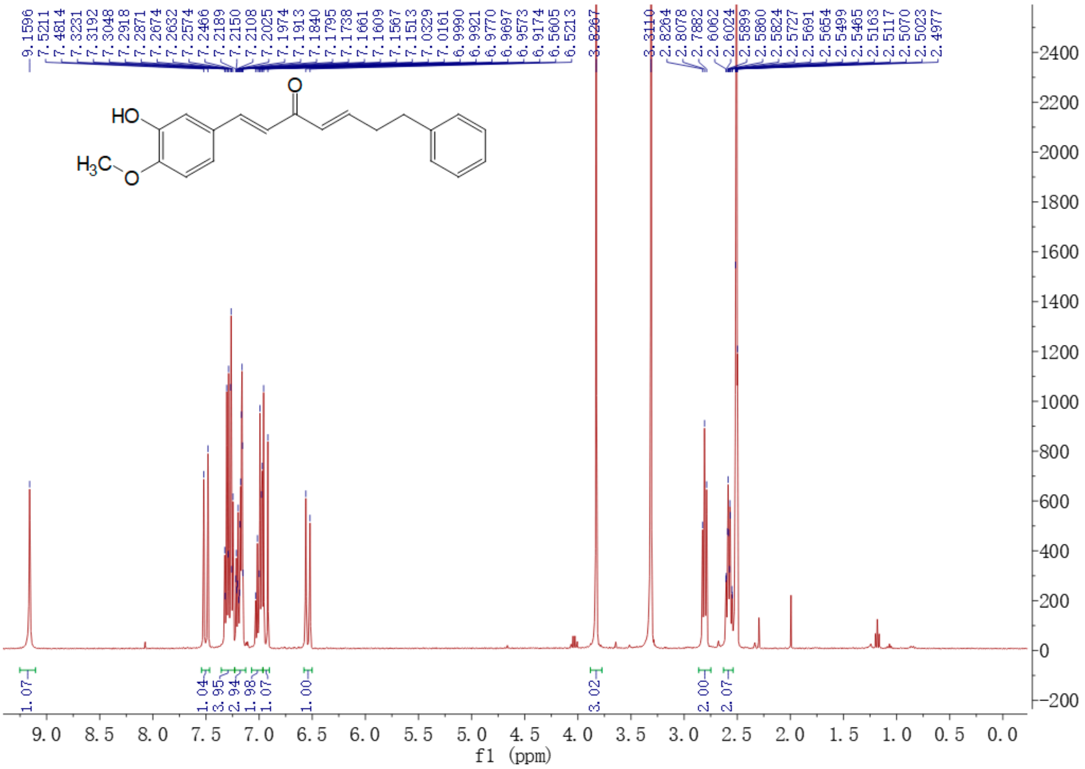

^{1}H NMR spectrum of **CU-8** (600 MHz, DMSO-d_6)

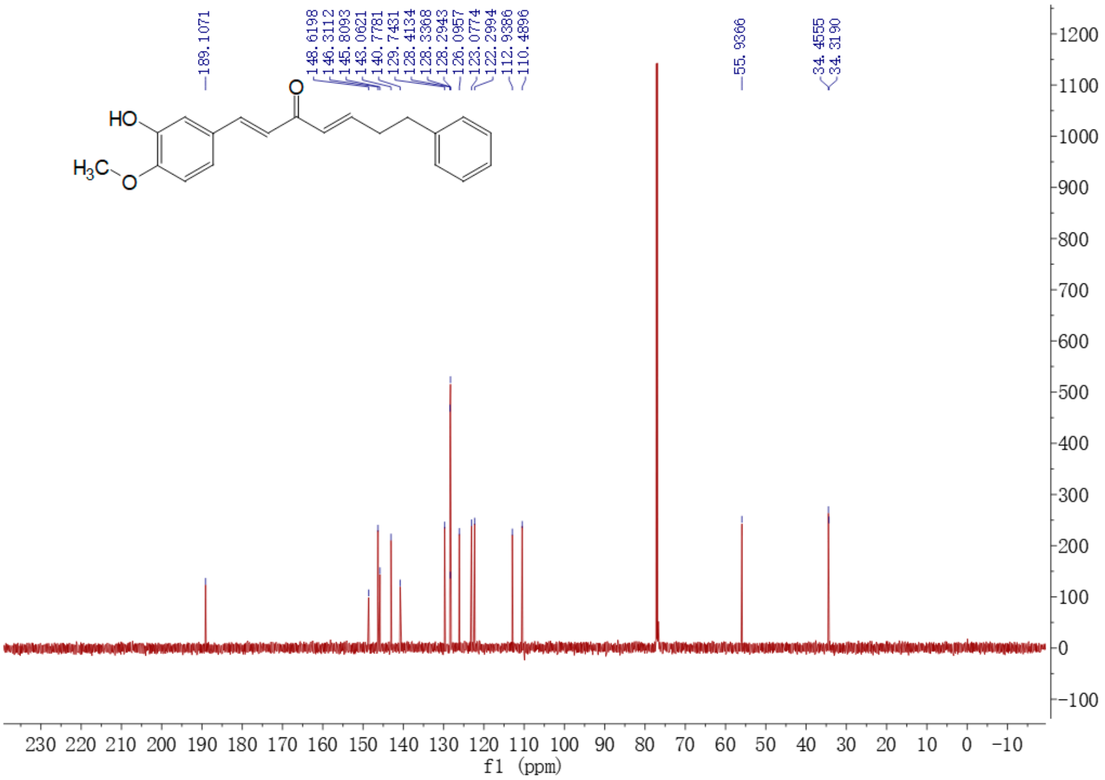

^{13}C NMR spectrum of **CU-8** (151 MHz, $CDCl_3$)

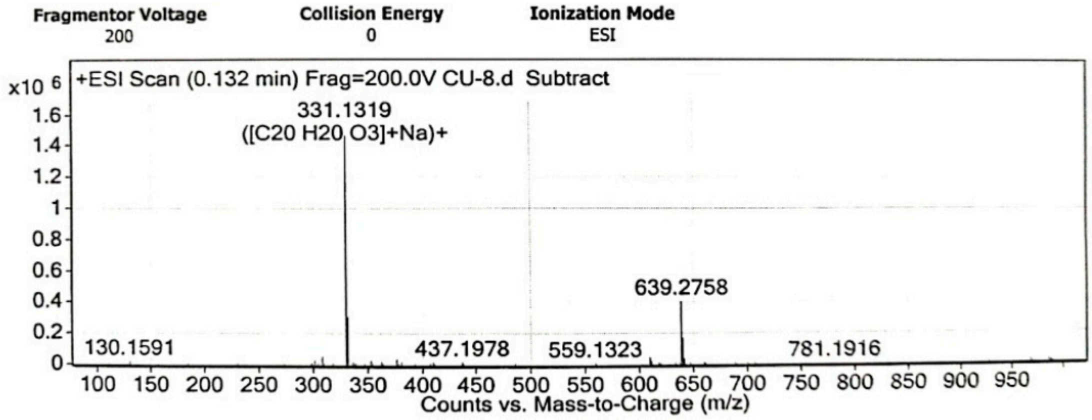

HRMS spectrum of **CU-8**

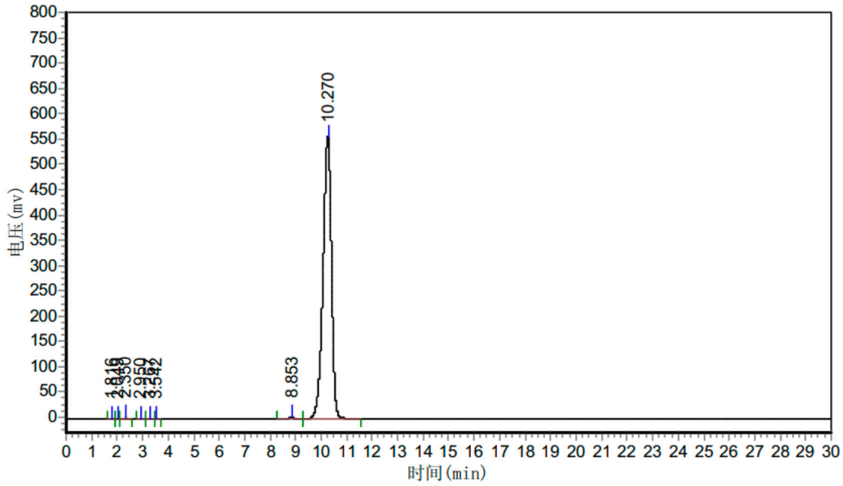

分析结果表

峰号	峰名	保留时间	峰高	峰面积	含量
1		1.816	339.918	3014.010	0.0230
2		2.049	764.809	7876.200	0.0601
3		2.350	2492.955	37321.590	0.2847
4		2.950	1461.650	17055.998	0.1301
5		3.267	1782.750	19170.498	0.1462
6		3.542	468.100	4163.962	0.0318
7		8.853	2412.613	51192.156	0.3905
8		10.270	558719.438	12968676.000	98.9336
总计			568442.233	13108470.414	100.0000

The HPLC spectrum of **CU-8**

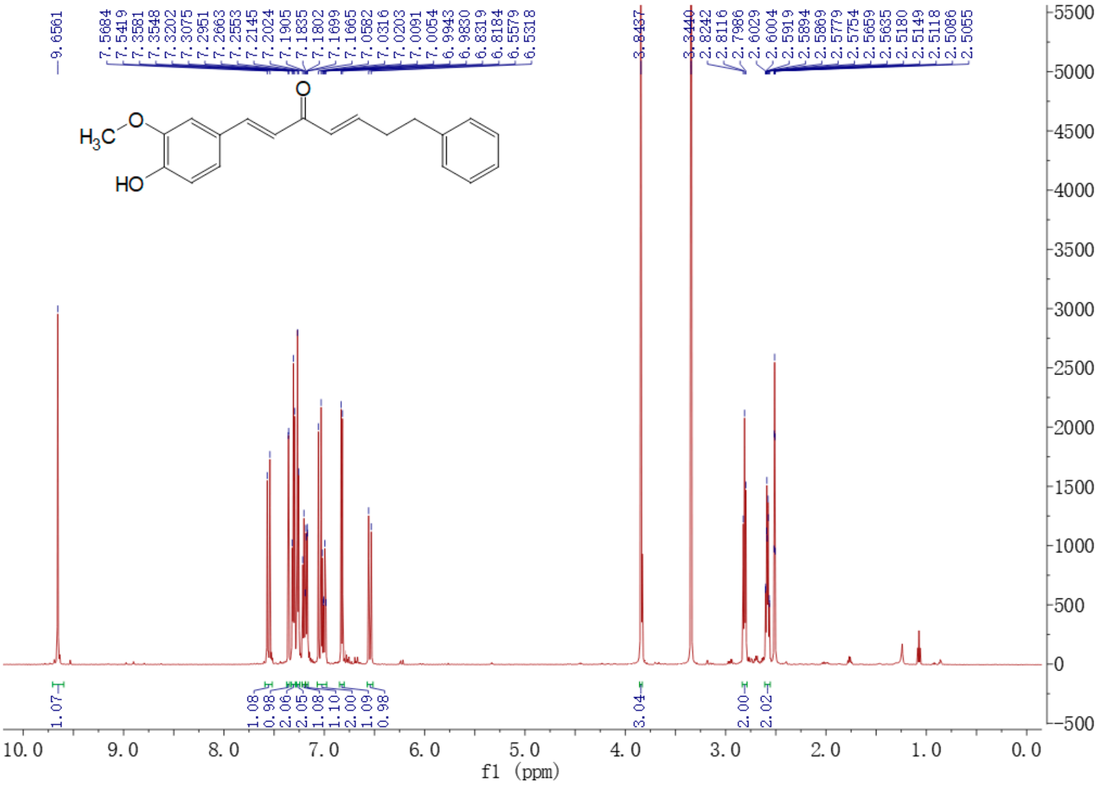

^{1}H NMR spectrum of **CU-9** (600 MHz, DMSO-d_6)

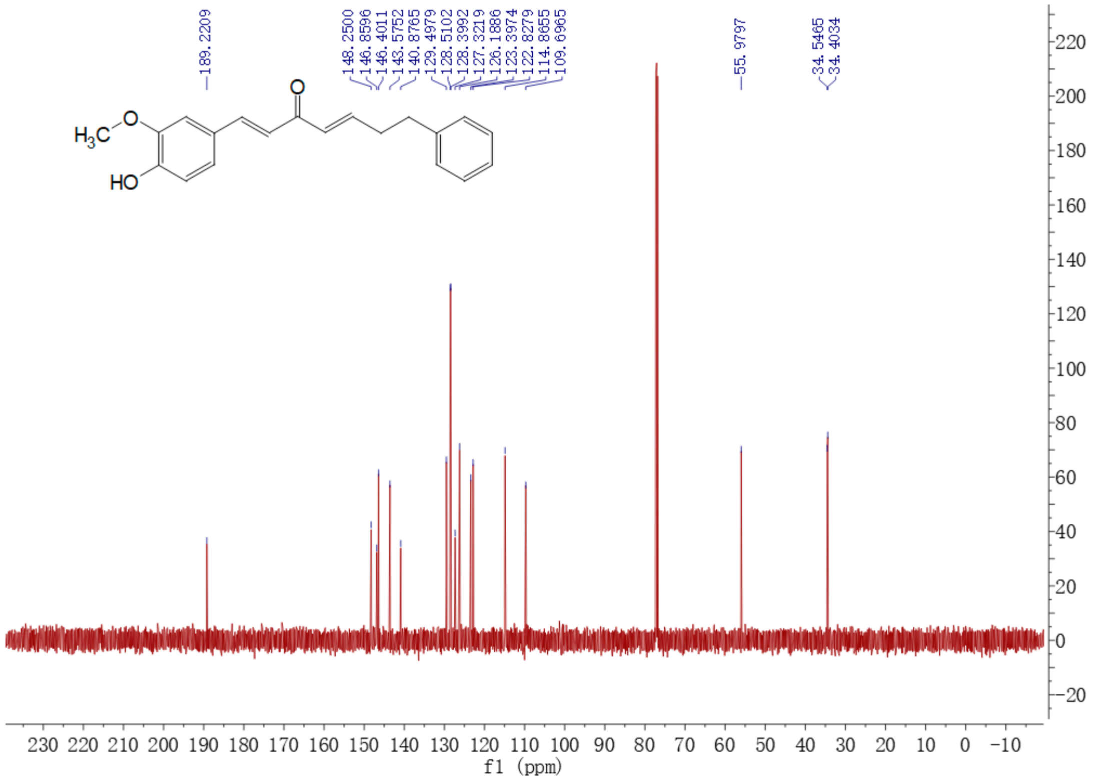

^{13}C NMR spectrum of **CU-9** (151 MHz, $CDCl_3$)

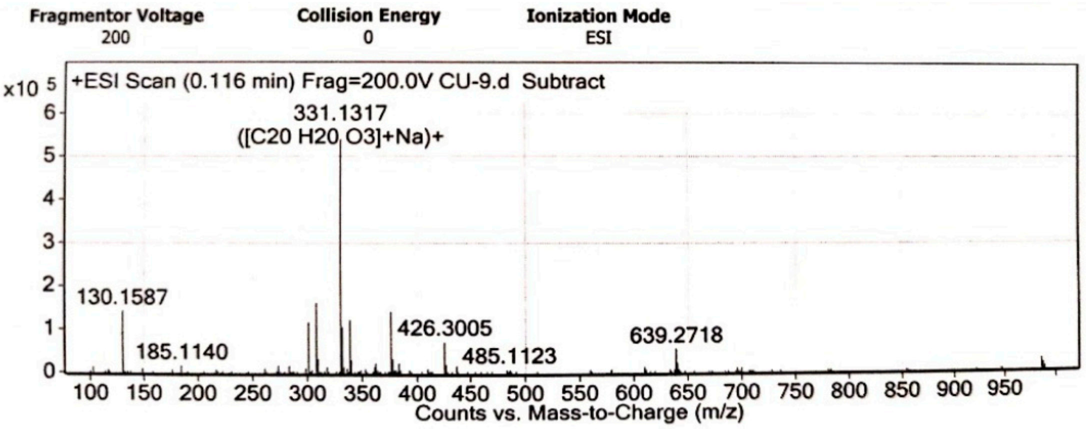

HRMS spectrum of **CU-9**

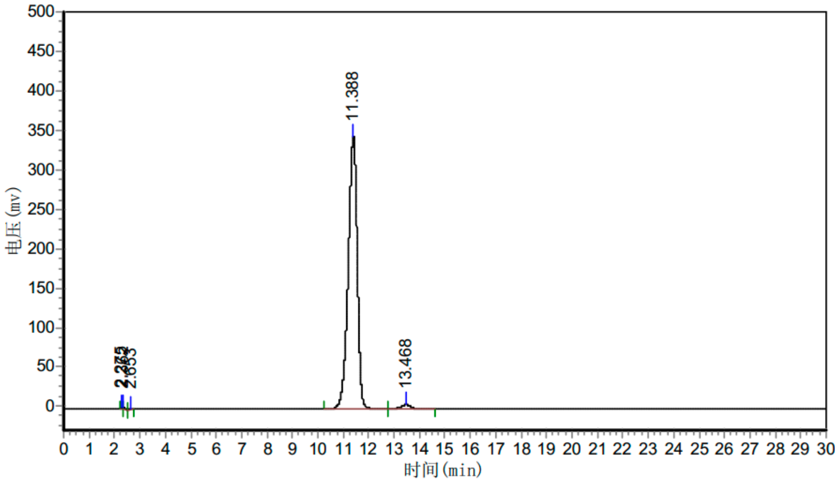

分析结果表

峰号	峰名	保留时间	峰高	峰面积	含量
1		2.275	2056.899	11685.270	0.1324
2		2.362	2077.719	13154.101	0.1491
3		2.653	275.952	3085.696	0.0350
4		11.388	346223.375	8529178.541	96.6559
5		13.468	5794.259	267172.453	3.0277
总计			356428.204	8824270.988	100.0000

The HPLC spectrum of **CU-9**

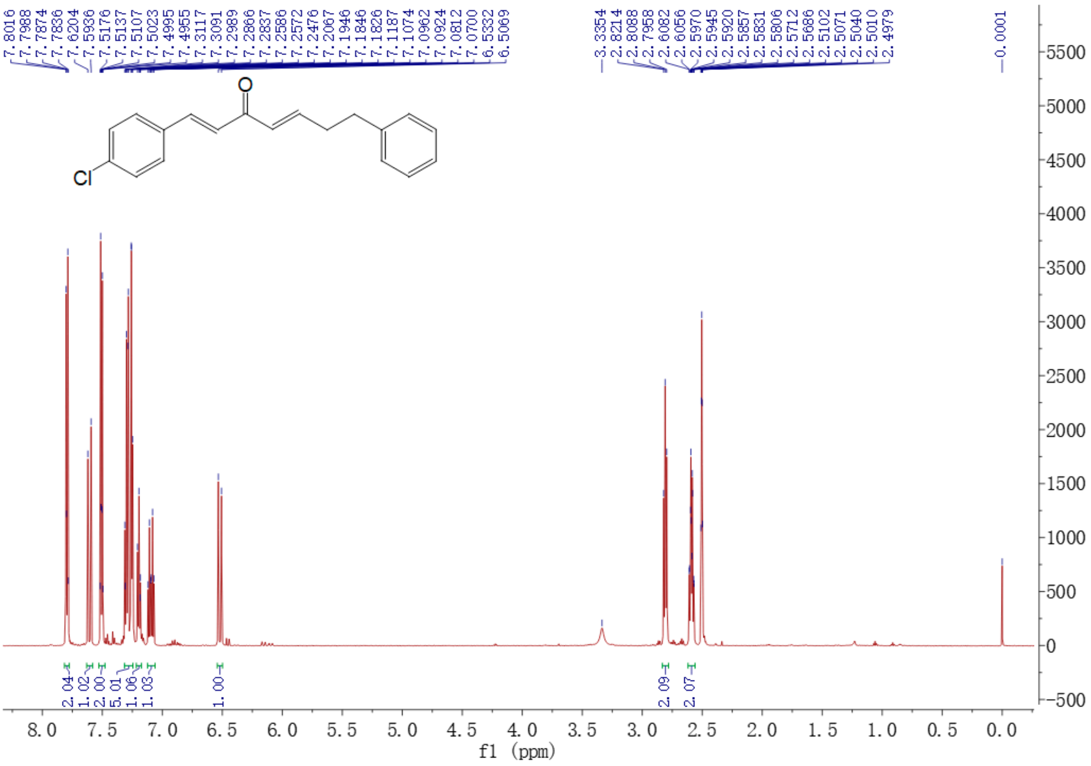

^{1}H NMR spectrum of **CU-10** (600 MHz, $CDCl_3$)

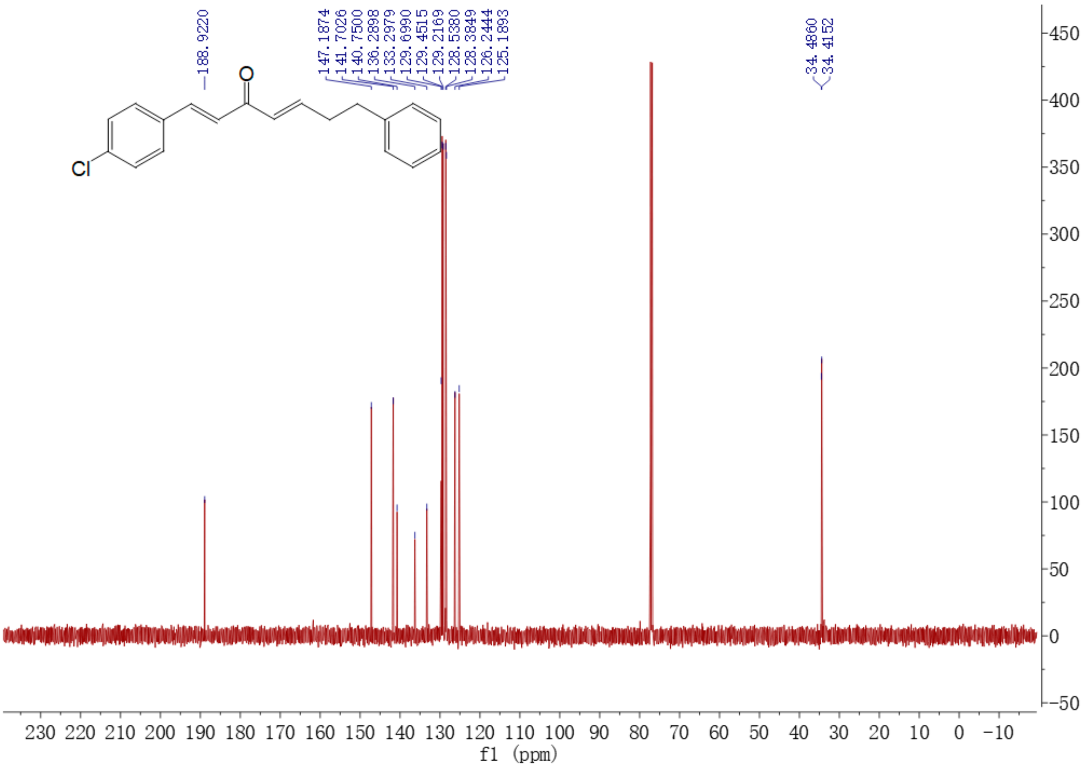

^{13}C NMR spectrum of **CU-10** (151 MHz, $CDCl_3$)

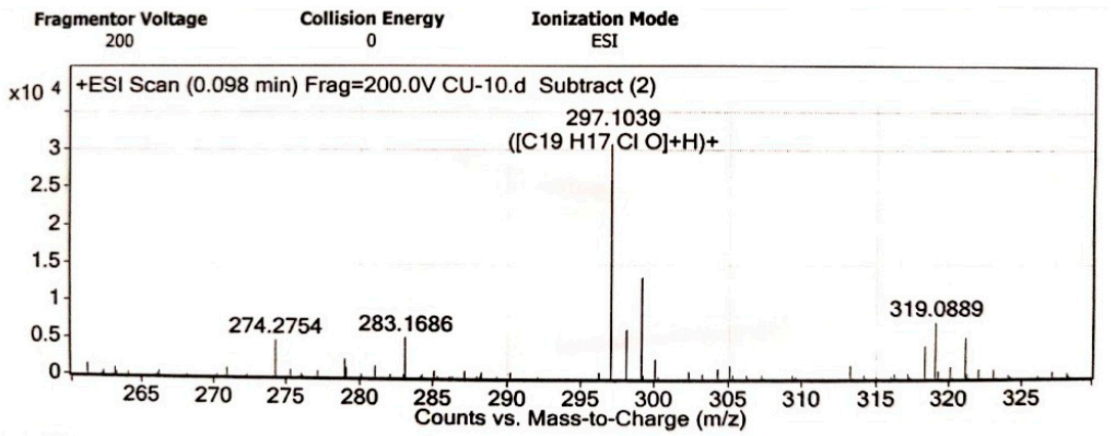

HRMS spectrum of **CU-10**

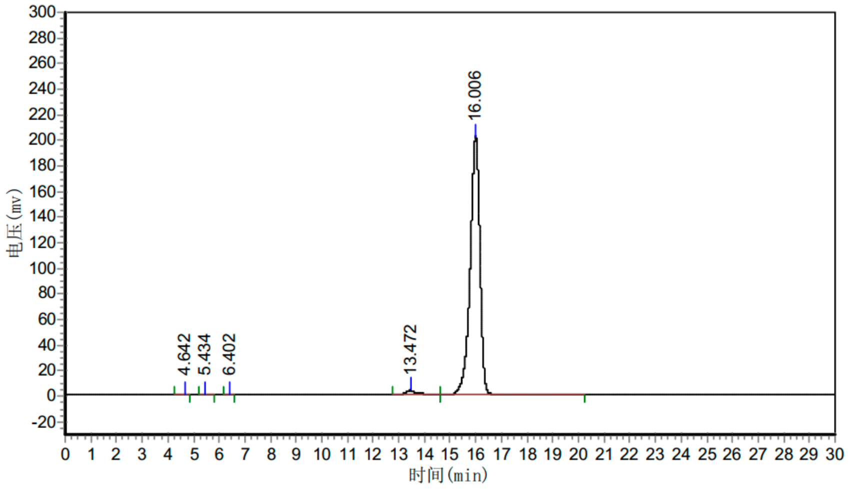

分析结果表

峰号	峰名	保留时间	峰高	峰面积	含量
1		4.642	516.206	4868.100	0.0892
2		5.434	180.751	3799.228	0.0696
3		6.402	183.043	2623.213	0.0481
4		13.472	3398.400	209226.116	3.8332
5		16.006	202235.281	5237748.636	95.9600
总计			206513.681	5458262.439	100.0000

The HPLC spectrum of **CU-10**

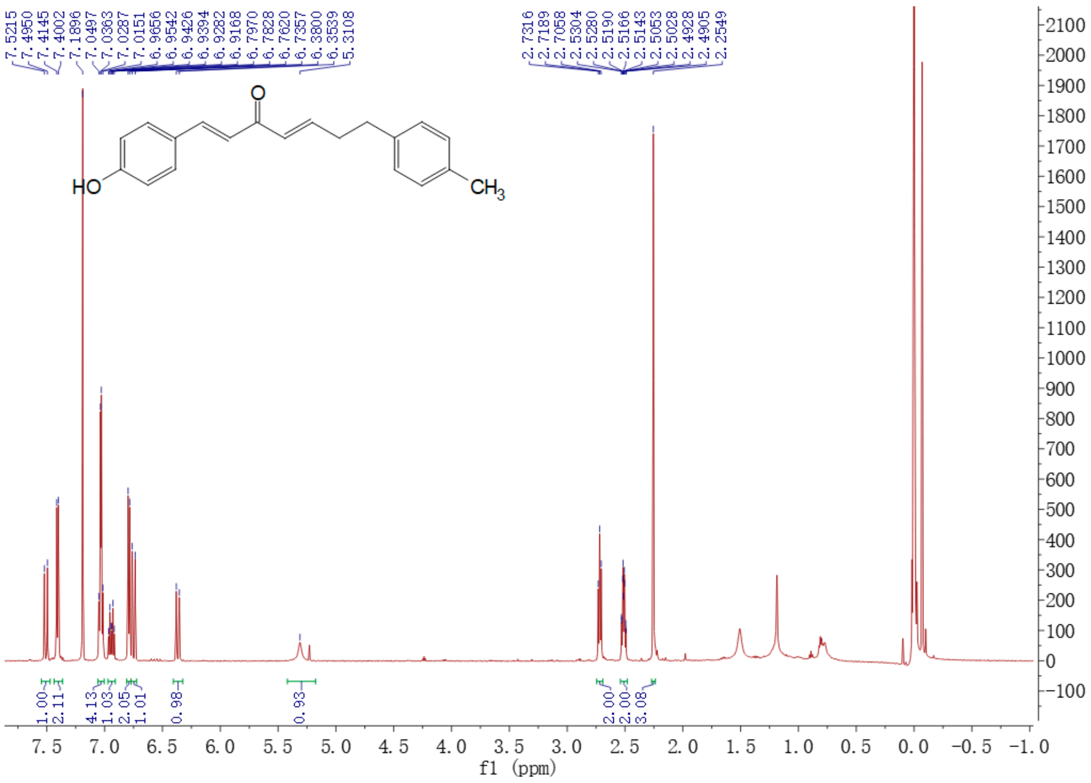

^{1}H NMR spectrum of **CU-11** (600 MHz, $CDCl_3$)

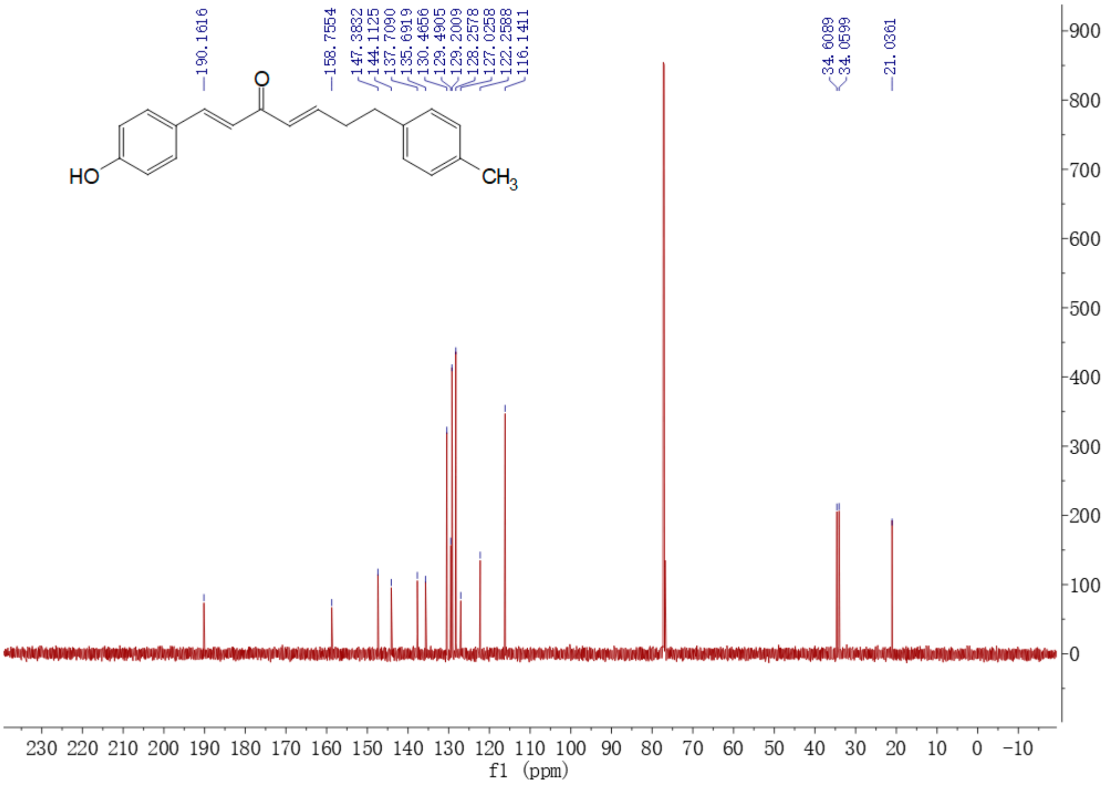

^{13}C NMR spectrum of **CU-11** (151 MHz, $CDCl_3$)

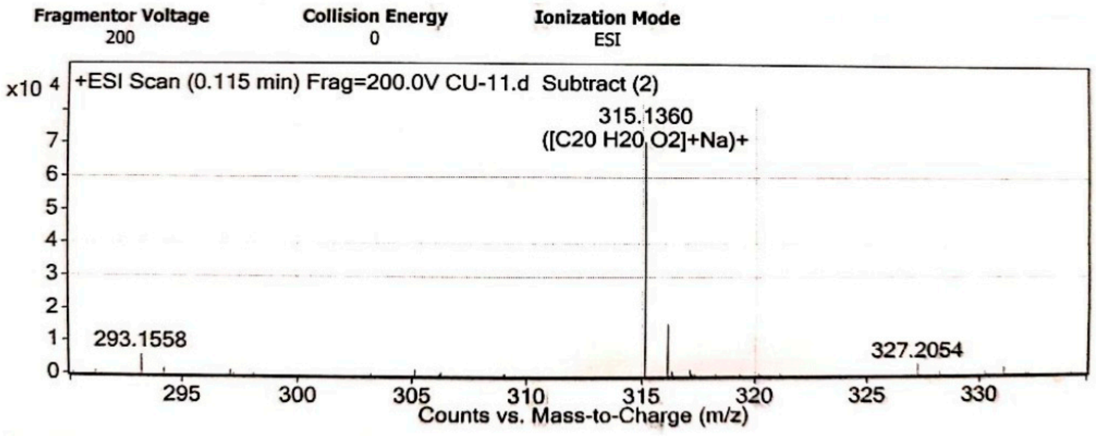

HRMS spectrum of **CU-11**

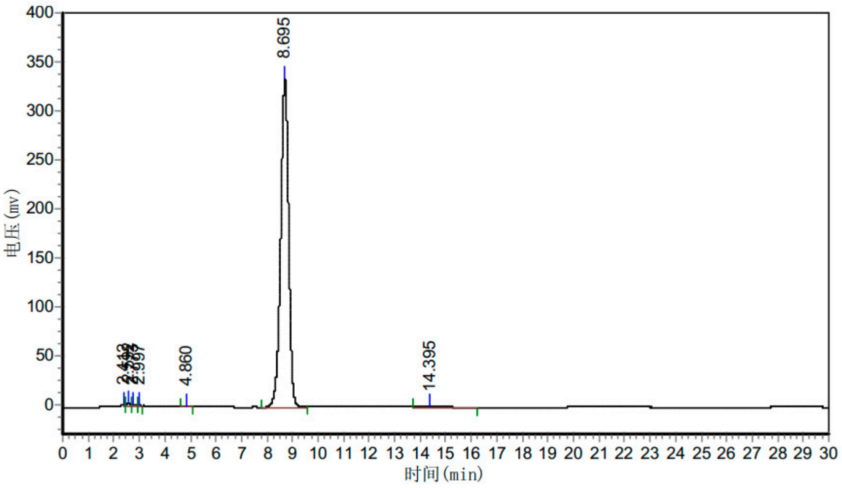

分析结果表

峰号	峰名	保留时间	峰高	峰面积	含量
1		2.412	1550.516	6832.275	0.0918
2		2.592	3946.372	40699.668	0.5470
3		2.773	1628.229	18528.340	0.2490
4		2.997	1707.006	16397.791	0.2204
5		4.860	796.488	8666.198	0.1165
6		8.695	336252.594	7283180.500	97.8804
7		14.395	1547.064	66590.031	0.8949
总计			347428.269	7440894.803	100.0000

The HPLC spectrum of **CU-11**

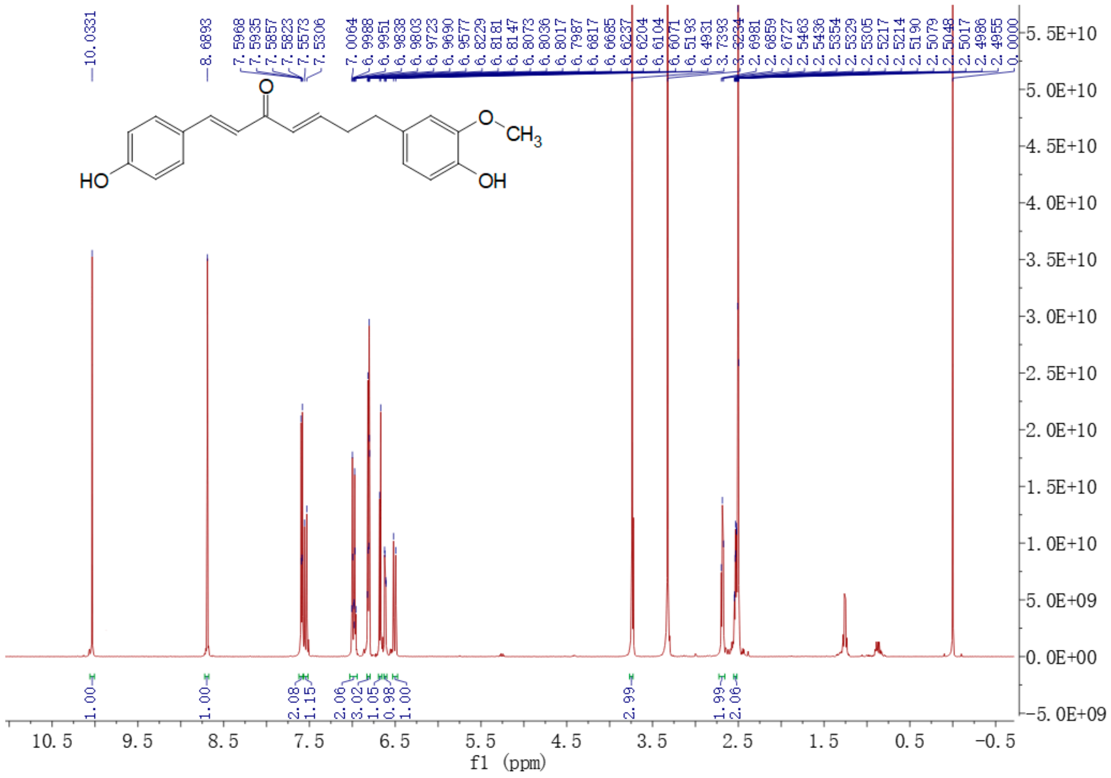

^{1}H NMR spectrum of **CU-12** (600 MHz, DMSO-d_6)

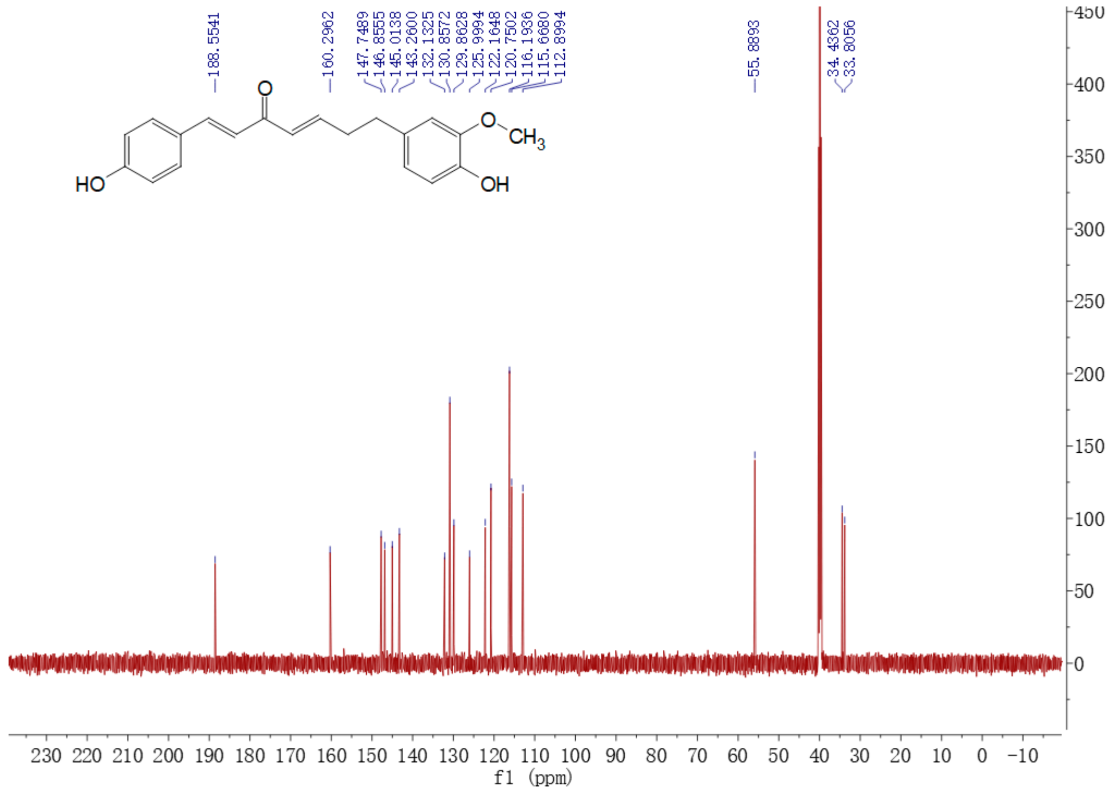

^{13}C NMR spectrum of **CU-12** (151 MHz, DMSO-d_6)

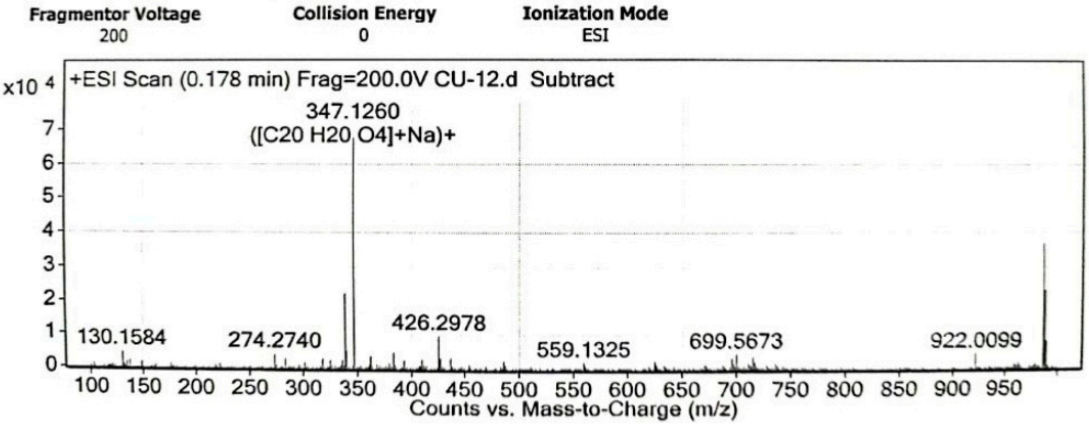

HRMS spectrum of **CU-12**

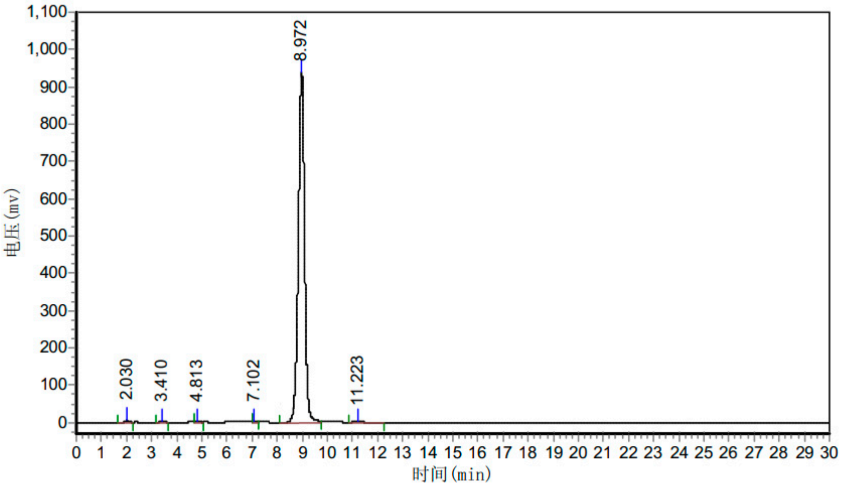

分析结果表

峰号	峰名	保留时间	峰高	峰面积	含量
1		2. 030	5665. 422	64132. 012	0. 3910
2		3. 410	3126. 135	39040. 695	0. 2380
3		4. 813	1458. 074	18318. 861	0. 1117
4		7. 102	2830. 337	41172. 398	0. 2510
5		8. 972	939980. 563	16199390. 000	98. 7727
6		11. 223	1396. 281	38629. 465	0. 2355
总计			954456. 811	16400683. 432	100. 0000

The HPLC spectrum of **CU-1**2

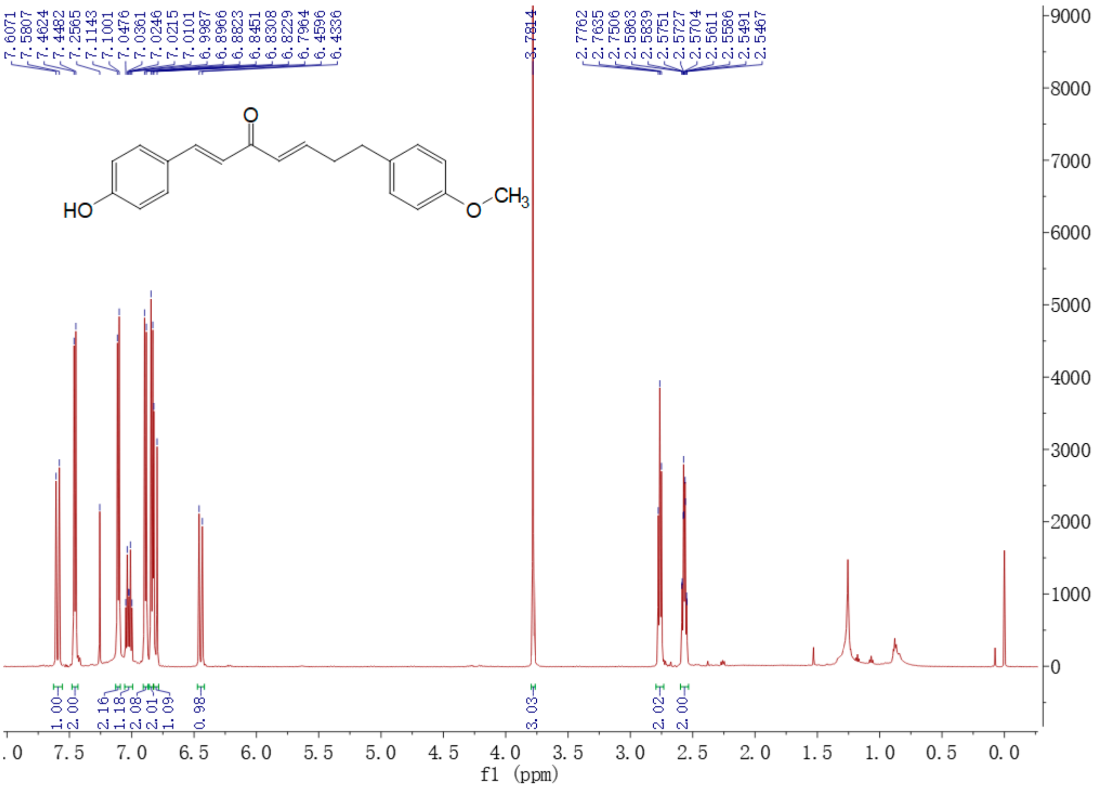

^{1}H NMR spectrum of **CU-13** (600 MHz, $CDCl_3$)

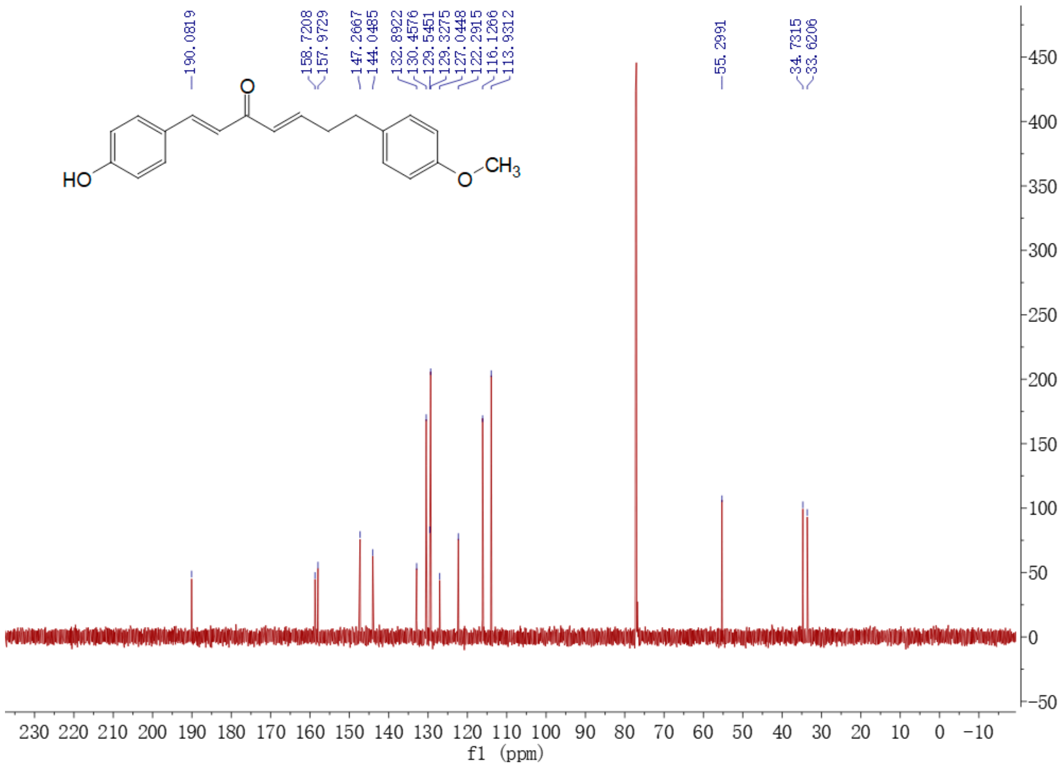

^{13}C NMR spectrum of **CU-13** (151 MHz, $CDCl_3$)

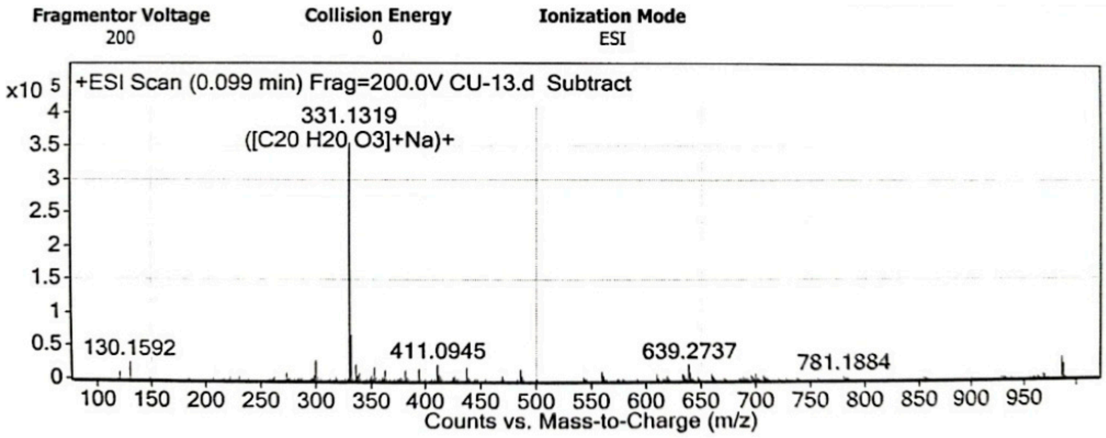

HRMS spectrum of **CU-13**

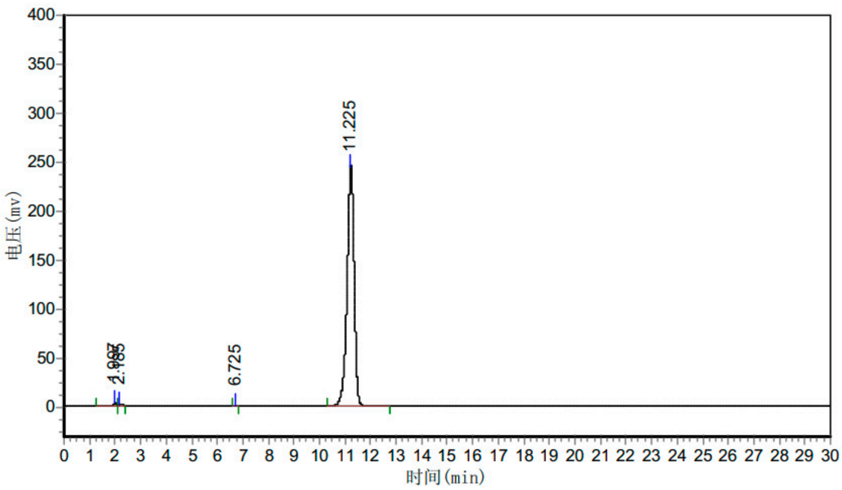

分析结果表

峰号	峰名	保留时间	峰高	峰面积	含量
1		1.997	4029.980	39066.336	0.8170
2		2.185	2475.585	26860.914	0.5618
3		6.725	18.771	163.950	0.0034
4		11.225	245071.781	4715405.000	98.6178
总计			251596.117	4781496.200	100.0000

The HPLC spectrum of **CU-13**

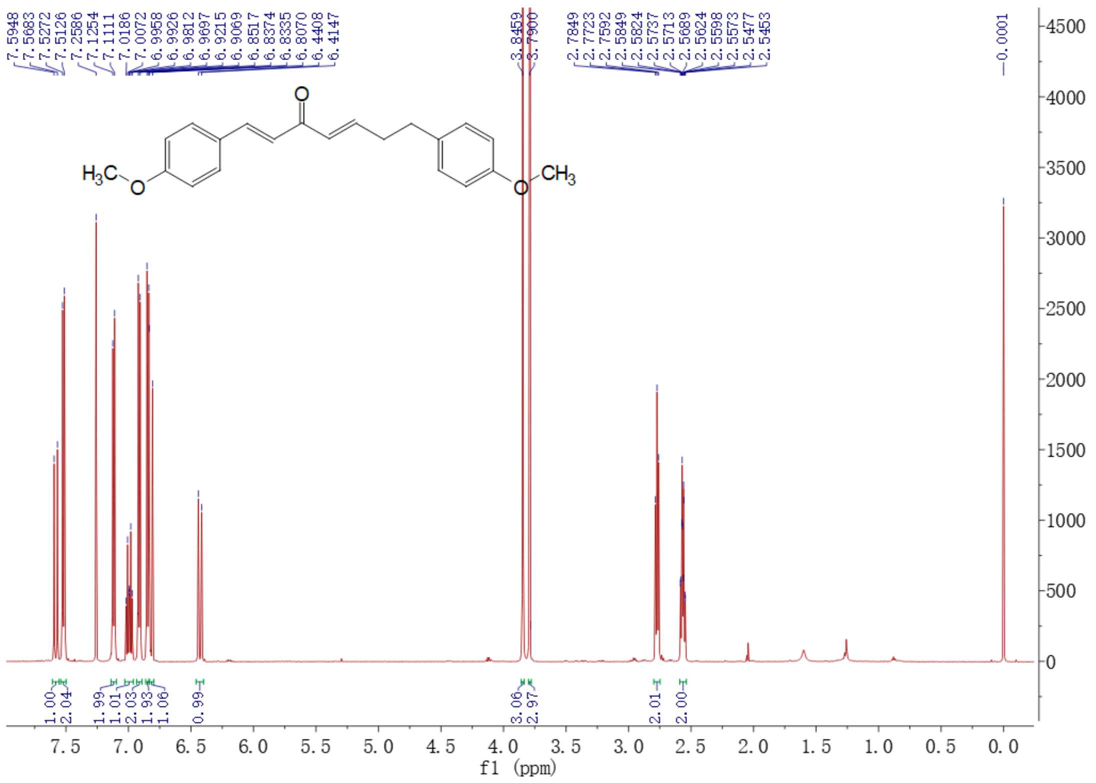

^{1}H NMR spectrum of **CU-14** (600 MHz, $CDCl_3$)

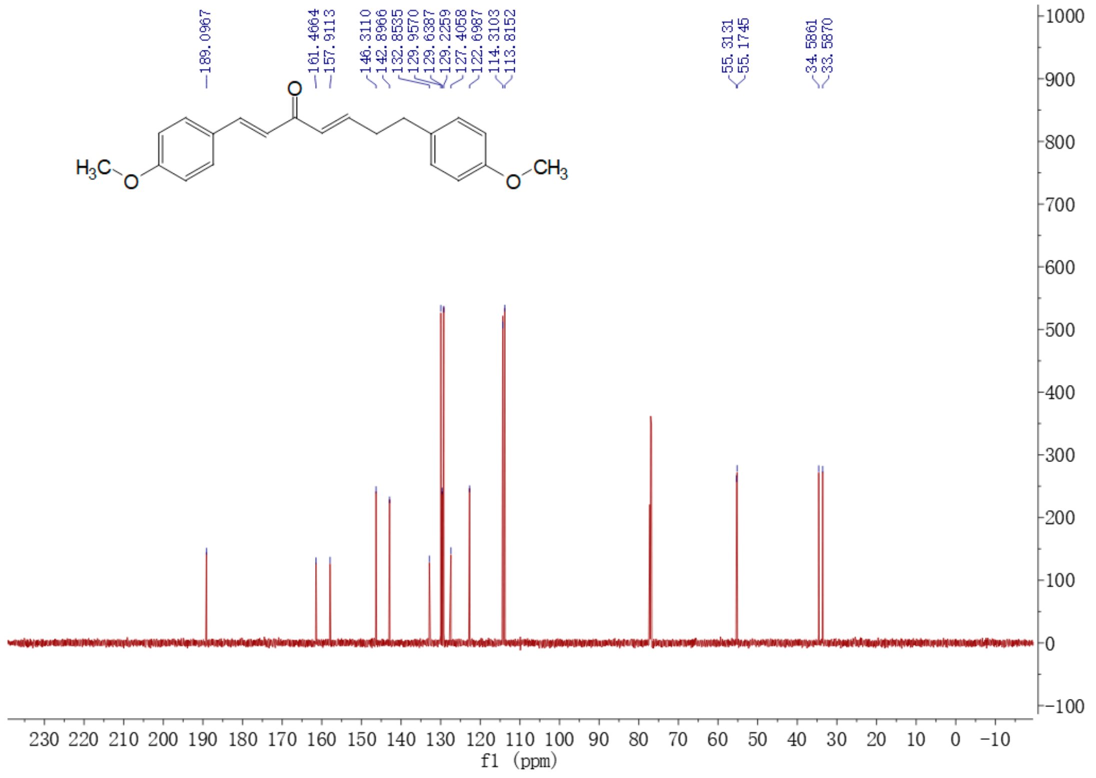

^{13}C NMR spectrum of **CU-14** (151 MHz, $CDCl_3$)

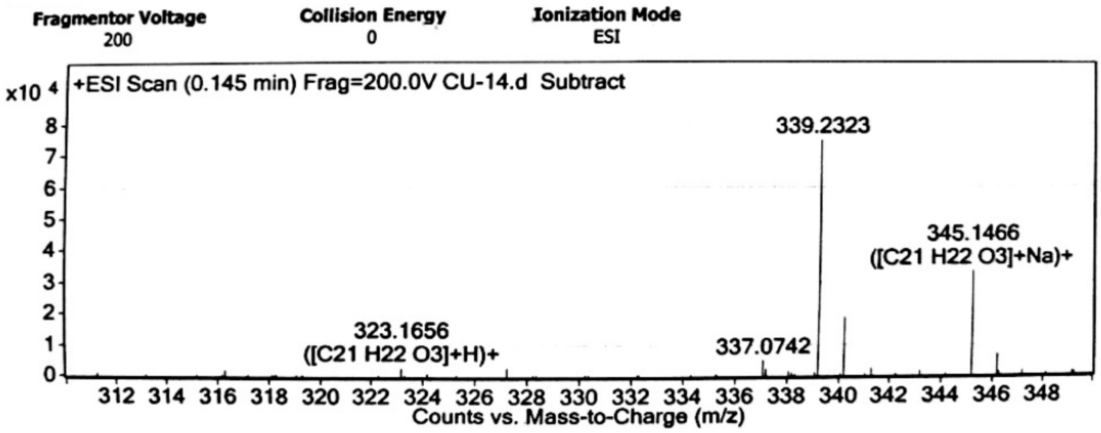

HRMS spectrum of **CU-14**

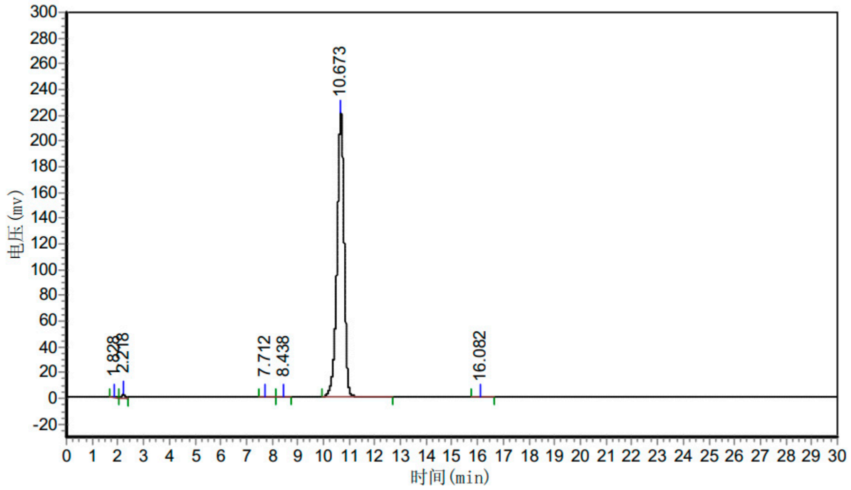

分析结果表

峰号	峰名	保留时间	峰高	峰面积	含量
1		1.828	1090.357	16324.590	0.3779
2		2.218	2791.281	27769.631	0.6428
3		7.712	193.008	4214.891	0.0976
4		8.438	188.052	3209.481	0.0743
5		10.673	221175.578	4267936.500	98.7876
6		16.082	35.673	861.950	0.0200
总计			225473.949	4320317.043	100.0000

The HPLC spectrum of **CU-14**

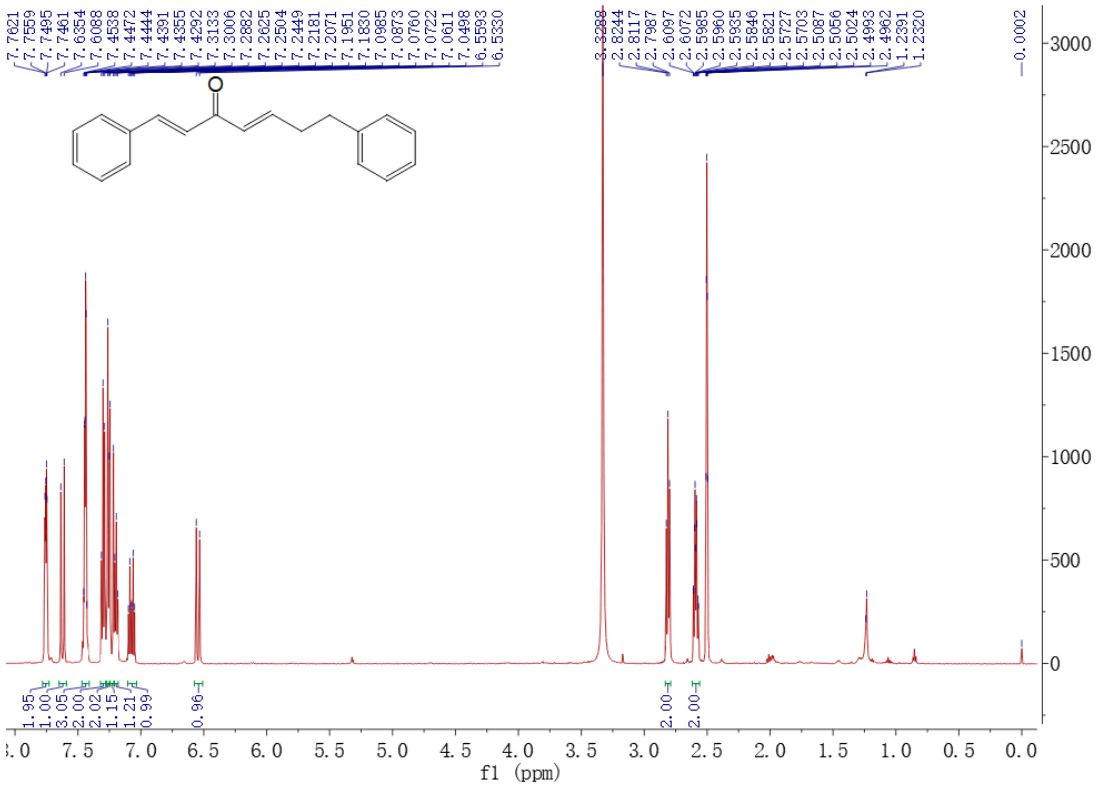

^{1}H NMR spectrum of **CU-15** (600 MHz, DMSO-d_6)

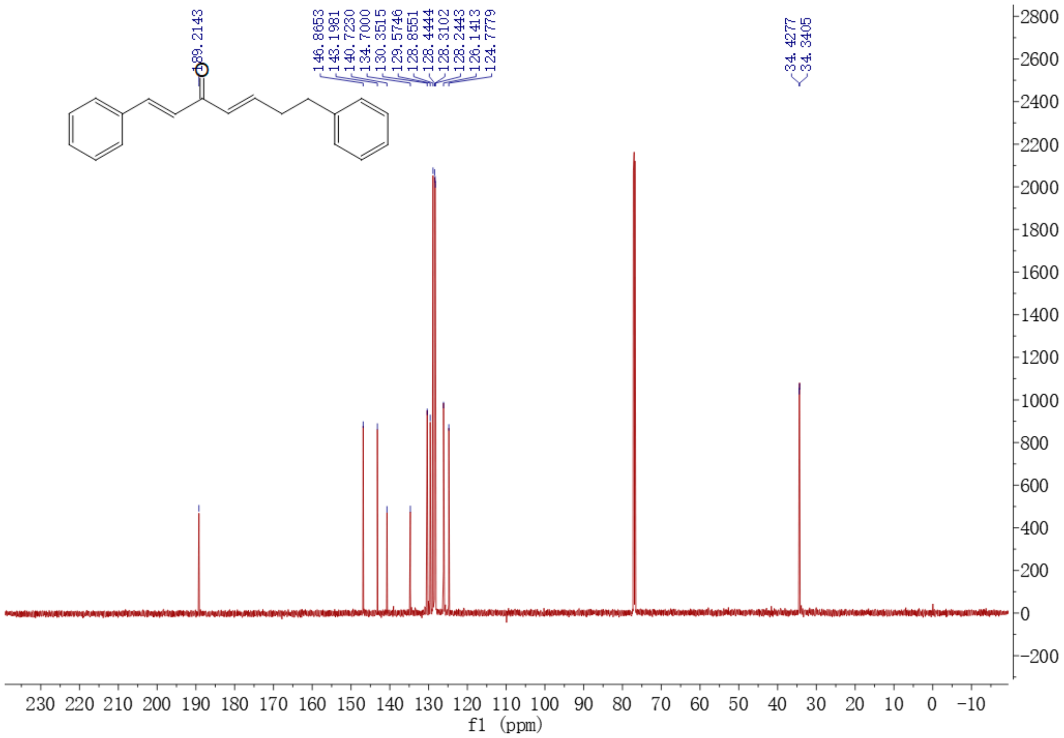

^{13}C NMR spectrum of **CU-15** (151 MHz, $CDCl_3$)

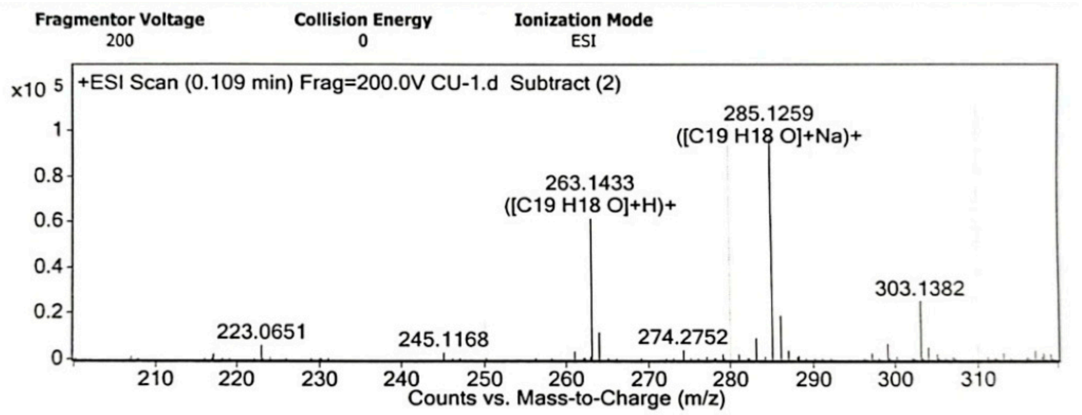

HRMS spectrum of **CU-15**

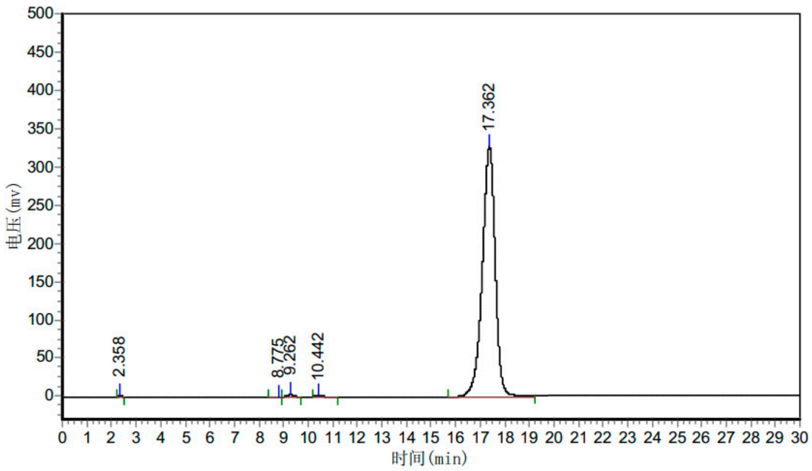

分析结果表

峰号	峰名	保留时间	峰高	峰面积	含量
1		2. 358	2468. 408	23122. 396	0. 1904
2		8. 775	590. 802	9853. 272	0. 0811
3		9. 262	2853. 672	66528. 758	0. 5478
4		10. 442	1599. 628	46737. 375	0. 3849
5		17. 362	328269. 375	11997702. 000	98. 7958
总计			335781. 885	12143943. 802	100. 0000

The HPLC spectrum of **CU-15**

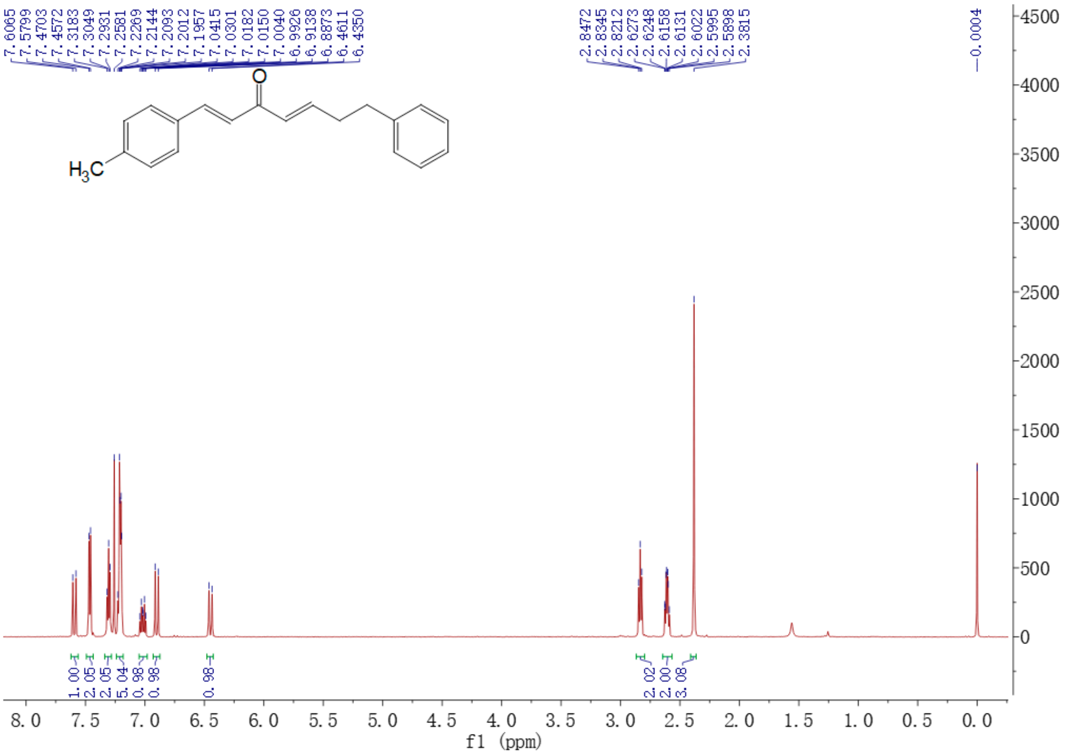

^{1}H NMR spectrum of **CU-16** (600 MHz, $CDCl_3$)

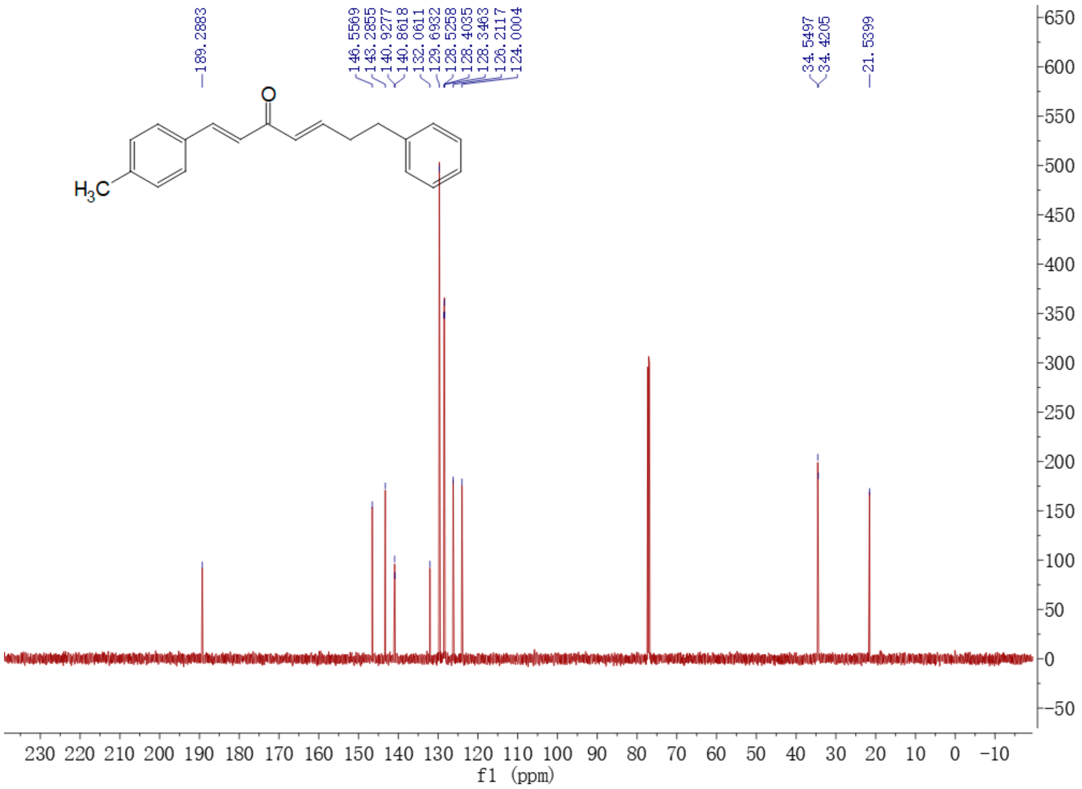

^{13}C NMR spectrum of **CU-16** (151 MHz, $CDCl_3$)

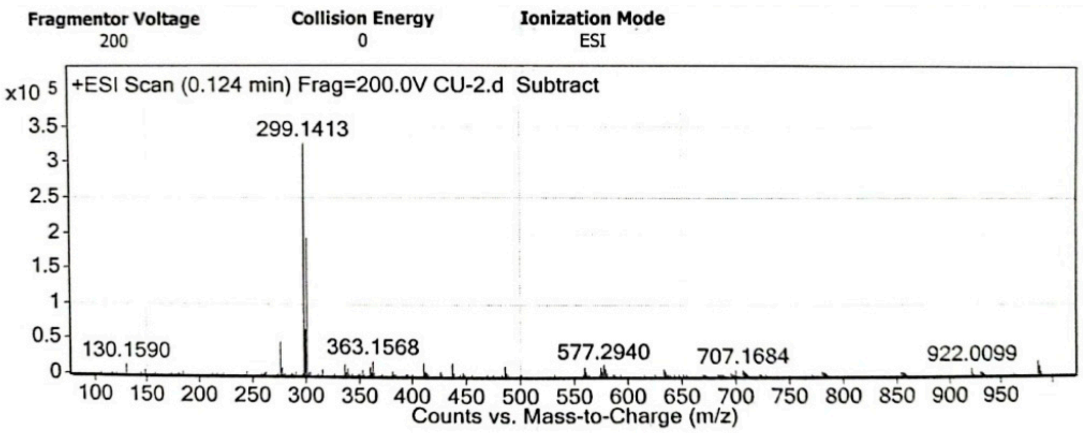

HRMS spectrum of **CU-16**

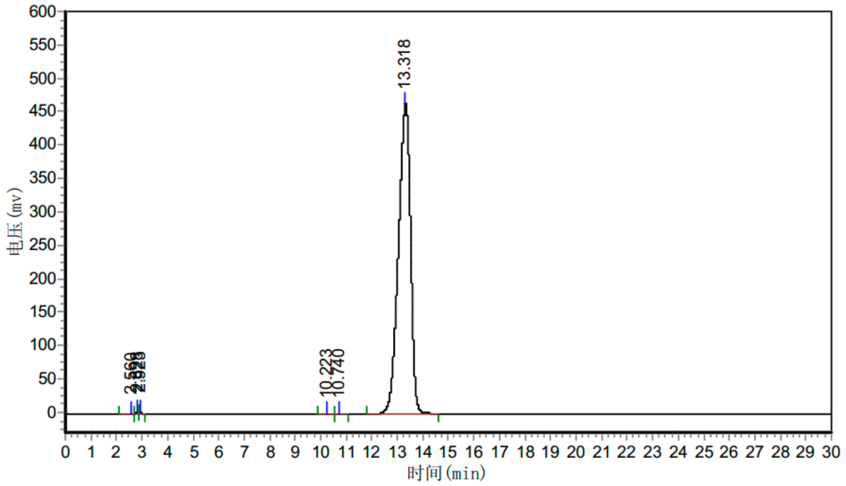

分析结果表

峰号	峰名	保留时间	峰高	峰面积	含量
1		2. 560	1155. 828	15794. 859	0. 1024
2		2. 828	2396. 271	20468. 115	0. 1327
3		2. 923	3438. 740	26358. 938	0. 1709
4		10. 223	76. 064	1574. 164	0. 0102
5		10. 740	66. 494	1430. 696	0. 0093
6		13. 318	464130. 813	15360320. 000	99. 5746
总计			471264. 210	15425946. 772	100. 0000

The HPLC spectrum of **CU-16**

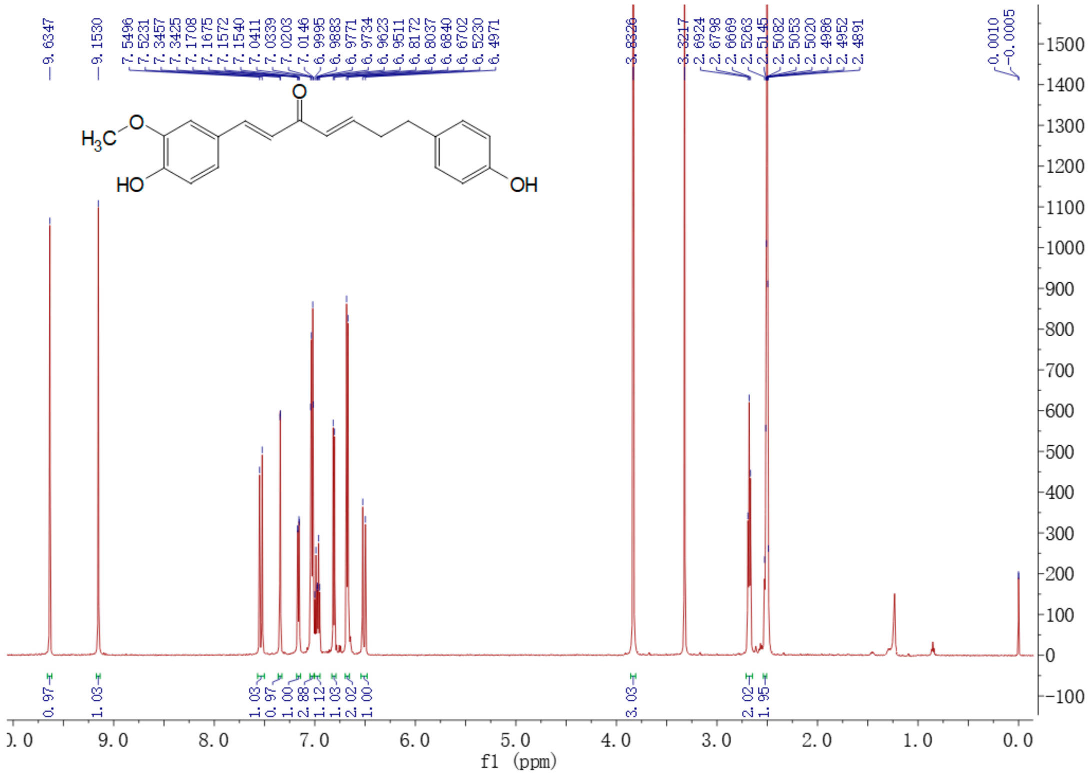

^{1}H NMR spectrum of **CU-17** (600 MHz, DMSO-d_6)

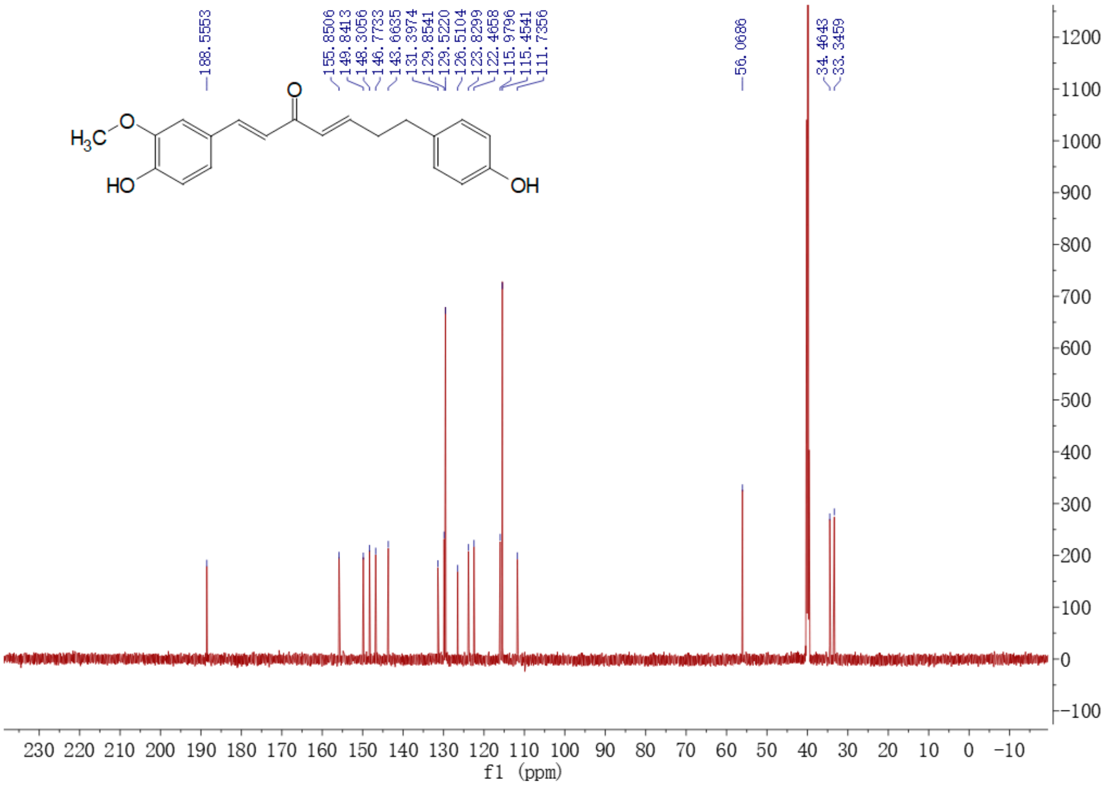

^{13}C NMR spectrum of **CU-17** (151 MHz, DMSO-d_6)

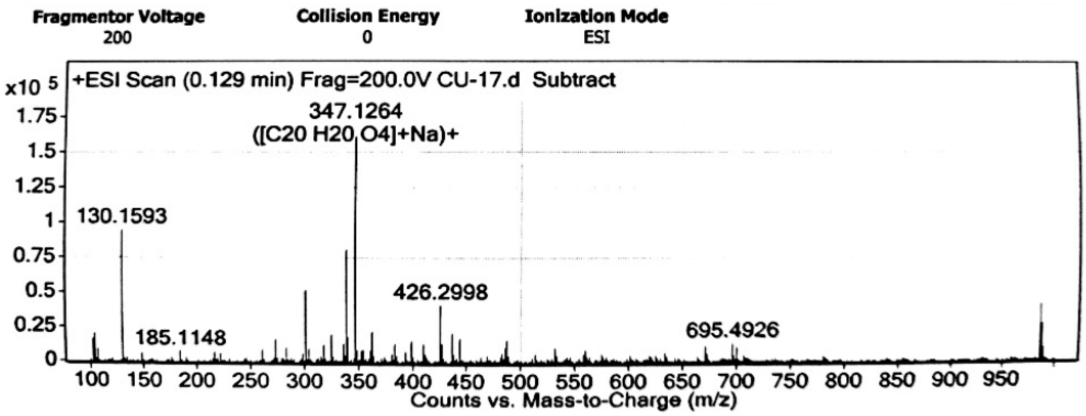

HRMS spectrum of **CU-17**

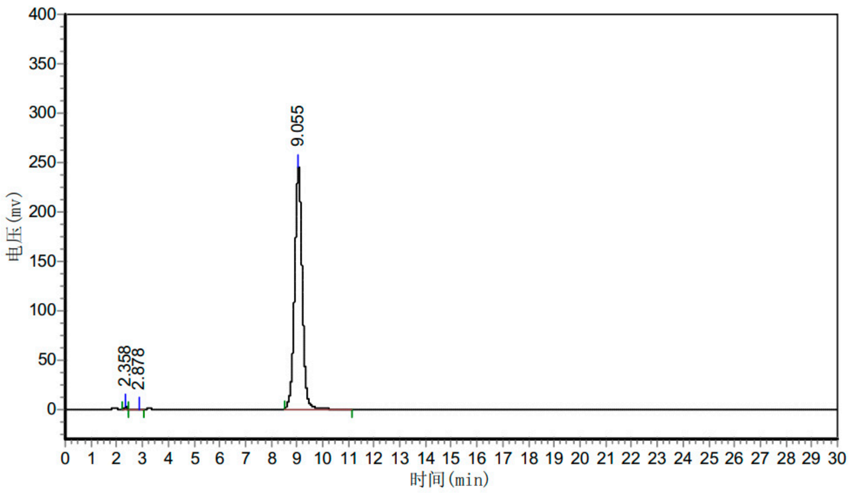

分析结果表

峰号	峰名	保留时间	峰高	峰面积	含量
1		2.358	3597.047	32145.783	0.6201
2		2.878	696.289	24007.377	0.4631
3		9.055	245961.563	5127918.000	98.9168
总计			250254.899	5184071.160	100.0000

The HPLC spectrum of **CU-17**

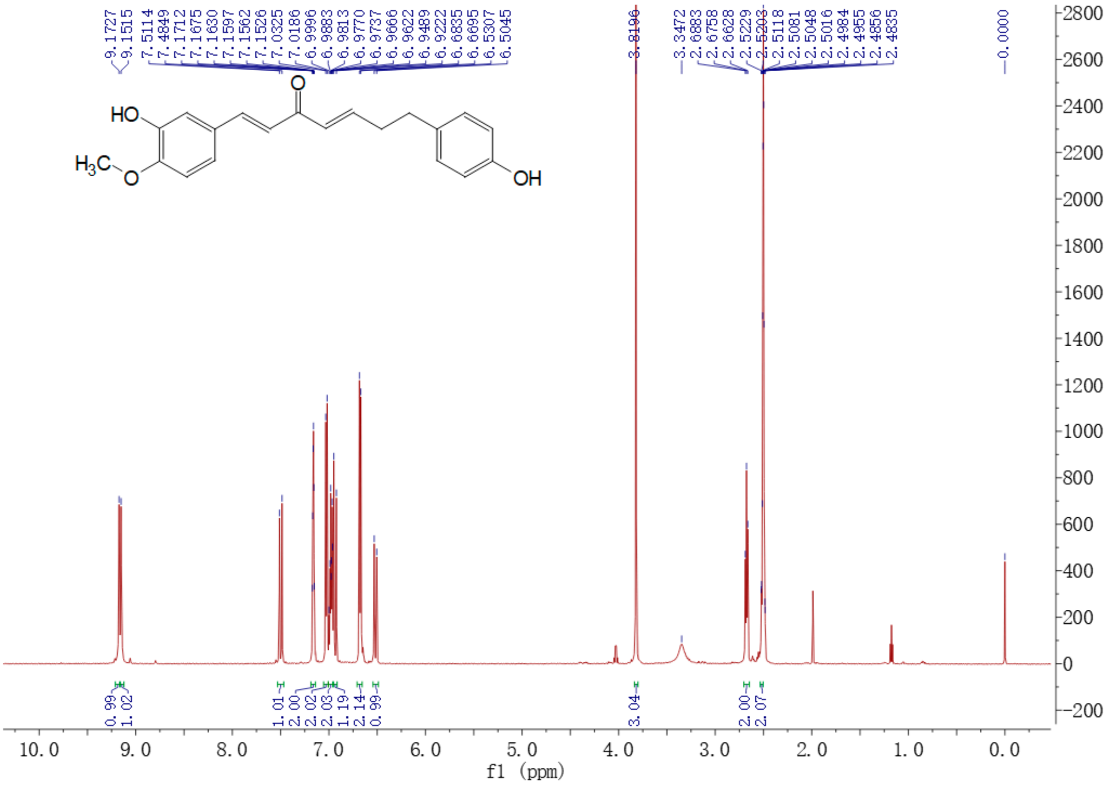

^{1}H NMR spectrum of **CU-18** (600 MHz, DMSO-d_6)

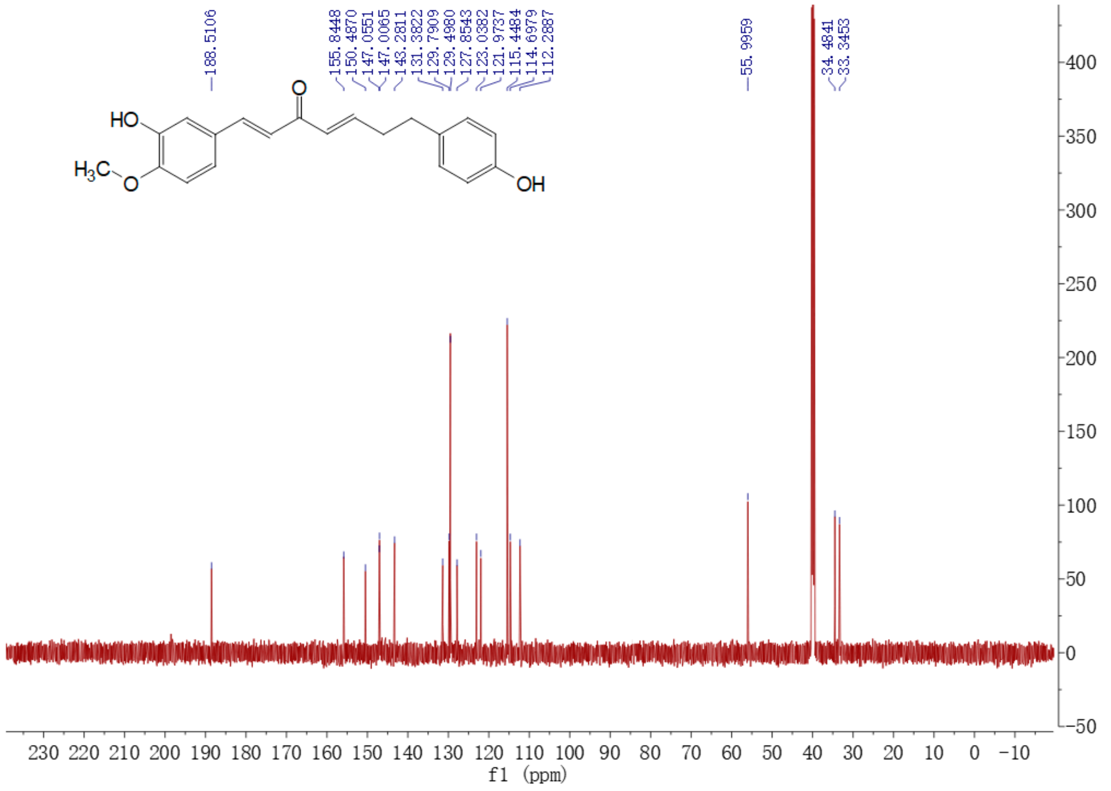

^{13}C NMR spectrum of **CU-18** (151 MHz, DMSO-d_6)

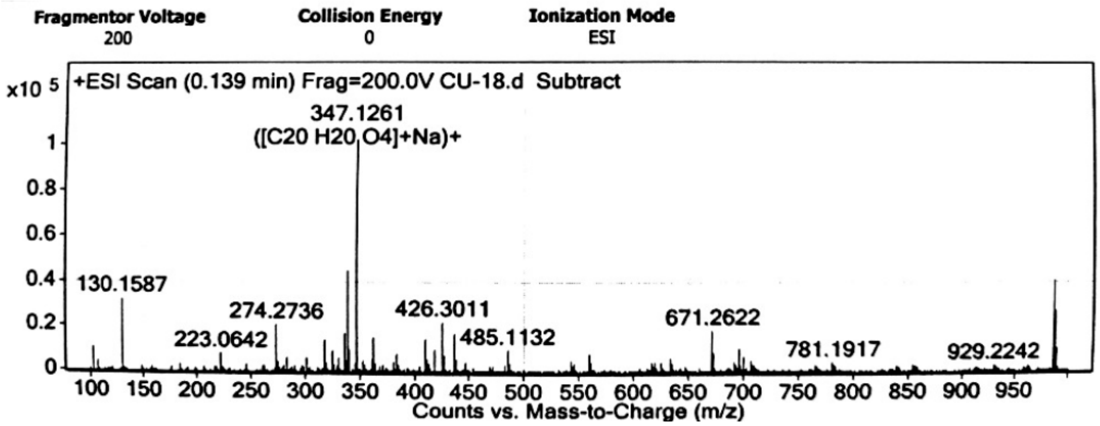

HRMS spectrum of **CU-18**

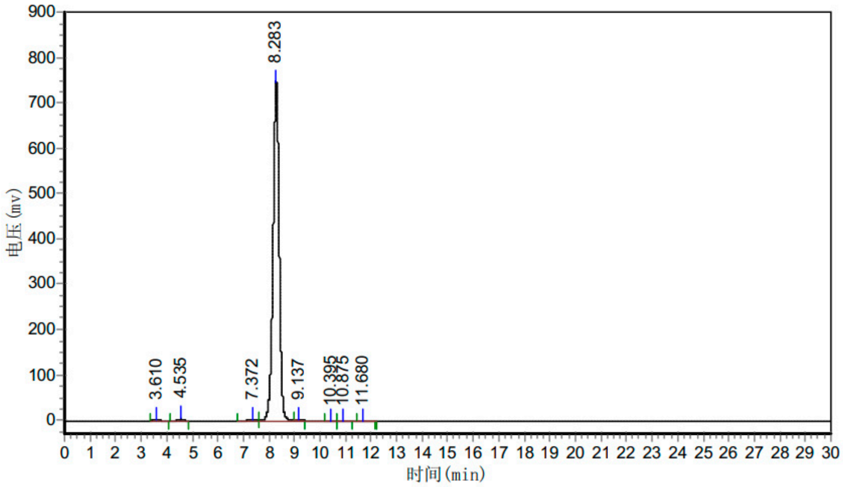

分析结果表

峰号	峰名	保留时间	峰高	峰面积	含量
1		3.610	1992.307	35446.059	0.2789
2		4.535	3685.352	47547.102	0.3742
3		7.372	1138.950	29954.449	0.2357
4		8.283	748021.500	12576824.000	98.9697
5		9.137	734.525	9428.700	0.0742
6		10.395	39.964	513.702	0.0040
7		10.875	247.083	4196.156	0.0330
8		11.680	188.055	3835.099	0.0302
总计			756047.736	12707745.266	100.0000

The HPLC spectrum of **CU-1**8

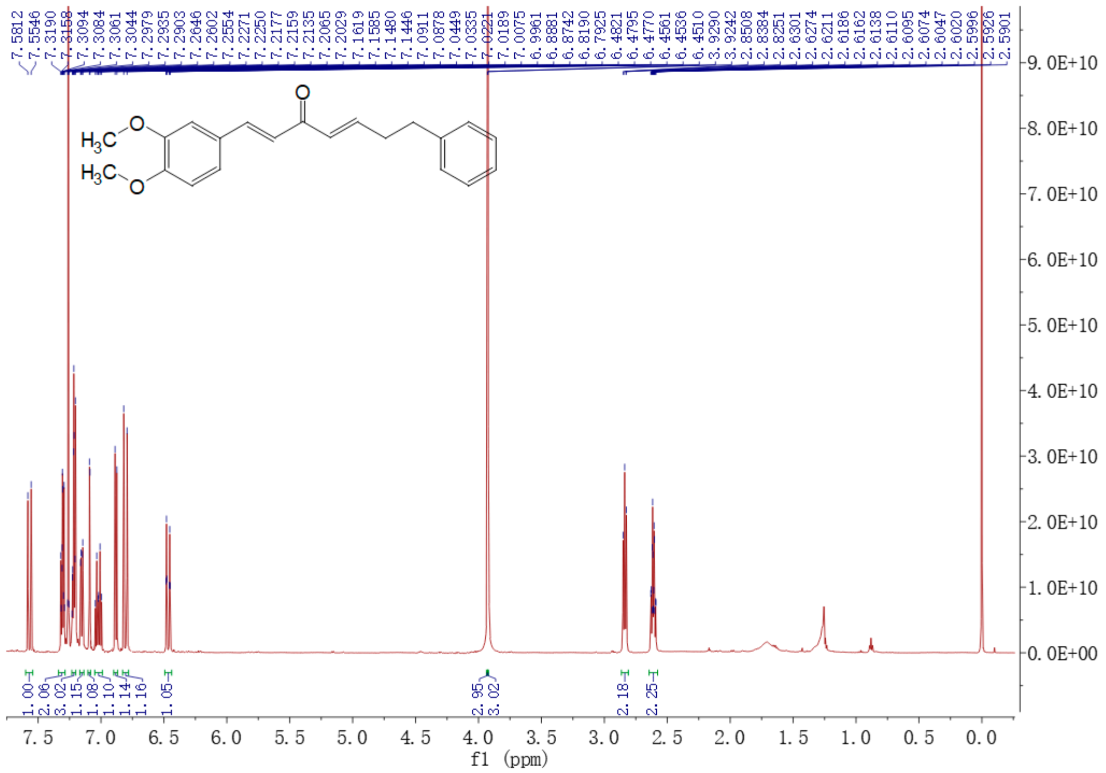

^{1}H NMR spectrum of **CU-19** (600 MHz, $CDCl_3$)

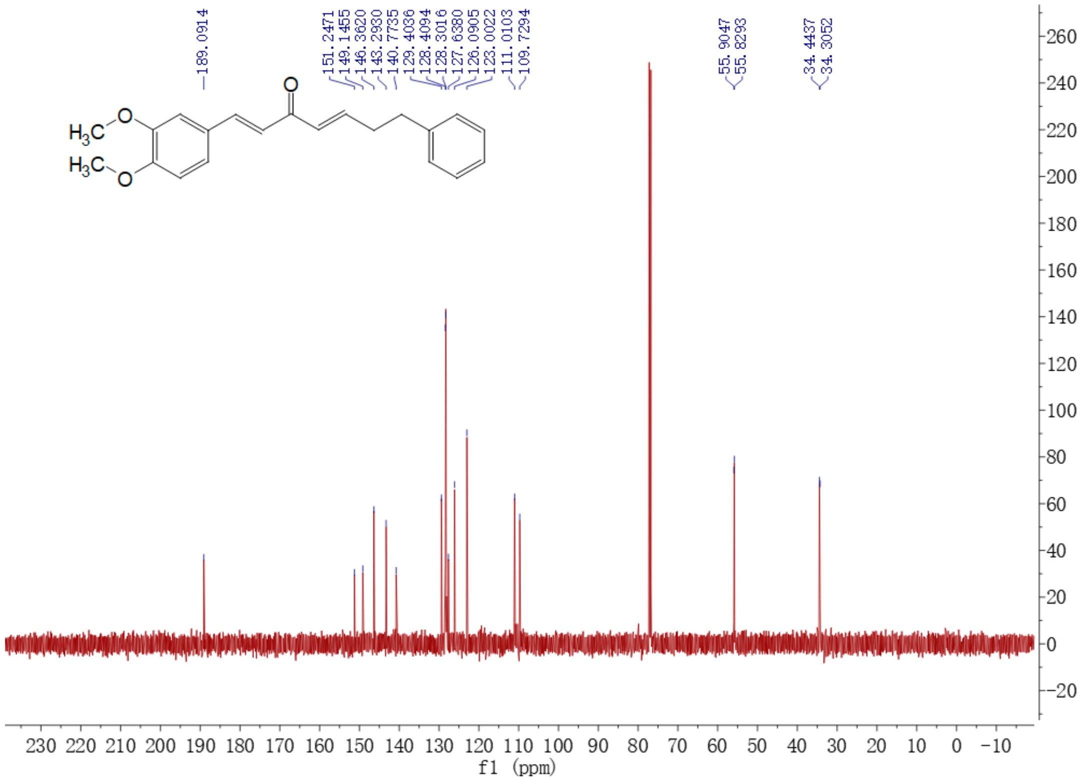

^{13}C NMR spectrum of **CU-19** (151 MHz, $CDCl_3$)

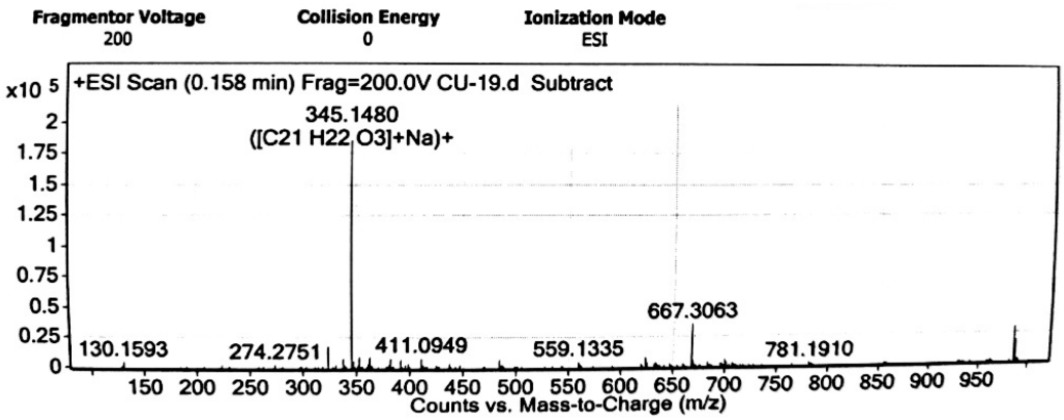

HRMS spectrum of **CU-19**

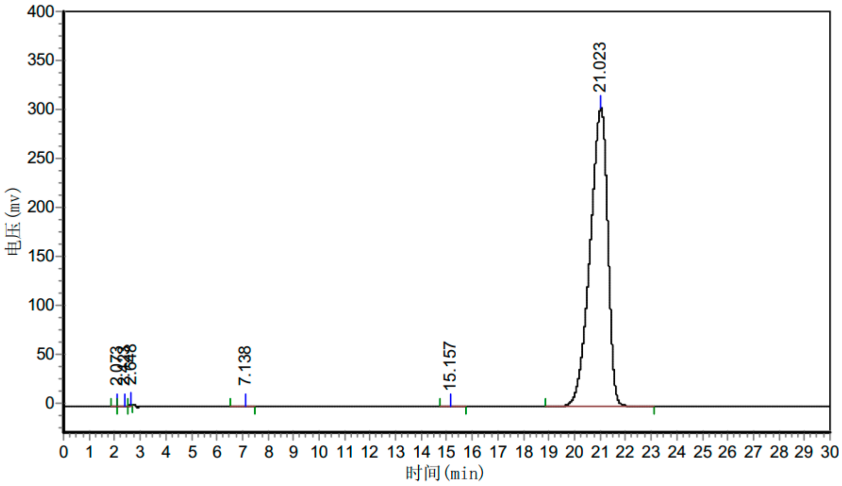

分析结果表

峰号	峰名	保留时间	峰高	峰面积	含量
1		2.073	100.034	605.651	0.0041
2		2.423	774.456	7857.278	0.0529
3		2.648	1997.720	18678.699	0.1258
4		7.138	113.700	3017.489	0.0203
5		15.157	86.438	2275.711	0.0153
6		21.023	306195.469	14812987.000	99.7815
总计			309267.817	14845421.828	100.0000

The HPLC spectrum of **CU-19**

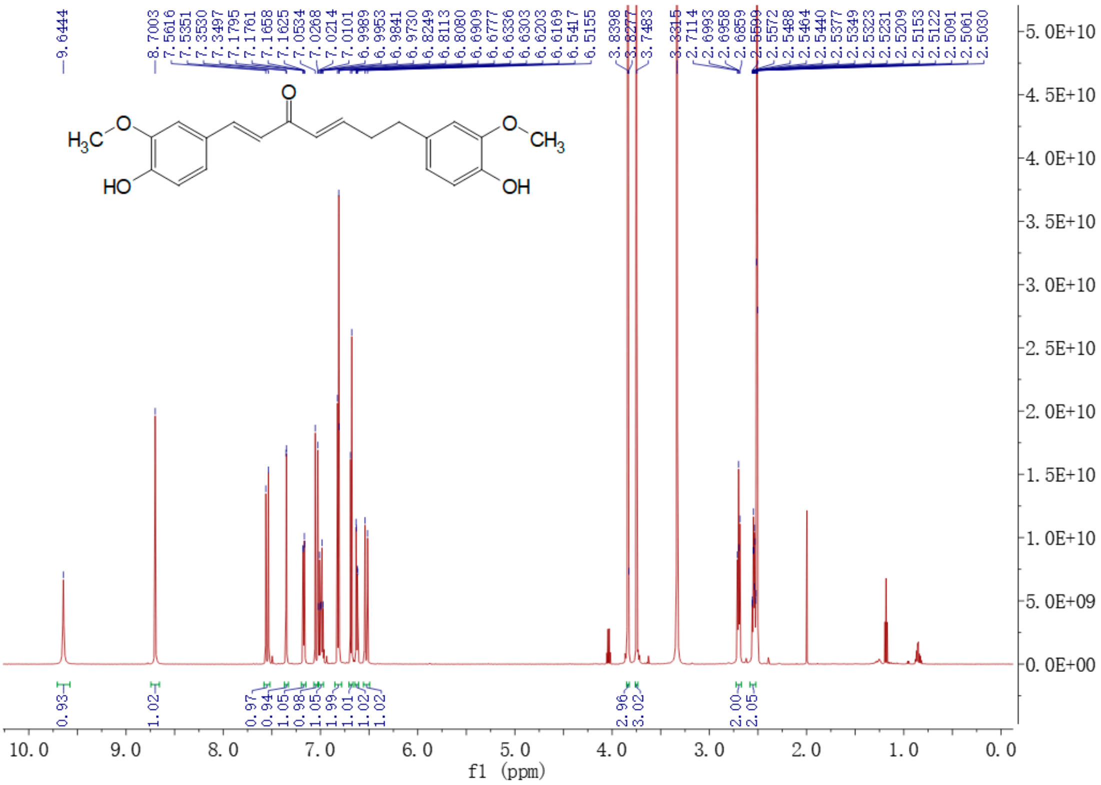

^{1}H NMR spectrum of **CU-20** (600 MHz, DMSO-d_6)

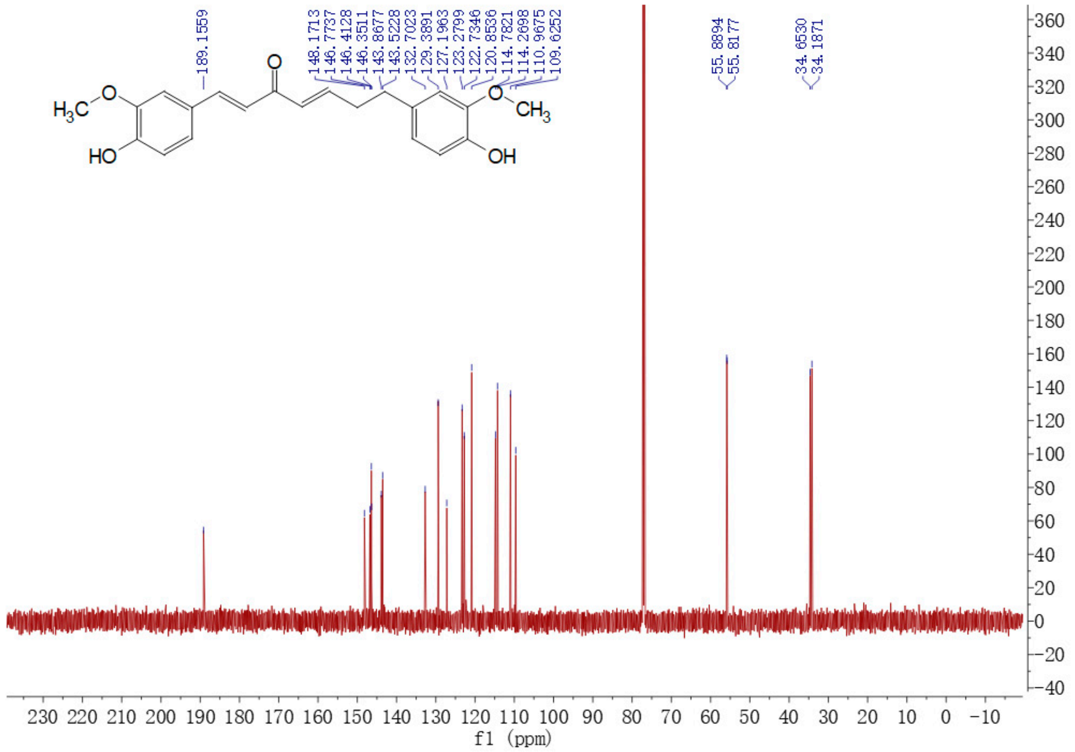

^{13}C NMR spectrum of **CU-20** (151 MHz, $CDCl_3$)

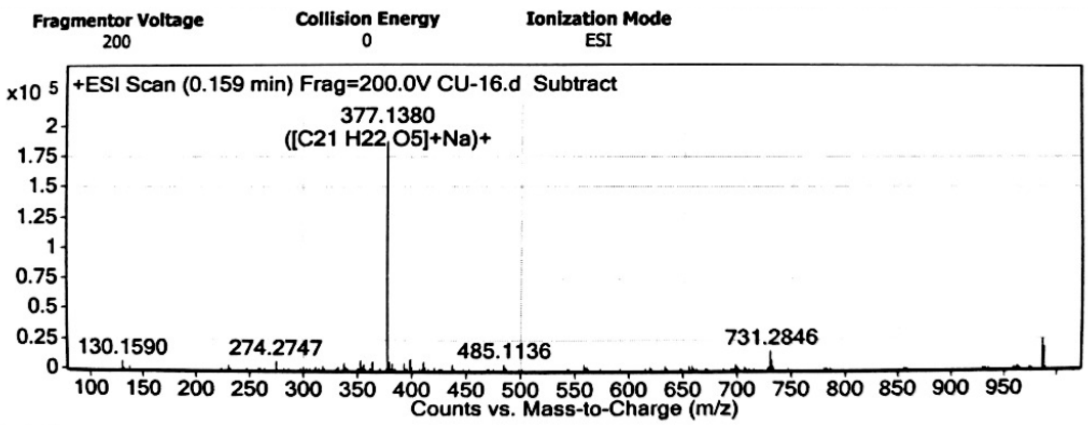

HRMS spectrum of **CU-20**

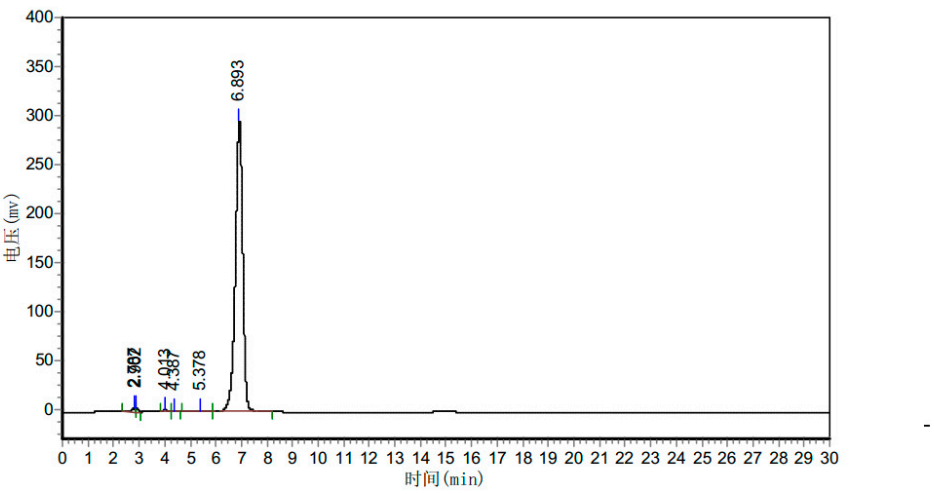

分析结果表

峰号	峰名	保留时间	峰高	峰面积	含量
1		2.787	3385.985	44478.063	0.5084
2		2.902	3399.358	22977.336	0.2627
3		4.013	1736.195	29728.010	0.3398
4		4.387	765.546	9617.381	0.1099
5		5.378	762.724	25239.002	0.2885
6		6.893	316028.875	8616037.000	98.4906
总计			326078.683	8748076.791	100.0000

The HPLC spectrum of **CU-20**

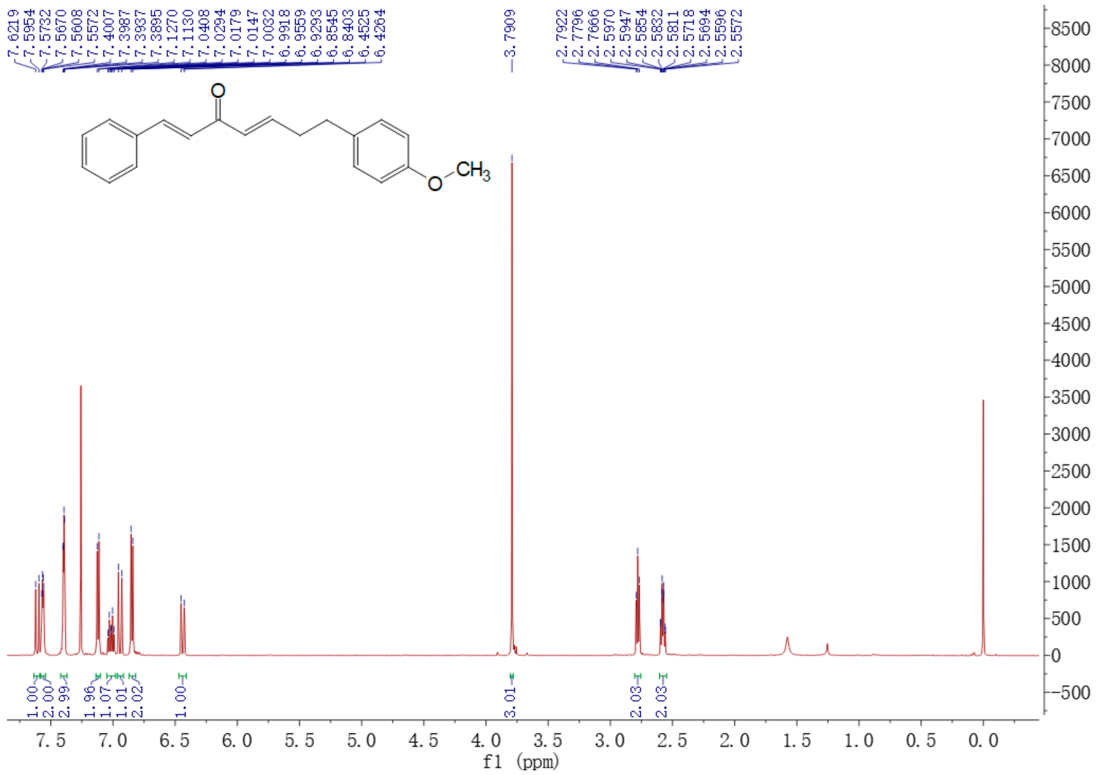

^{1}H NMR spectrum of **CU-21** (600 MHz, DMSO-d_6)

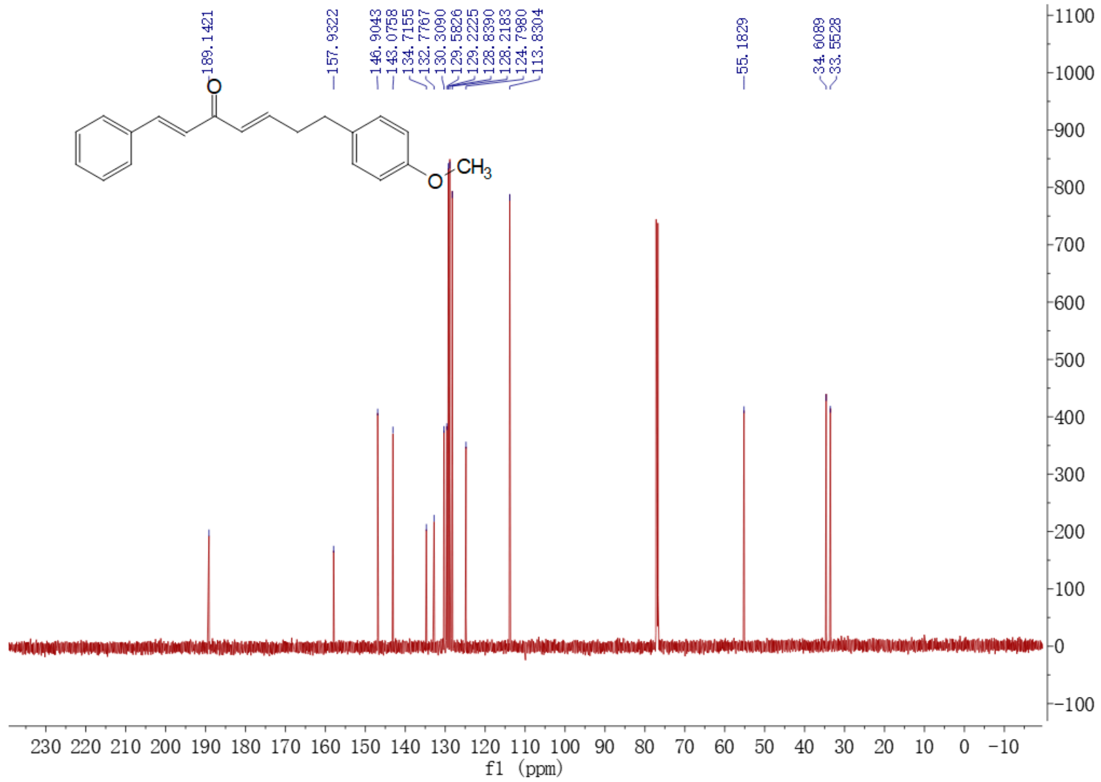

^{13}C NMR spectrum of **CU-21** (151 MHz, $CDCl_3$)

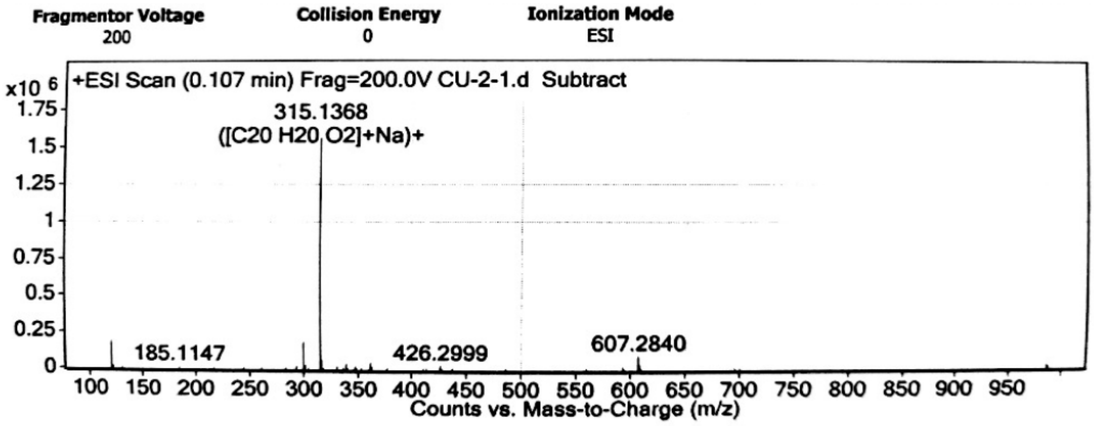

HRMS spectrum of **CU-21**

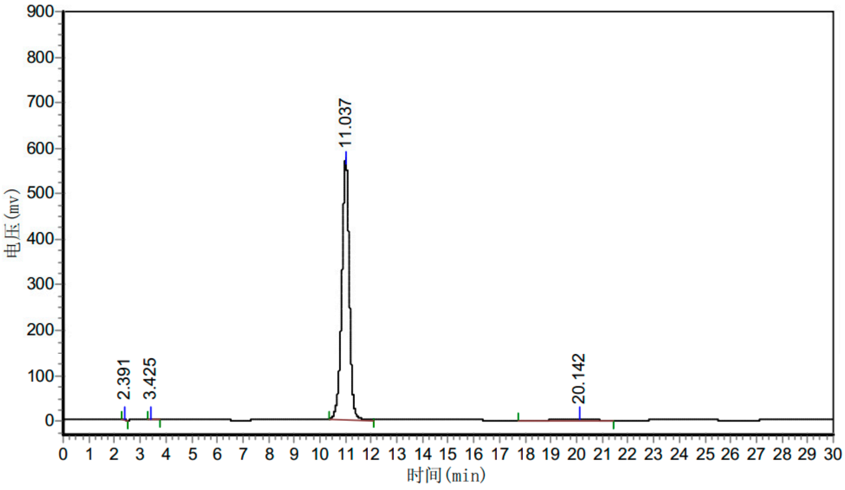

分析结果表

峰号	峰名	保留时间	峰高	峰面积	含量
1		2.391	3002.290	25996.801	0.2181
2		3.425	2300.696	23682.129	0.1987
3		11.037	572452.625	11759446.000	98.6684
4		20.142	1761.638	109027.969	0.9148
总计			579517.249	11918152.898	100.0000

The HPLC spectrum of **CU-21**

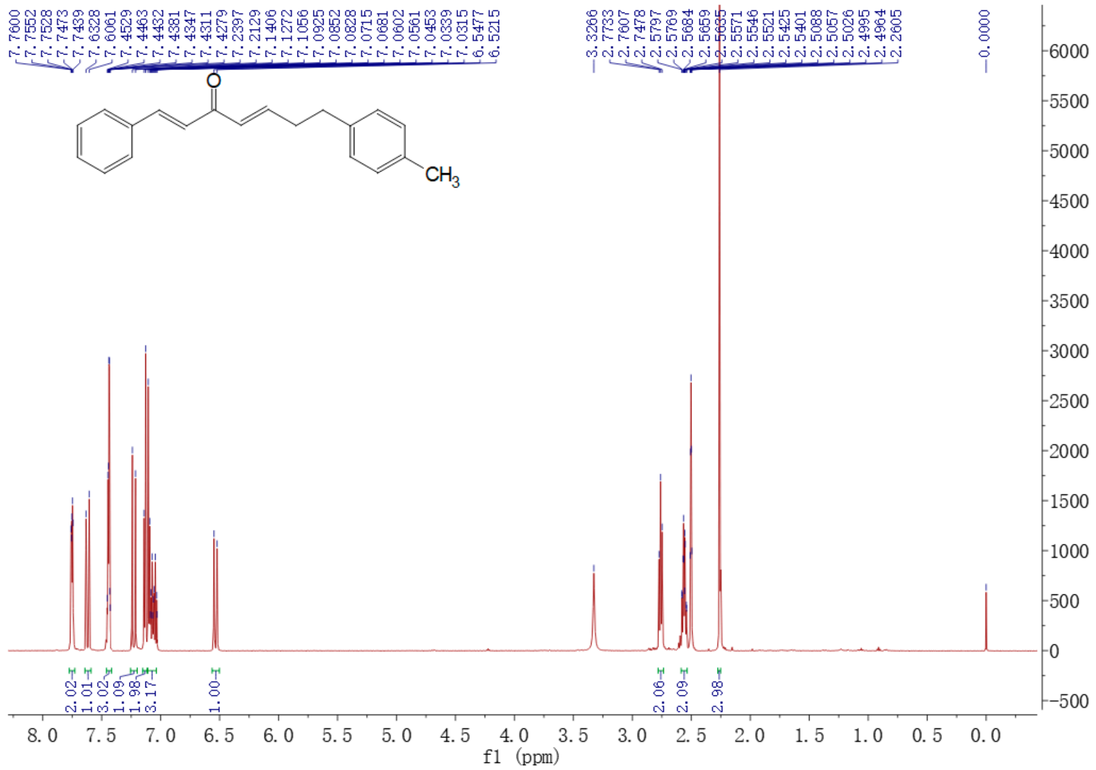

^{1}H NMR spectrum of **CU-22** (600 MHz, DMSO-d_6)

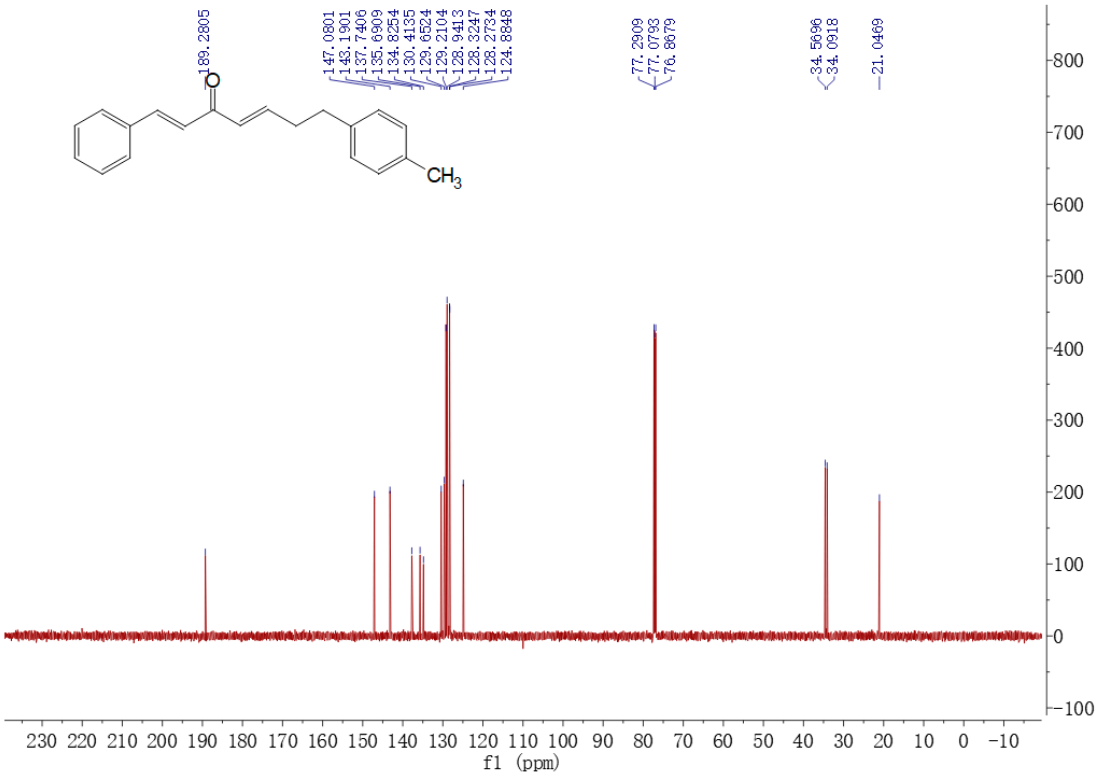

^{13}C NMR spectrum of **CU-22** (151 MHz, $CDCl_3$)

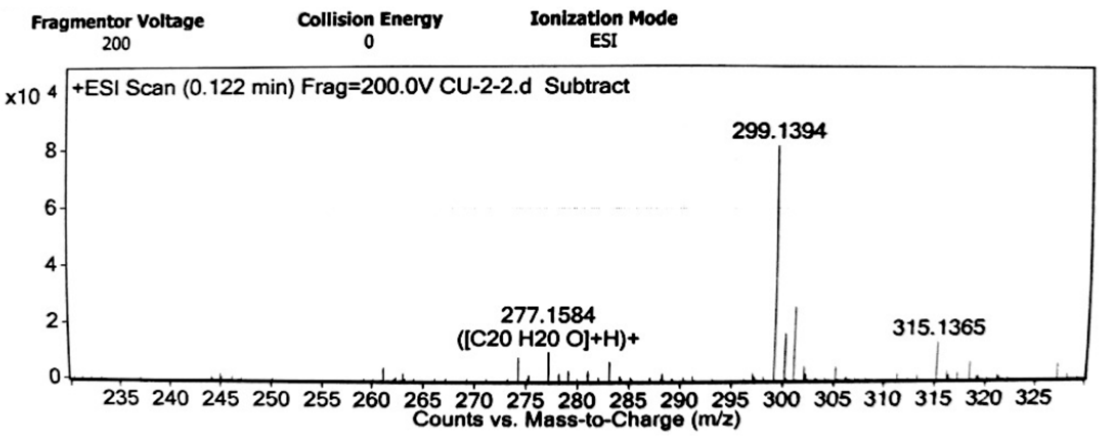

HRMS spectrum of **CU-22**

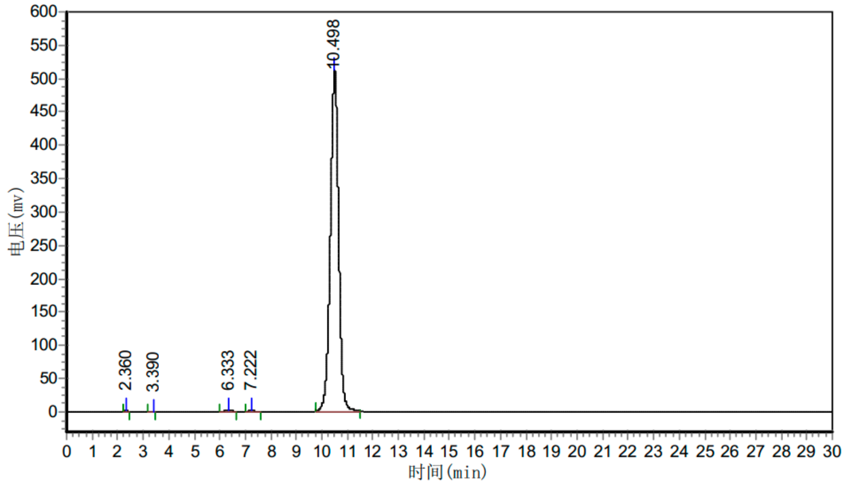

分析结果表

峰号	峰名	保留时间	峰高	峰面积	含量
1		2. 360	2905. 563	27012. 738	0. 2292
2		3. 390	321. 310	3259. 391	0. 0277
3		6. 333	3124. 772	55271. 230	0. 4690
4		7. 222	1659. 751	34418. 188	0. 2921
5		10. 498	512449. 031	11664626. 000	98. 9820
总计			520460. 428	11784587. 547	100. 0000

The HPLC spectrum of **CU-22**

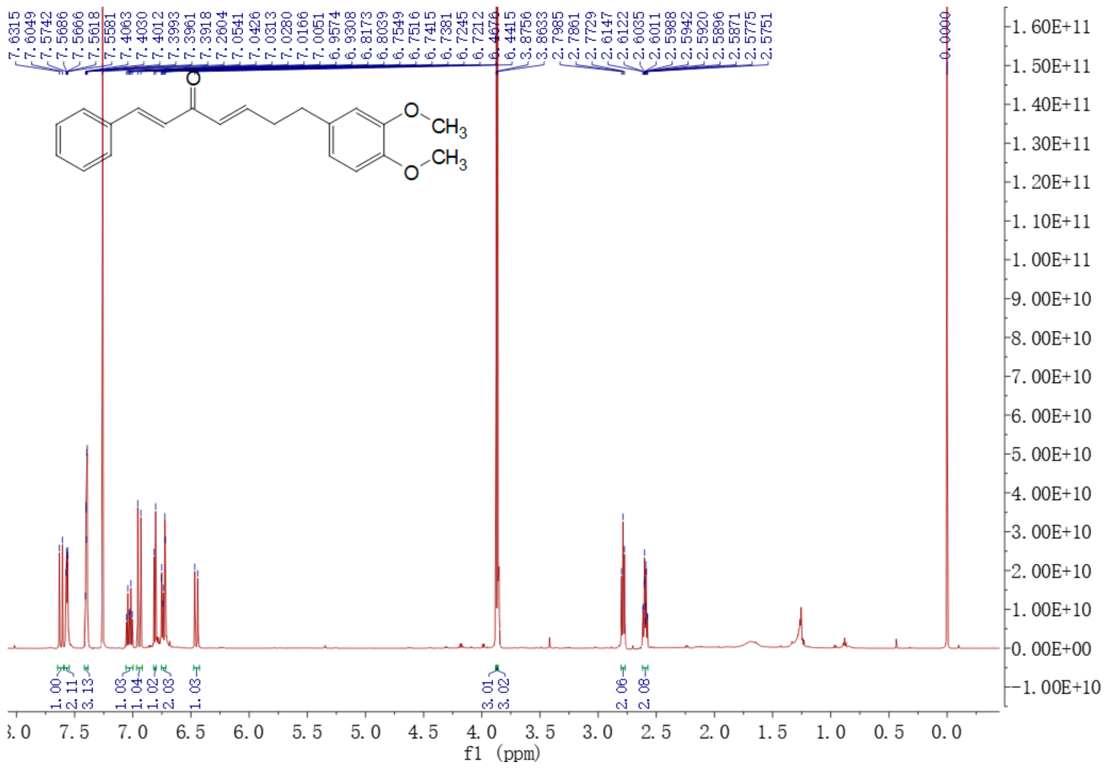

^{1}H NMR spectrum of **CU-23** (600 MHz, $CDCl_3$)

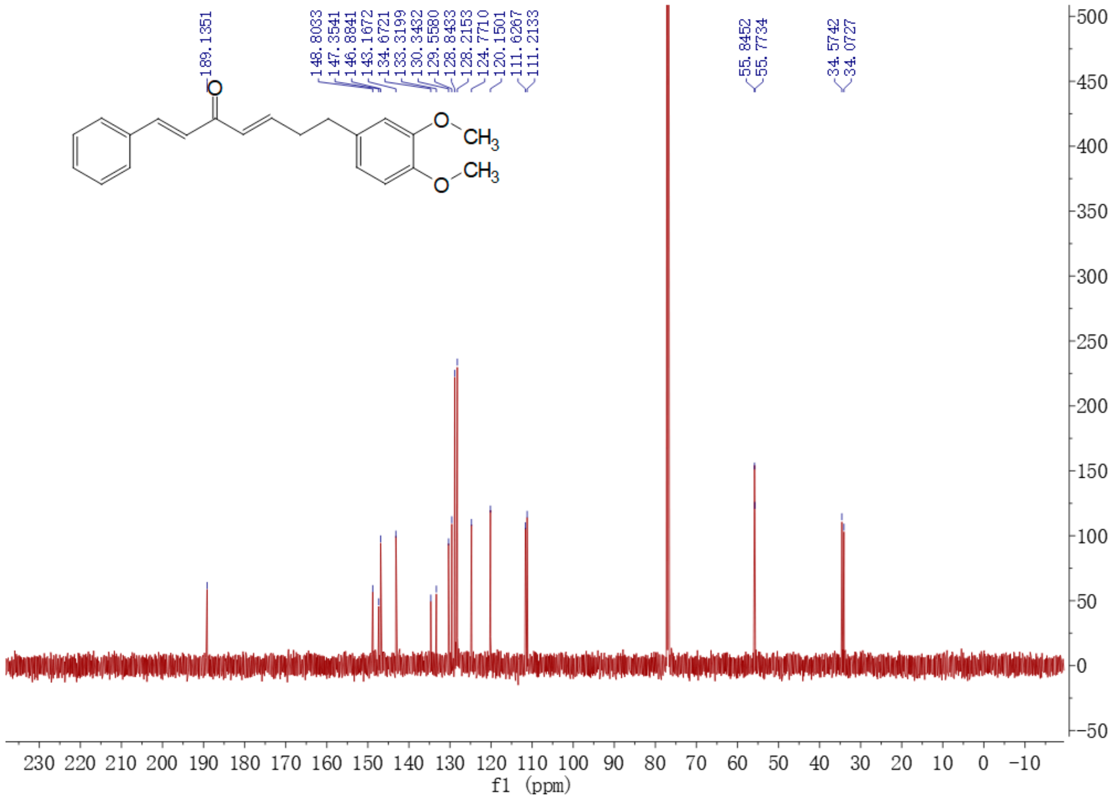

^{13}C NMR spectrum of **CU-23** (151 MHz, $CDCl_3$)

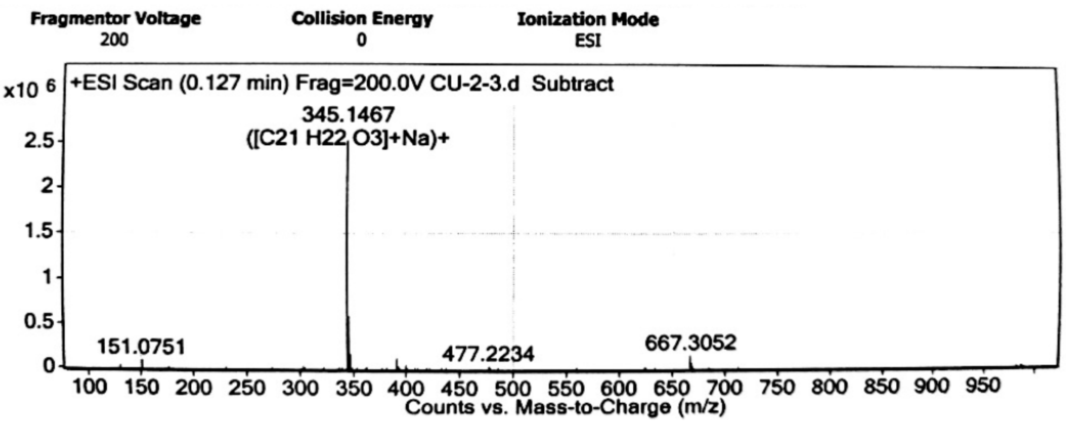

HRMS spectrum of **CU-23**

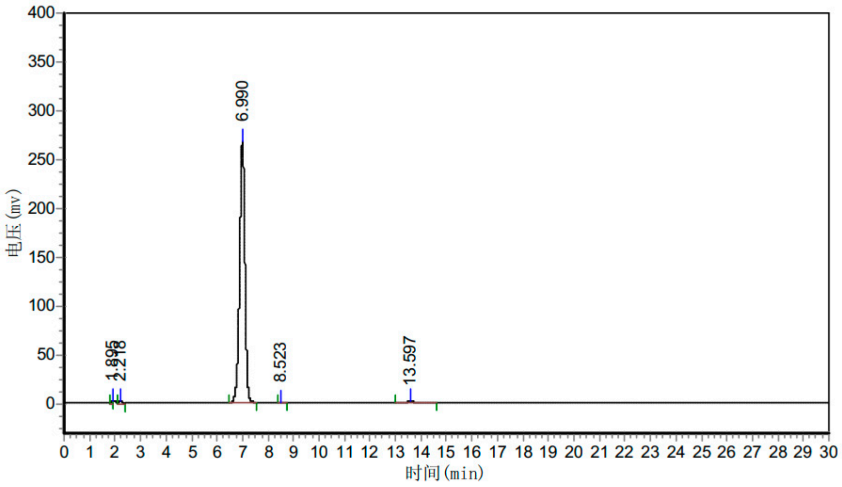

分析结果表

峰号	峰名	保留时间	峰高	峰面积	含量
1		1.895	1713.736	11773.936	0.2966
2		2.218	3007.332	28375.893	0.7149
3		6.990	268341.344	3882909.750	97.8263
4		8.523	67.161	806.950	0.0203
5		13.597	1459.562	45320.070	1.1418
总计			274589.134	3969186.599	100.0000

The HPLC spectrum of **CU-23**

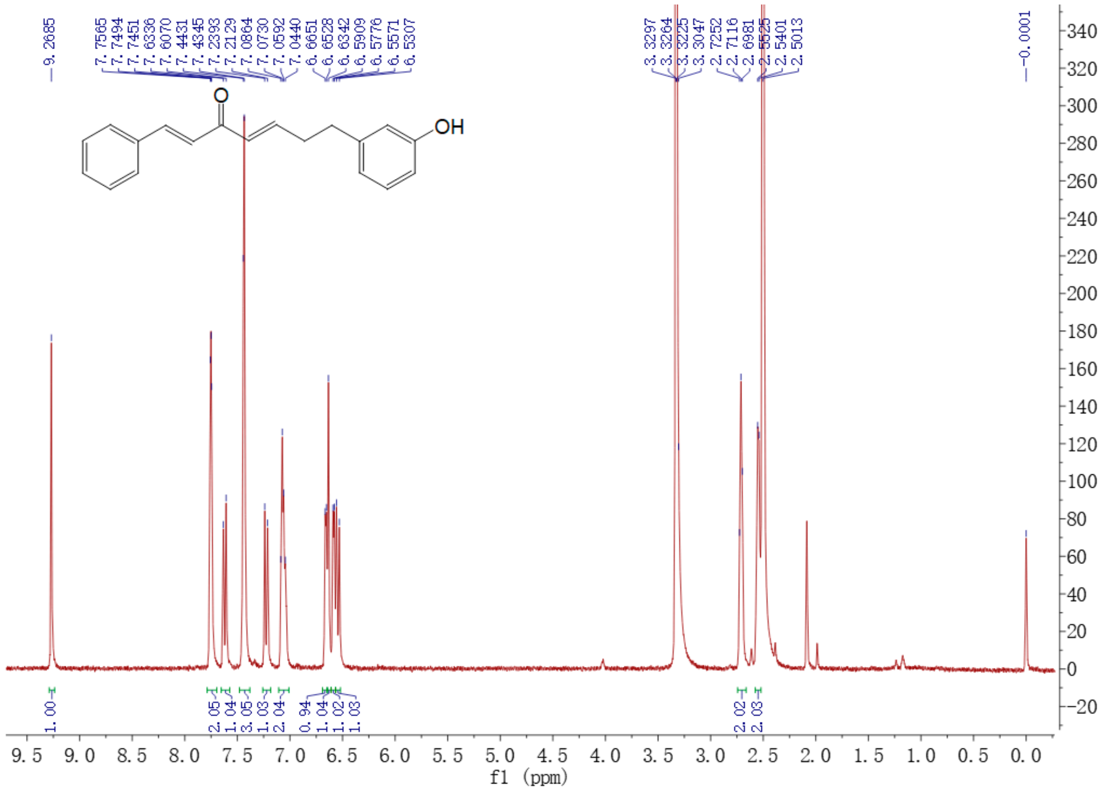

^{1}H NMR spectrum of **CU-24** (600 MHz, DMSO-d_6)

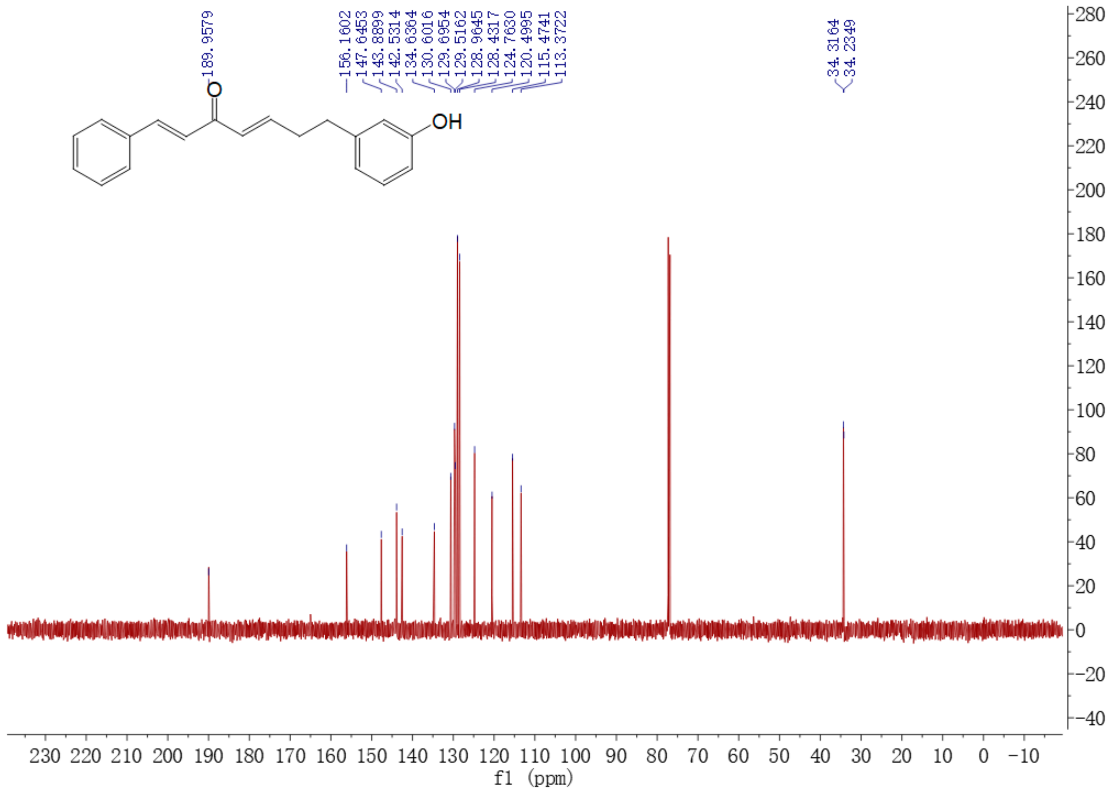

^{13}C NMR spectrum of **CU-24** (151 MHz, $CDCl_3$)

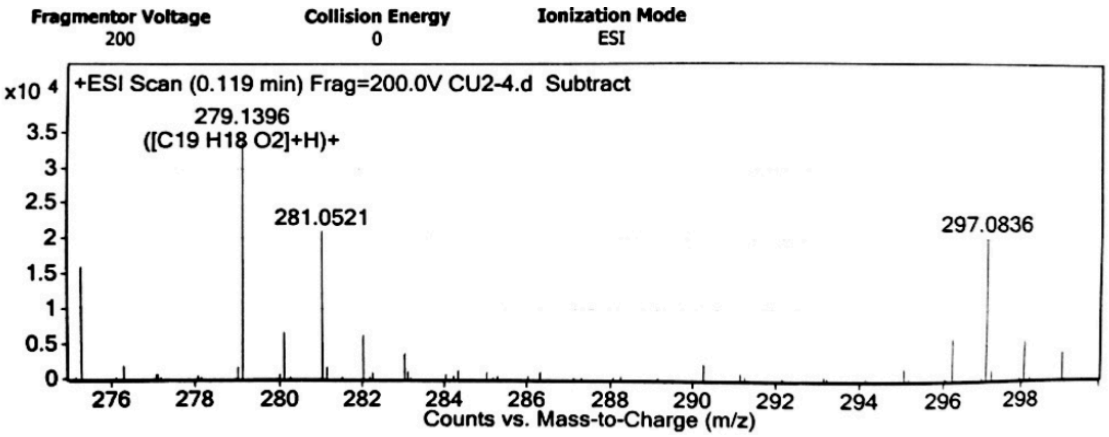

HRMS spectrum of **CU-24**

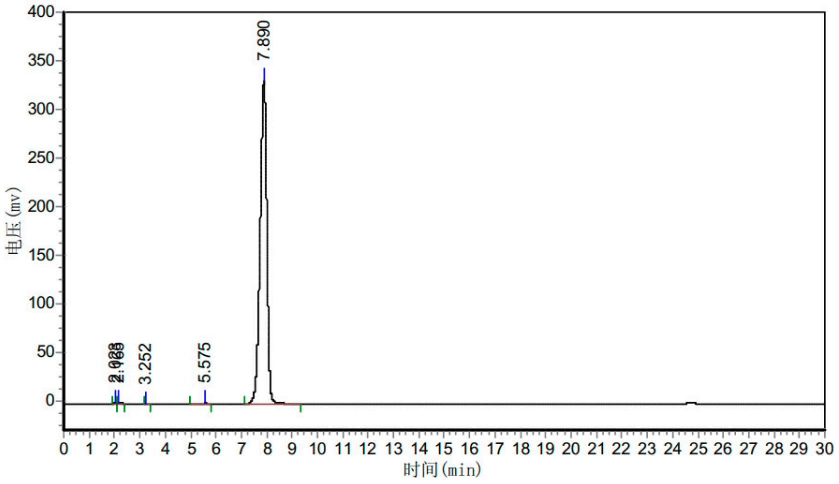

分析结果表

峰号	峰名	保留时间	峰高	峰面积	含量
1		2.023	1400.328	10989.504	0.1784
2		2.165	2311.156	26861.221	0.4361
3		3.252	241.002	2239.907	0.0364
4		5.575	595.056	6661.798	0.1081
5		7.890	332275.406	6113292.000	99.2410
总计			336822.948	6160044.429	100.0000

The HPLC spectrum of **CU-24**

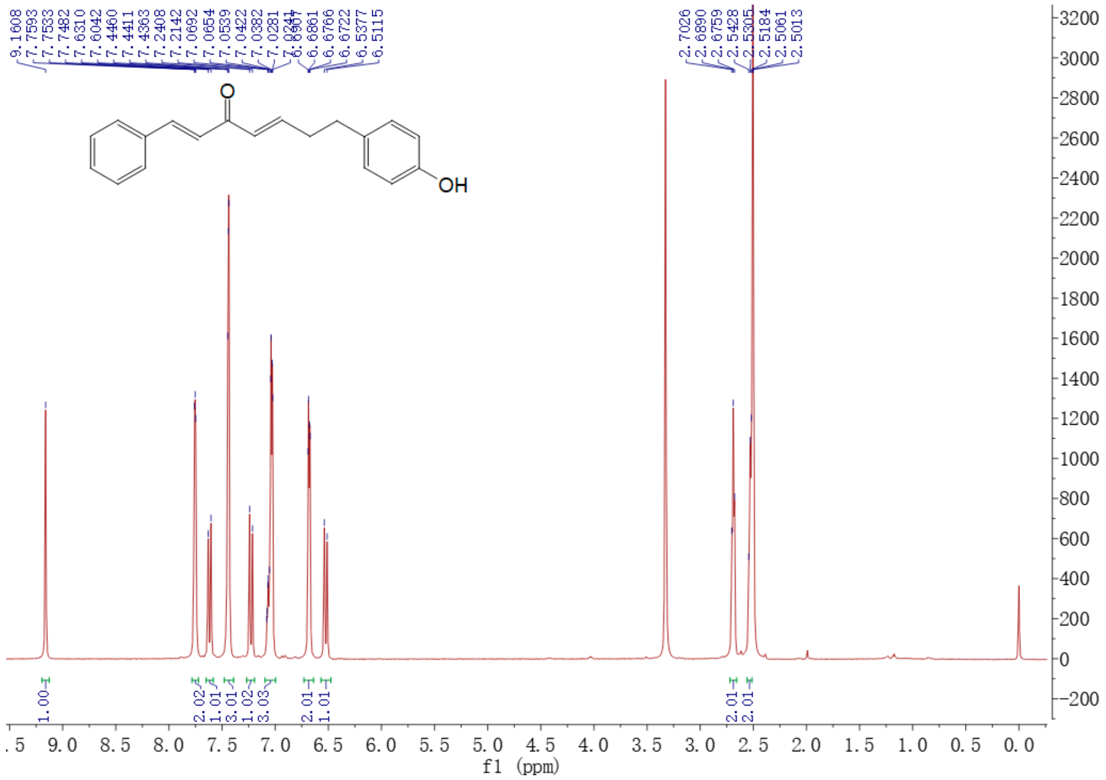

^{1}H NMR spectrum of **CU-25** (600 MHz, DMSO-d_6)

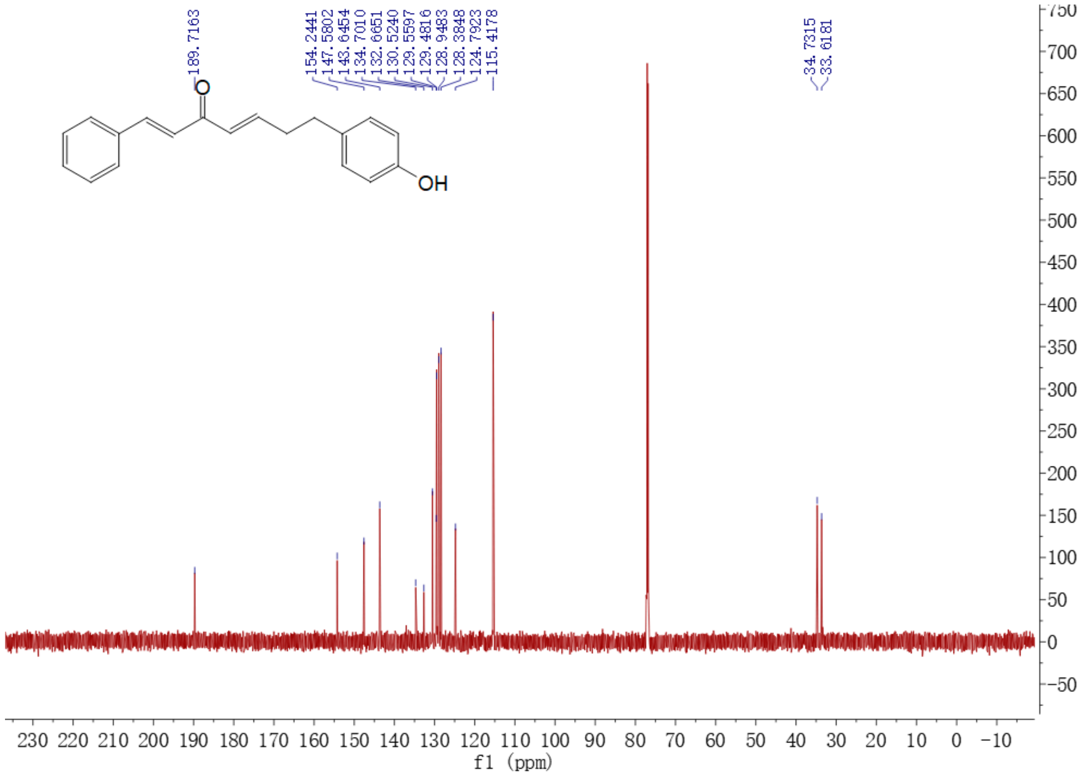

^{13}C NMR spectrum of **CU-25** (151 MHz, $CDCl_3$)

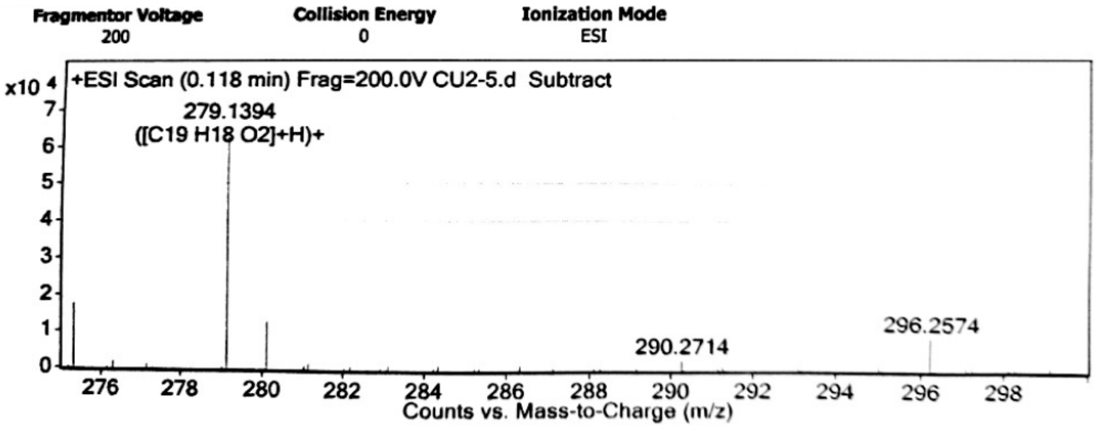

HRMS spectrum of **CU-25**

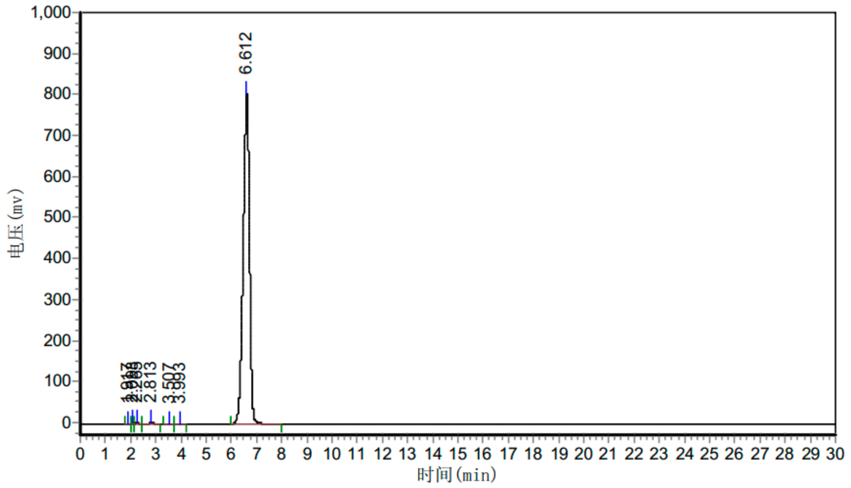

分析结果表

峰号	峰名	保留时间	峰高	峰面积	含量
1		1.917	782.113	8296.820	0.0585
2		2.093	1728.069	11892.307	0.0839
3		2.265	2652.758	24651.416	0.1739
4		2.813	5149.940	63053.742	0.4448
5		3.507	1232.668	21451.383	0.1513
6		3.993	977.338	23172.100	0.1635
7		6.612	805443.438	14022141.000	98.9240
总计			817966.324	14174658.768	100.0000

The HPLC spectrum of **CU-25**

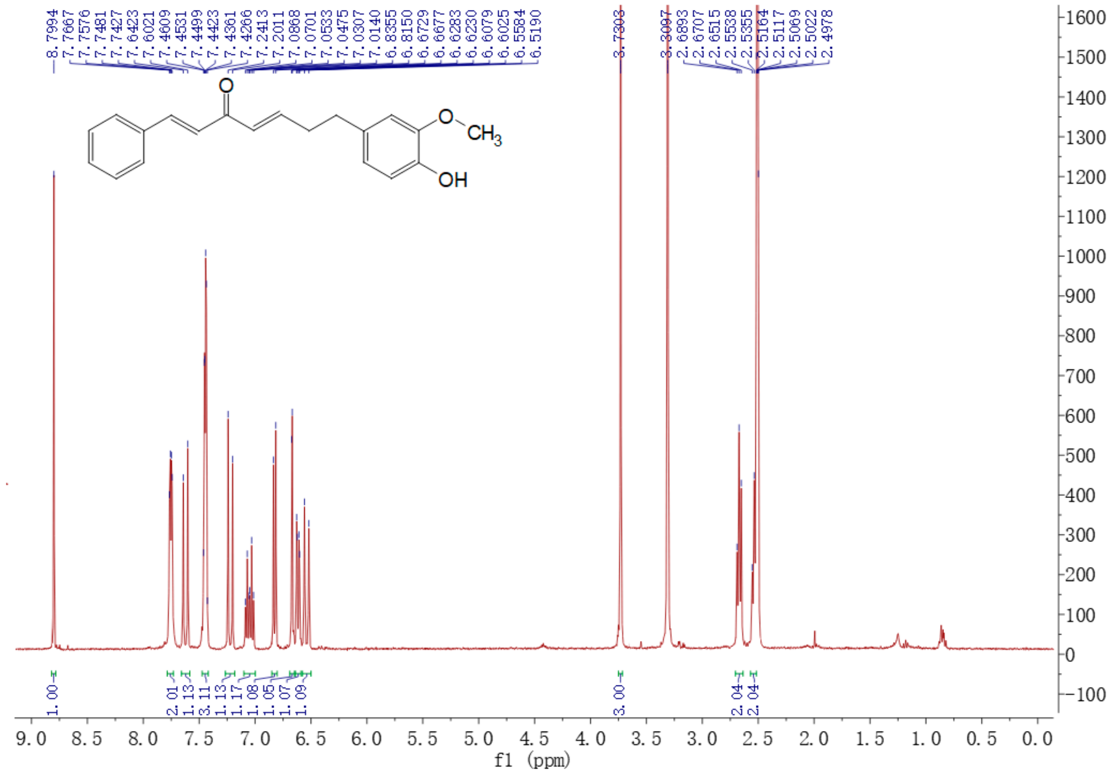

^{1}H NMR spectrum of **CU-26** (600 MHz, DMSO-d_6)

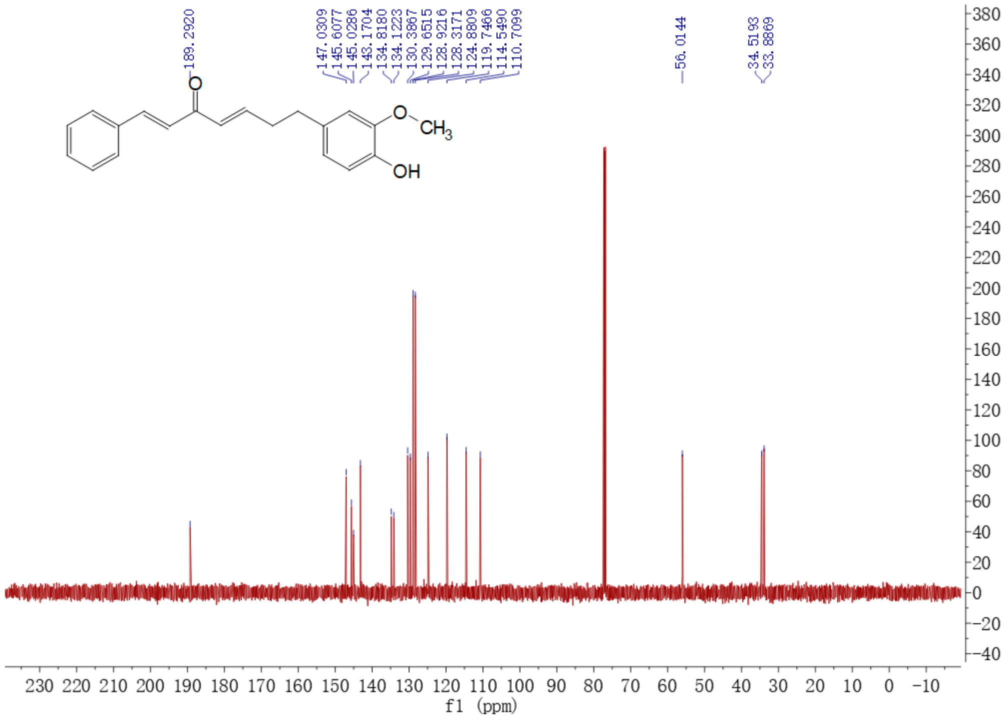

^{13}C NMR spectrum of **CU-26** (151 MHz, $CDCl_3$)

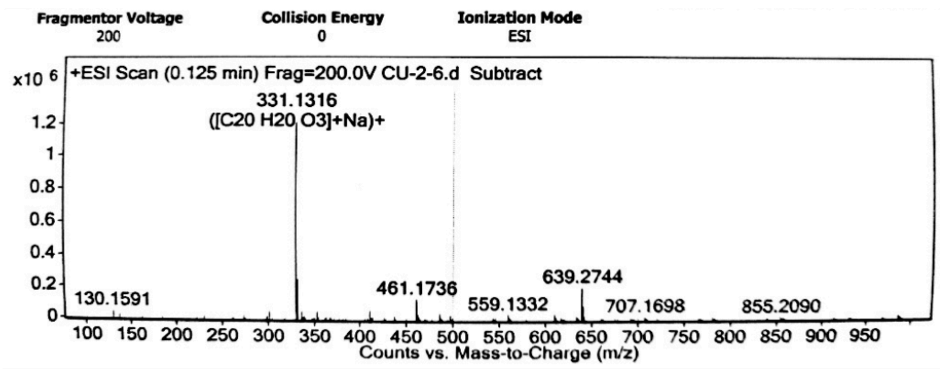

HRMS spectrum of **CU-26**

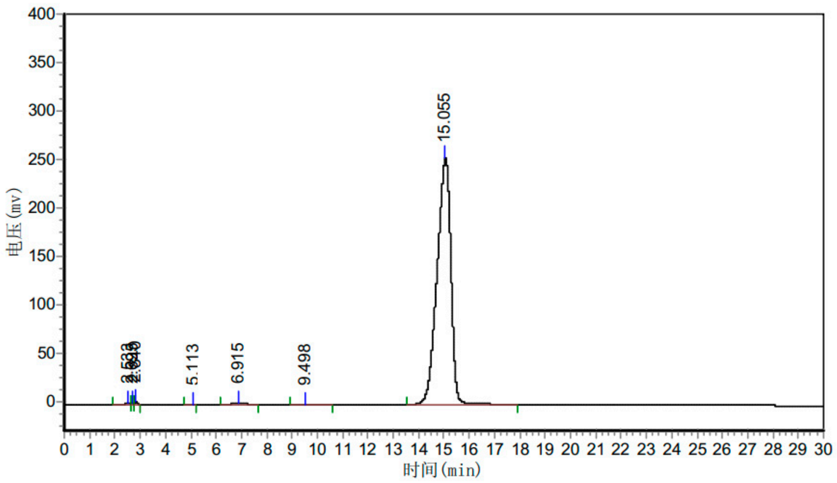

分析结果表

峰号	峰名	保留时间	峰高	峰面积	含量
1		2.533	1775.016	25576.943	0.2628
2		2.695	1568.596	8098.390	0.0832
3		2.840	3193.023	29132.199	0.2994
4		5.113	143.329	2144.889	0.0220
5		6.915	574.446	20796.652	0.2137
6		9.498	252.002	8483.975	0.0872
7		15.055	254276.422	9637396.000	99.0317
**总计**			261782.833	9731629.049	100.0000

The HPLC spectrum of **CU-26**

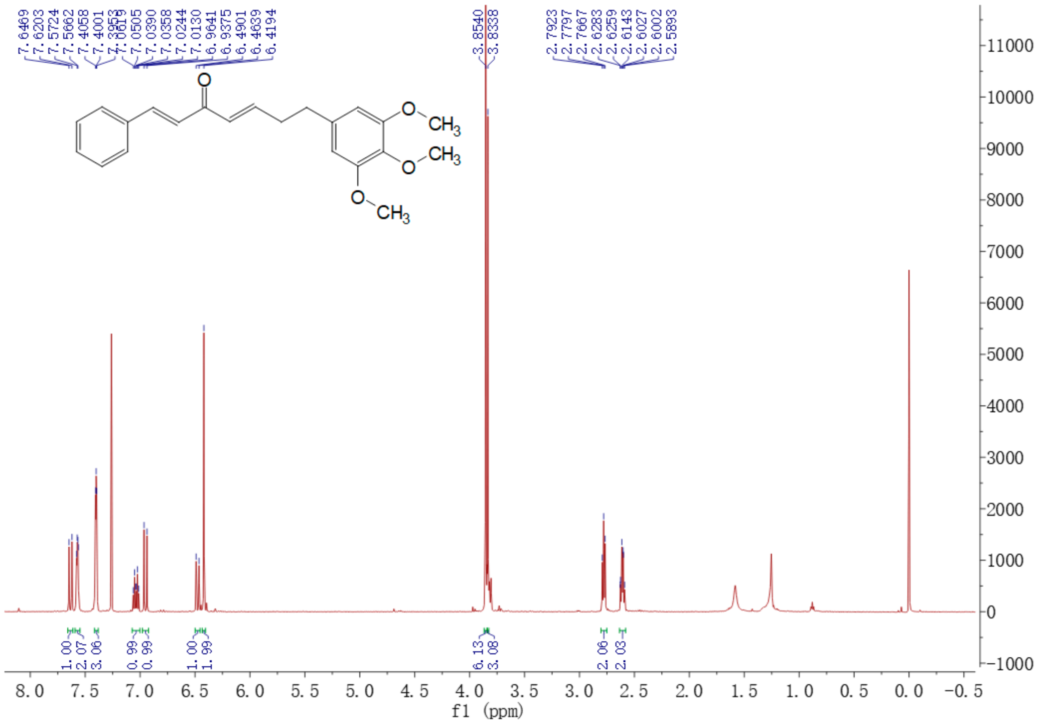

^{1}H NMR spectrum of **CU-27** (600 MHz, $CDCl_3$)

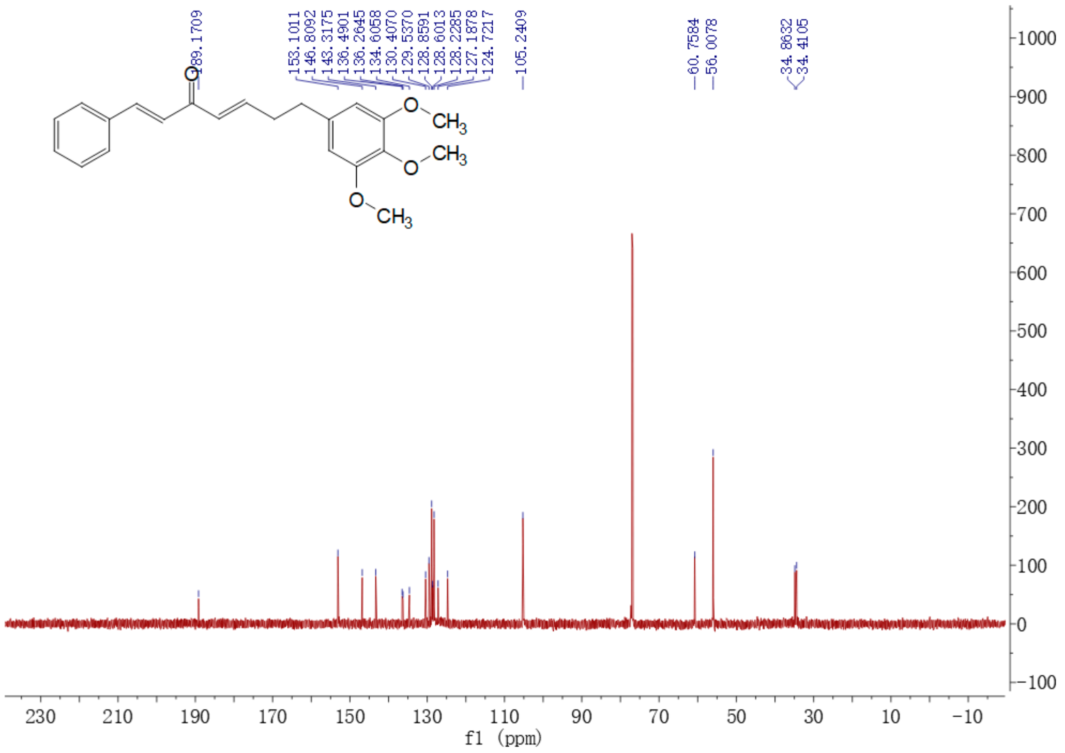

^{13}C NMR spectrum of **CU-27** (151 MHz, $CDCl_3$)

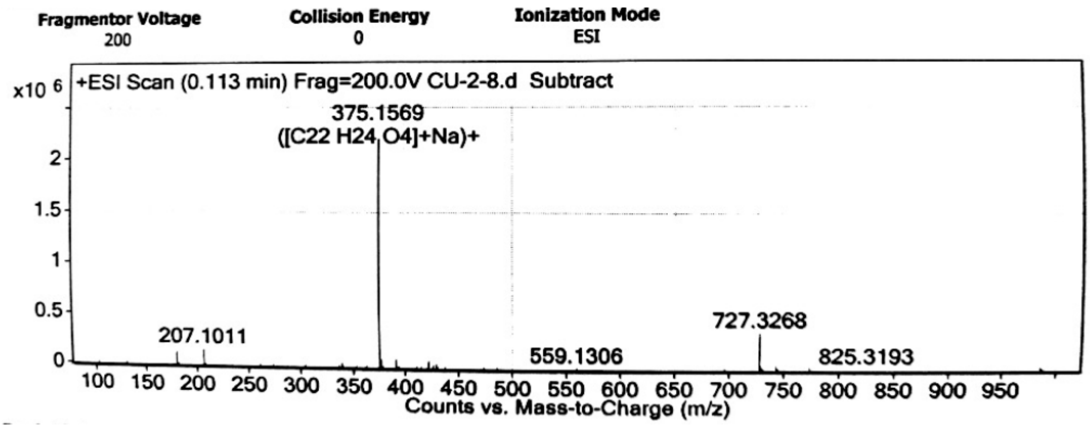

HRMS spectrum of **CU-27**

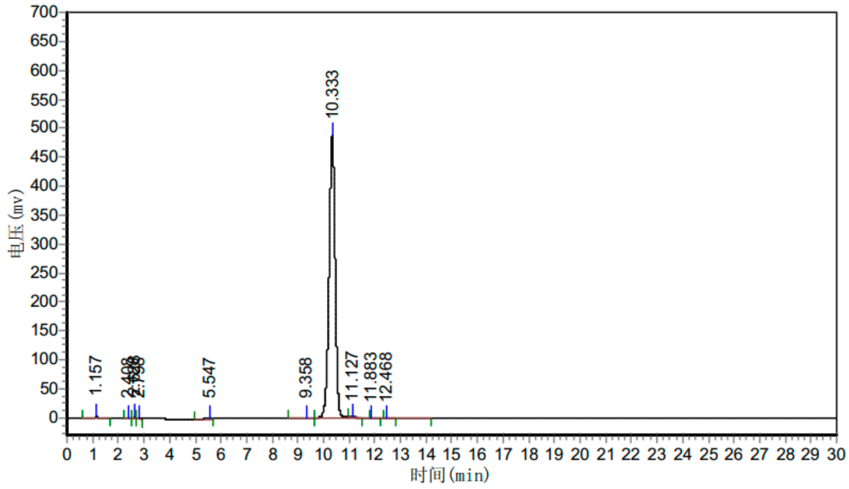

分析结果表

峰号	峰名	保留时间	峰高	峰面积	含量
1		1. 157	1391. 207	26807. 098	0. 3168
2		2. 408	1385. 321	16413. 619	0. 1940
3		2. 628	2304. 184	19312. 771	0. 2283
4		2. 798	2409. 861	20278. 156	0. 2397
5		5. 547	347. 754	8042. 631	0. 0951
6		9. 358	1856. 601	83053. 844	0. 9816
7		10. 333	488781. 063	8268843. 500	97. 7295
8		11. 127	1052. 964	15643. 999	0. 1849
9		11. 883	84. 590	1271. 751	0. 0150
10		12. 468	68. 270	1284. 000	0. 0152
**总计**			499681. 815	8460951. 370	100. 0000

The HPLC spectrum of **CU-27**

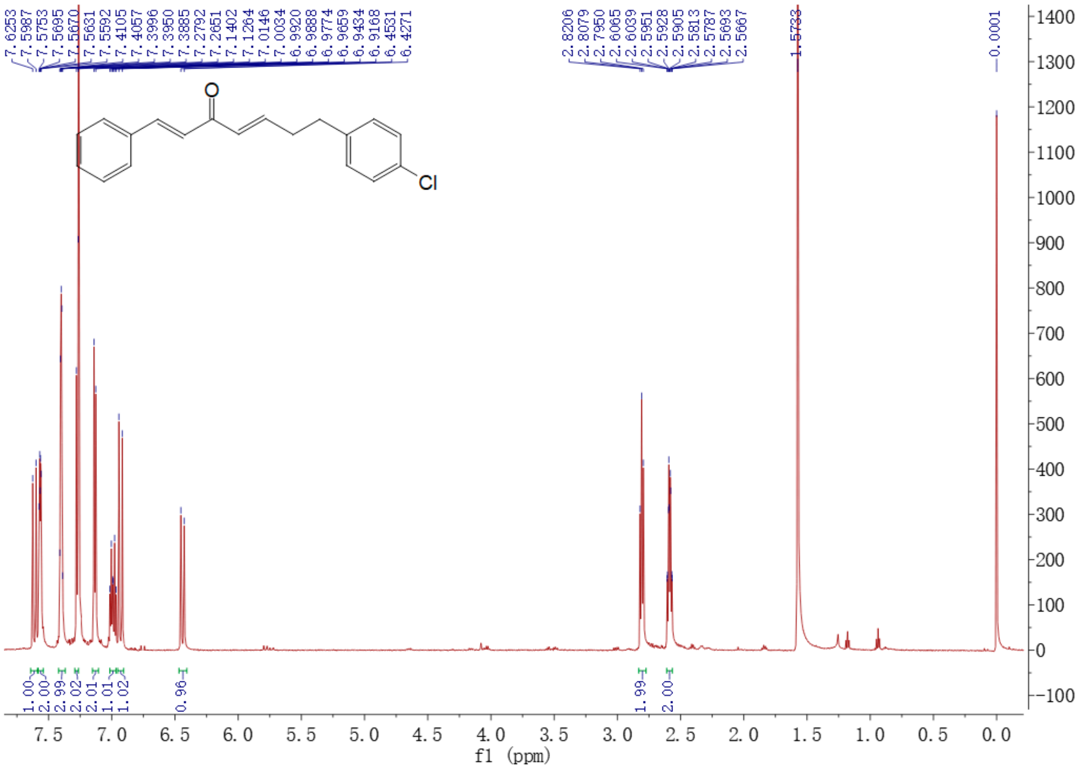

^{1}H NMR spectrum of **CU-28** (600 MHz, $CDCl_3$)

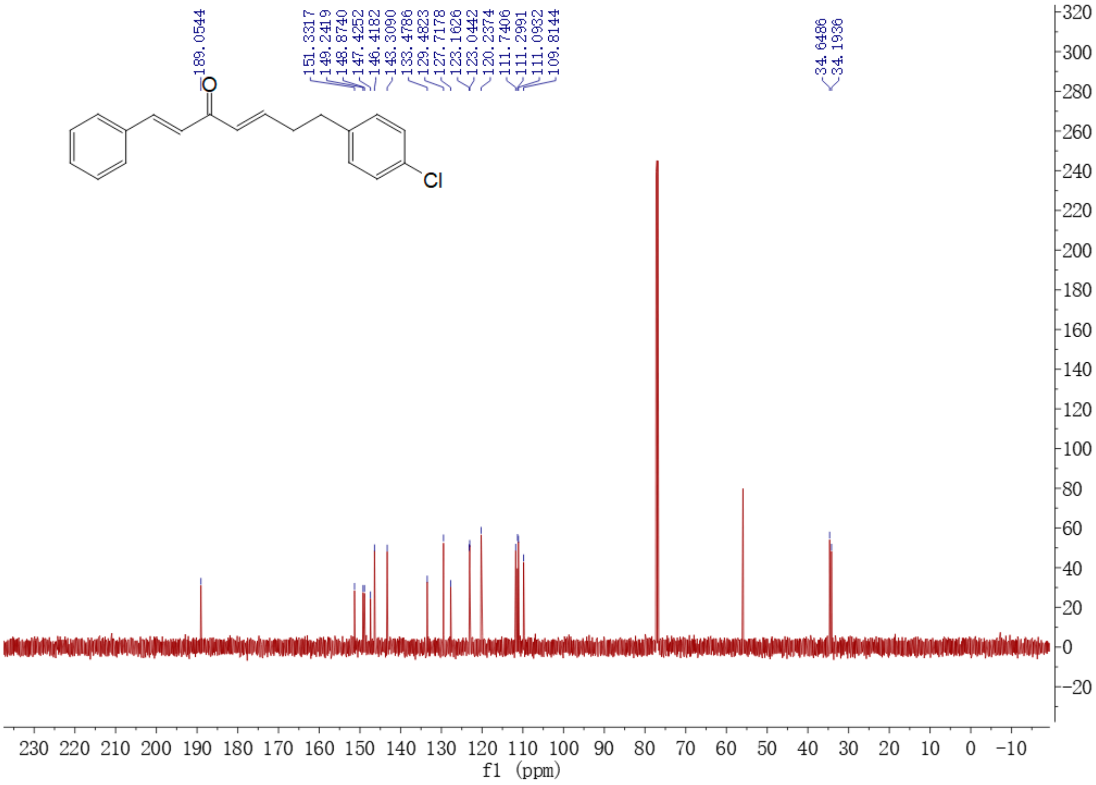

^{13}C NMR spectrum of **CU-28** (151 MHz, $CDCl_3$)

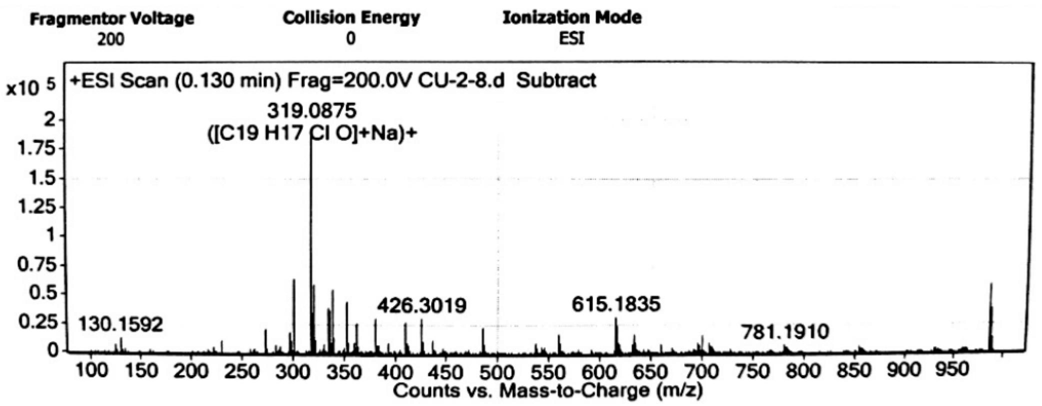

HRMS spectrum of **CU-28**

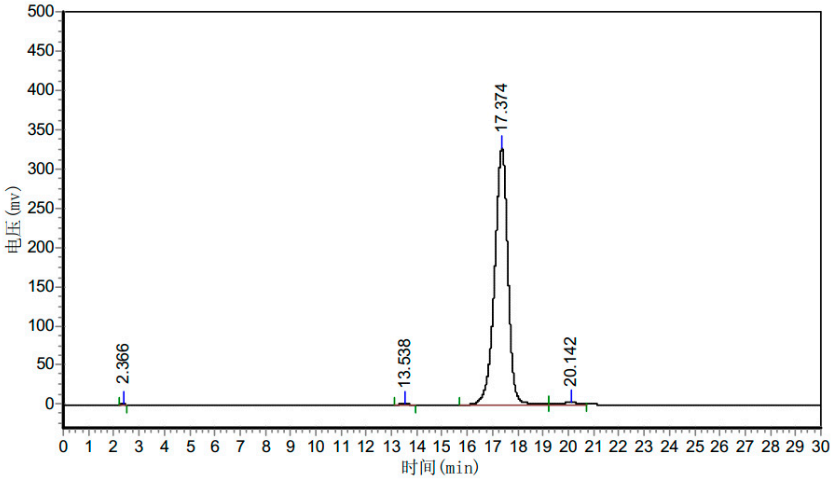

分析结果表

峰号	峰名	保留时间	峰高	峰面积	含量
1		2.366	2473.032	23140.064	0.1888
2		13.538	1237.150	37409.094	0.3052
3		17.374	327512.375	11997911.000	97.8831
4		20.142	3512.324	177405.109	1.4473
5		31.581	24.725	1673.784	0.0137
6		36.917	272.984	19852.699	0.1620
总计			335032.591	12257391.751	100.0000

The HPLC spectrum of **CU-28**
